# Synthesis and Biological Evaluation of Structurally Varied 5′-/6′-Isonucleosides and Theobromine-Containing *N*-Isonucleosidyl Derivatives

**DOI:** 10.3390/ph12030103

**Published:** 2019-07-02

**Authors:** Nuno M. Xavier, Eduardo C. de Sousa, Margarida P. Pereira, Anne Loesche, Immo Serbian, René Csuk, M. Conceição Oliveira

**Affiliations:** 1 Centro de Química e Bioquímica, Faculdade de Ciências, Universidade de Lisboa, Ed. C8, 5º Piso, Campo Grande, 1749-016 Lisboa, Portugal; 2 Centro de Química Estrutural, Faculdade de Ciências, Universidade de Lisboa, Ed. C8, 5º Piso, Campo Grande, 1749-016 Lisboa, Portugal; 3 Bereich Organische Chemie, Martin-Luther-Universität Halle-Wittenberg, Kurt-Mothes-Str. 2, D-06120 Halle (Saale), Germany; 4 Centro de Química Estrutural, Instituto Superior Técnico, Universidade de Lisboa, Av. Rovisco Pais, 1049-001 Lisboa, Portugal

**Keywords:** isonucleosides, theobromine, Mitsunobu reaction, cholinesterase inhibitors

## Abstract

Isonucleosides are rather stable regioisomeric analogs of nucleosides with broad therapeutic potential. We have previously demonstrated the ability of 5′ and 6′-isonucleosides to inhibit the activity of acetylcholinesterase, a major target for Alzheimer’s disease therapy. Continuing with our research on this topic, we report herein on the synthesis and biological evaluation of a variety of novel terminal isonucleosides and theobromine isonucleotide analogs. Xylofuranose-based purine or uracil 5′-isonucleosides and xylofuranos-5′-yl or glucos-6′-yl theobromine derivatives were accessed via Mitsunobu coupling between partially protected xylofuranose or glucofuranose derivatives with a nucleobase using conventional or microwave-assisted heating conditions. Theobromine-containing *N*-isonucleosidyl sulfonamide and phosphoramidate derivatives were synthesized from isonucleosidyl acetate precursors. The most active compounds in the cholinesterase inhibition assays were a glucopyranose-based theobromine isonucleosidyl acetate, acting as a dual inhibitor of acetylcholinesterase (AChE, *K*_i_ = 3.1 µM) and butyrylcholinesterase (BChE, *K*_i_ = 5.4 µM), and a 2-*O*,4-*O*-bis-xylofuranos-5′-yl uracil derivative, which displayed moderate inhibition of AChE (*K*_i_ = 17.5 µM). Docking studies revealed that the active molecules are positioned at the gorge entrance and at the active site of AChE. None of the compounds revealed cytoxic activity to cancer cells as well as to non-malignant mouse fibroblasts.

## 1. Introduction

**Figure 1:**
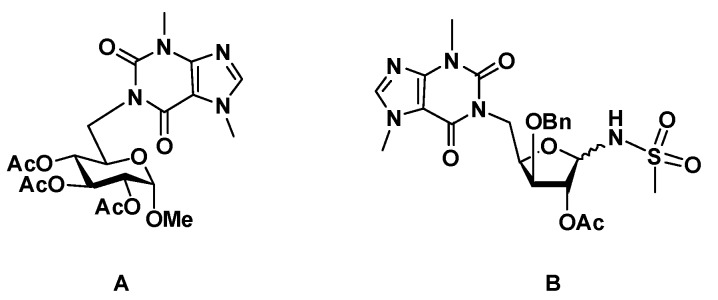
Terminal theobromine isonucleosides, including a glucopyranosid-6′-yl derivative (**A**) and a furanos-5’-yl-based *N*-isonucleosidyl sulfonamide (**B**), as selective AChE inhibitors.

Isonucleosides are regioisomers of nucleosides in which a nucleobase or an analogous nitrogeneous hetereoaromatic motif is linked to the sugar moiety at a non-anomeric position. This group of structures has attracted significant interest in the search of new nucleos(t)ide analogs, owing to the therapeutic potential of these groups of compounds, especially as anticancer and antiviral agents [1,2]. The lack of an *N*-glycosidic bond in isonucleosides linking the sugar and nucleobase moieties confers them a higher chemical and enzymatic stability than that of nucleosides. Most of the reported isonucleosides possess the nucleobase linked to furanose systems at C-2 or at C-3 [3,4,5,6,7,8,9,10,11], among which some molecules exhibited anticancer [4,5] or antiviral [6,7,8,9,11] activities. Pyranosyl isonucleosides have been relatively less exploited and the few reported examples include 2′-fluoroarabino-2′-yl pyrimidine derivatives [12], which displayed anticancer activities, and pyranos-6′-yl isonucleosides [13,14], which resulted from our previous investigations and showed selective and moderate to good inhibition of acetylcholinesterase (AChE). This enzyme hydrolyses the neurotransmitter acetylcholine and is a main therapeutic target for the symptomatic treatment of Alzheimer’s disease (AD) [15,16]. Isonucleosides comprising 2-acetamido-6-chloropurine or theobromine units linked to methyl glucoside moieties and a bis-glucopyranosid-6-yl thymine derivative were the most significant inhibitors with *K*_i_ values ranging from 7.1 µM to 4.3 µM. The good inhibitory effect of the theobromine 6′-isonucleoside (**A**, Figure 1) motivated the synthesis of furanosyl analogs, which included an *N*-isonucleosidyl sulfonamide derivative (**B**, Figure 1) [17]. This isonucleotide analog, in which a sulfonamide is a neutral surrogate and a potential mimetic of a phosphate group, also showed micromolar and selective inhibition of AChE, besides revealing low toxicity in normal fibroblasts and in a neuronal cell, indicating therefore the interest of this type of skeleton in the search for new lead molecules for AD.

Encouraged by our previous results, we further explored the synthesis and biological potential of terminal isonucleosides, focusing on furanosyl derivatives and giving further emphasis to theobromine derivatives. We report herein on the synthesis of various xylofuranos-5′-yl purine and uracil derivatives, furanosyl or pyranosyl xylo/gluco-configured terminal theobromine isonucleosides and theobromine *N*-isonucleosidyl analogs of compound **B** comprising anomeric sulfonamide and phosphoramidate moieties. Molecules possessing a long *O*-alkyl chain at C-3 (octyl, dodecyl groups), instead of an *O*-benzyl group present in the previous reported theobromine furanosyl derivatives (such as **B**), were accessed. The presence of the long hydrocarbon chain may enhance the aptitude of the compounds to cross the blood brain barrier, which is a relevant property to consider when planning anti-AD agents [18], particularly if brain AChE is targeted. The ability of the synthesized compounds to inhibit cholinesterases (ChEs) was further evaluated. Moreover, in view of the anticancer potential of nucleos(t)ide analogs, the cytotoxicity of the new isonucleosides on cancer cells was assessed.

## 2. Results

### 2.1. Chemistry

**Scheme 1:**
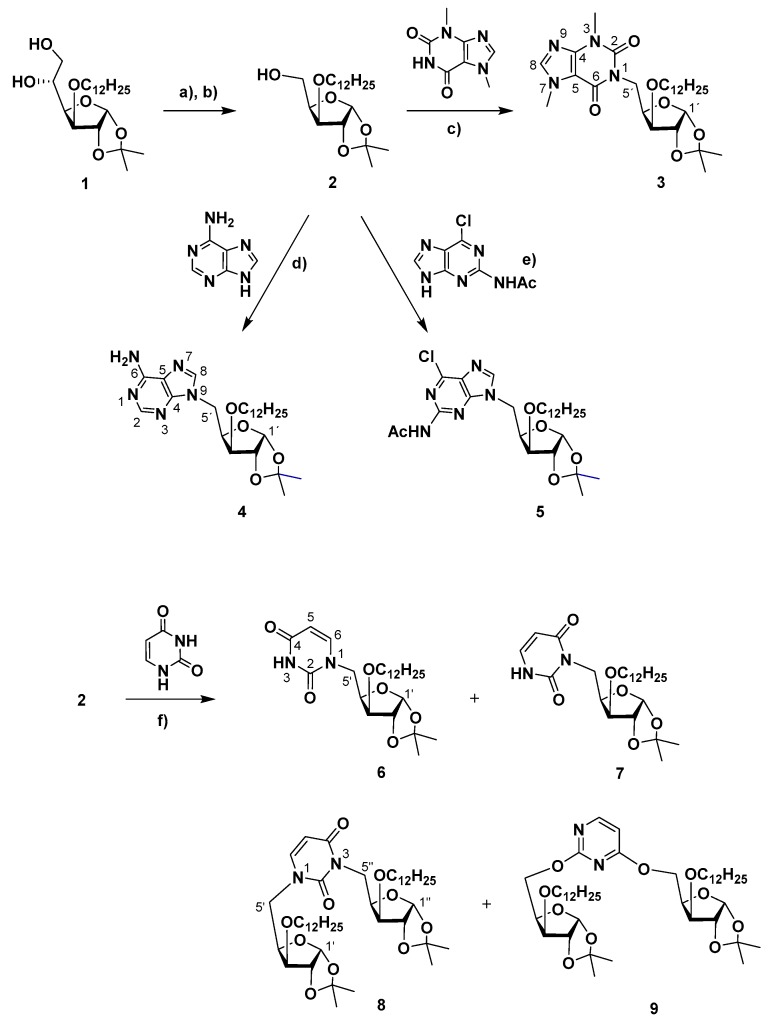
Reagents and conditions: (**a**) NaIO_4_, THF (60 % aq. soln.), room temp., 2.5 h, 79% [19]; (**b**) NaBH_4_, EtOH/H_2_O, room temp., 1 h, 81% [19]; (**c**) theobromine, PPh_3_, diethyl azodicarboxylate (DEAD), THF, 50 °C, 48 h, 16% or THF/DMF, MW, max. 150 W, 65 °C, 30 min, 44%; (**d**) adenine, PPh_3_, DEAD, THF/DMF, MW, max. 150 W, 65 °C, 50 min, 31%; (**e**) 2-NHAc-6-Cl-purine, PPh_3_, DEAD, THF, 50 °C, 16 h, 51%; (**f**) uracil, PPh_3_, DEAD, THF, 50 °C, 18 h, 12% (**6**), 13% (**7**), 29% (**8**), 5% (**9**).

The synthesis of 3′-*O*-dodecyl xylofuranos-5′-yl isonucleosides was based on the Mitsunobu coupling between the 3′-*O*-dodecyl-1,2-*O*-isopropylidene xylofuranose precursor (**2**), which was prepared from 3′-*O*-dodecyl-1,2-*O*-isopropylidene glucofuranose (**1**) [19], with purine derivatives and uracil in the presence of diethyl azodicarboxylate (DEAD) and triphenylphosphine (Scheme 1). The reaction with the purine alkaloid theobromine in THF at 50 °C during 2 days led to the desired isonucleoside **3** in a low yield (16%). The conversion was significantly improved when performing the reaction under microwave irradiation (MW, 150 W, Pmax 250 Psi) at 65 °C in a mixture of tetrahydrofuran/*N*,*N*-dimethylformamide (THF/DMF), conducting to **3** in a satisfactory yield of 44% after only 30 min. The coupling of **2** with adenine was carried out using the above mentioned MW-assisted protocol, since we have previously observed low conversions even after long reaction times (16–96 h) in the Mitsunobu reaction between this nucleobase and partially protected glucosides under conventional heating [14]. The N^9^-xylofuranos-5′-yl adenine **4** was obtained in moderate yield (33%) within 50 min as the sole heterocoupling product, whose N^9^‒C-5 linkage was determined by HMBC correlations between protons H-5′ and C-4/C-8 of the purine moiety. N^9^-regioselectivity also occurred in the coupling between **2** and 2-acetamido-6-chloropurine, which was performed under conventional heating conditions leading to the isonucleoside **5** in 51% yield. Similarly, as for **4**, the HMBC spectra of **5** showed the key correlations between H-5′ and both C-4/C-8 which comproved the regiochemistry of the isonucleosidic bond. In contrast with purines, the Mitsunobu reaction involving uracil afforded different regiosomeric products of both mono- and bis-coupling, an outcome previously found when using pyrimidines in analogous reactions performed under conventional heating [14]. The isonucleosides comprising the uracil moiety linked by N^1^ (**6**) and by N^3^ (**7**) were obtained in identical yields (12%–13%) and were assigned based on the HMBC correlations of the protons H-5′ with C-2/C-6 or with C-2/C-4, respectively. The distinct chemical shifts for C-5′ in the ^13^C NMR spectra of **6** and **7**, at 48.1 ppm and at 40.1 ppm, respectively, were additional indicative spectral features for their regiochemical elucidation. The uracil-linked disaccharides N^1^,N^3^- and 2-*O*,4-*O*-xylofuran-5-yl uracils (**8**, **9**) were formed in yields of 29% and 5%, respectively. Assignment of the N^1^,N^3^-bis-xylofuranos-5-yl uracil (**8**) was based on its NMR spectral data, which combined the features of both N^1^- and N^3^-linked isonucleosides, namely through the HMBC correlations between H-5′ protons with C-2/C-6 and between H-5″ with C-2/C-4 as well as the C-5′ and C-5″ resonances at 49.0 ppm and 40.8 ppm, respectively. In the case of the 2,4*-*bis*-O*-xylofuranos-5-yl uracil derivative (**9**), the ^1^H NMR and ^13^C NMR signals of the uracil moiety are deshielded relatively to those of the N^1^,N^3^-linked regioisomer **8**. The differences between the signals of H-6 and H-5 of the uracil ring are particularly notorious, appearing at 8.18 ppm and 6.41 ppm in **9**, whereas in the case of **8** these protons resonate at 7.24 ppm and 5.69 ppm, respectively. In addition, the signals of C-5′ and C-5″ of the sugar moiety at 64.4 and 64.7 ppm further confirmed their connection to the 2-O and 4-O atoms of the uracil unit.

**Scheme 2:**
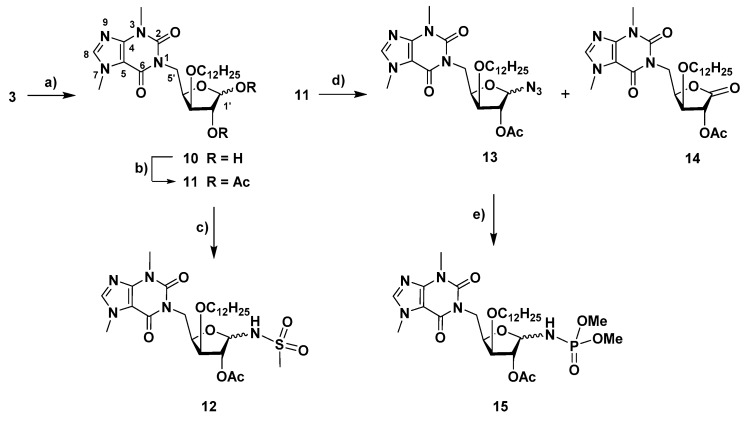
Reagents and conditions: (**a**) trifluoroacetic acid (TFA) (60% aq. soln.), room temp., 2 h, 83% (α/β = 1:0.9; (**b**) Ac_2_O, py, room temp., 1.5 h, 69% (α/β = 1:0.5); (**c**) CH_3_SO_2_NH_2_, BF_3_·Et_2_O, CH_2_Cl_2_/CH_3_CN, room temp., 2 h, 81% (β/α = 1:0.4); (**d**) trimethylsilyl azide (TMSN_3_), trimethylsilyl triflate (TMSOTf), CH_3_CN, 65 °C, MW, max. 150 W, 1 h 40 min, 86% (**13**, β/α ratio, 1:0.8) and 14% (**14**); (**e**) P(OMe)_3_, CH_2_Cl_2_, room temp., 23 h, 56% (α/β = 1:0.3).

Motivated by our previous studies, which revealed the interest of theobromine isonucleosides as promising lead molecules for AD (Figure 1) [14,17], we further focused our synthetic work on a variety of theobromine derivatives. The theobromine xylofuranosyl isonucleoside **3** was the precursor for the synthesis of isonucleotide analogs via derivatization at the anomeric center (Scheme 2). Thus, **3** was subjected to acid-mediated hydrolysis of the acetonide moiety (aq. trifluoroacetic acid (TFA) 60%), leading to diol **10**. Further acetylation (Ac_2_O/pyridine) afforded the isonucleosidyl acetate **11**, which was subsequently converted into *N*-isonucleosidyl derivatives containing polar anomeric moieties capable of hydrogen bond interactions as potential isosteres of a phosphate group. *N*-isonucleosidation of methanesulfonamide with **11** in the presence of BF_3_·Et_2_O provided the *N*-isonucleosidyl sulfonamide **12** in 81% yield, as a 3-*O*-dodecyl analog of isonucleoside **B** (Figure 1). An anomeric mixture (β/α ratio, 1:0.4) was obtained, which may be a result from the α and β-directing effects exerted by the neighboring acetate group participation and by the remote participation of theobromine carbonyl group, respectively, as well as from anomerization via the acyclic *N*-sulfonyl imine intermediate, as previously described in the synthesis of **B** (Figure 1) [17]. On the other hand, the isonucleosidyl acetate **11** was subjected to MW-assisted anomeric azidation with trimethylsilyl azide (TMSN_3_), mediated by trimethylsilyl triflate (TMSOTf), leading to the α- and β-isonucleosidyl azides **13** in a 1:0.8 ratio and 86% yield. The formation of the α-anomer is likely to arise also from the above mentioned remote group participation in assisting the isonucleosidyl cation intermediate, an effect already described to influence the stereochemical outcome of the anomeric azidation of glucuronamide derivatives [17,20]. Moreover, the xylonolactone-containing theobromine isonucleoside **14** was formed as a secondary product in 14% yield and was obtained in a mixture containing the β-isonucleosyl azide, the separation of which could not be achieved by column chromatography. The structure of lactone **14** was identified based on its NMR and high resolution mass spectrometry (HRMS) data. HMBC experiments showed correlations between H-2′, which appeared at rather low field (*δ* = 5.59 ppm), H-3′ and H-4′ with C-1′ (*δ* = 170 ppm). The mechanism leading to the lactone **14** probably involves the formation of an isonucleosidyl imine intermediate, through nitrogen elimination from the anomeric azide moiety, and subsequent hydrolysis during the workup. The azide **13**-**α** was engaged in a Staudinger-type reaction with trimethylphosphite to furnish the *N*-isonucleosidyl phosphoramidate **15** in 56% yield as a 1:0.3 mixture of α- and β-anomers. Despite the α-anomeric configuration of the isonucleosidyl azide precursor, the β-configured product likely arises from anomerization via acyclic *N*-phosphoryl imine/iminium intermediates.

**Scheme 3:**
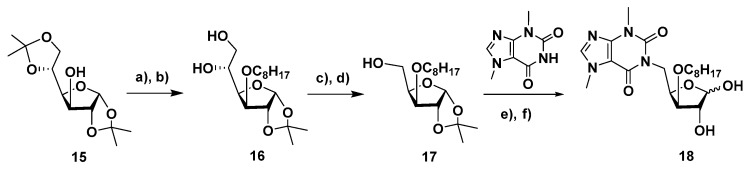
Reagents and conditions: (**a**) C_8_H_17_Br, NaH, DMF, room temp., 22 h; (**b**) AcOH (70% aq. soln.), room temp., 26 h, 88%; 2 steps; (**c**) NaIO_4_, THF (60% aq. soln.), room temp., 4 h; (**d**) NaBH_4_, EtOH/H_2_O, room temp., 1.5 h, 65%, 2 steps; (**e**) theobromine, PPh_3_, DEAD, DMF, 65 °C, MW, max. 150 W, 30 min; (**f**) TFA (60% aq. soln.), room temp., 4 h, 20%, 2 steps.

Furanosyl and pyranosyl theobromine isonucleosides containing a 3-*O*-octyl moiety were also synthesized. The 3-*O*-octyl-1,2-*O*-isopropylidene glucofuranose precursor (**16**, Scheme 3) was accessed through 3-*O*-octylation of diacetone-D-glucose with octyl bromide in the presence of sodium hydride and subsequent selective hydrolysis of the primary acetonide (aq. acetic acid 70%). The diol **16** was then subjected to oxidative cleavage with sodium periodate and the resulting aldehyde was reduced (NaBH_4_) to afford the xylofuranose derivative **17**. The reaction of **17** with theobromine under the MW-assisted Mitsunobu conditions, which was followed by removal of the isopropylidene moiety, gave the xylofuranos-5′-yl theobromine **18**, as an anomeric mixture, in 20% overall yield.

**Scheme 4:**
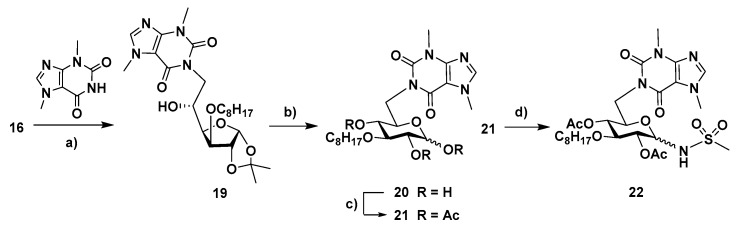
Reagents and conditions: (**a**) theobromine, PPh_3_, DEAD, DMF, reflux, 20 h, 16%; (**b**) TFA (60% aq. soln.), room temp., 5 h, 87% (α /β = 1:0.9); (**c**) Ac_2_O, py, room temp., 2.5 h, 84% (α /β = 1:0.7); (**d**) CH_3_SO_2_NH_2_, BF_3_·Et_2_O, CH_2_Cl_2_/CH_3_CN, room temp., 3.5 h, 38% (β/α = 1:0.4).

The direct regioselective Mitsunobu coupling of diol **16** with theobromine at C-6 could also be achieved, although in low yield (16%), when carrying out the reaction in refluxing DMF (Scheme 4), while the previously mentioned MW-assisted protocol did not enable significant conversion of **16**. Acid hydrolysis of the obtained glucofuranos-6′-yl theobromine **19** led to the pyranos-6′-yl isonucleoside **20**, the acetylation of which provided the tri-*O*-acetylated derivative **21**. The regiochemistry of the isonucleosidic linkage (N^1^-C-6) in **20** was confirmed by the HMBC correlations between the protons H-6′ and both C-2/C-6. The BF_3_Et_2_O-mediated reaction of **21** with methanesulfonamide gave the *N*-isonucleosidyl sulfonamide **22** in 38% yield as an anomeric mixture (β/α ratio, 1:0.4).

### 2.2. Biological Evaluation

The newly synthesized xylofuranosyl isonucleosides (**3**–**10** and **18**), the theobromine-containing isonucleosidyl sulfonamides (**12** and **22**), azide (**13-α**) and phosphoramidate (**15**) and the glucopyranos-6′-yl theobromine derivative **21** were subjected to biological evaluation. Their ability to inhibit the enzymes acetylcholinesterase (AChE, from *Electrophorus electricus*) and butyrylcholinesterase (BChE, from equine serum) was assessed by the Ellman’s method. The cholinesterase inhibitor galantamine hydrobromide, which is clinically used for the treatment of AD, was used as a standard. The percent inhibition (at 50 µM) determined for the compounds, the inhibition constants, *K*_i_ (for competitive inhibition) or *K*_i_′ (for uncompetitive inhibition) for the active molecules, and the respective types of inhibition are compiled in Table 1.

**Figure 2:**
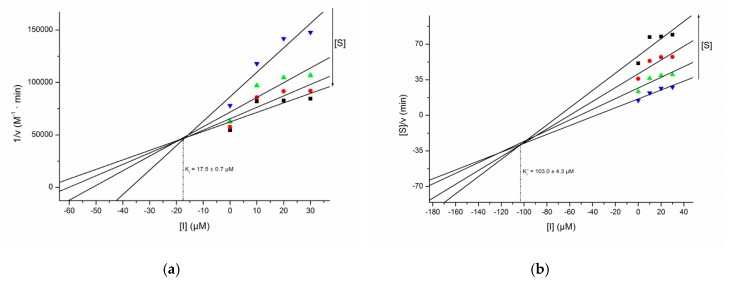
Dixon (**a**) and Cornish–Bowden (**b**) plots for the inhibition of AChE by compound **9**.

Among the furanosyl isonucleosides, only the 2-*O*,4-*O*-uracil-linked pseudodisaccharide **9** showed significant effect on the activity of cholinesterases, exhibiting a moderate mixed-type inhibition of AChE, in which the competitive character is more pronounced than the uncompetitive one (*K*_i_ = 17.5 µM, *K*_i_′ = 103.0 µM, Figure 2). The remaining furanosyl isonucleosides were considered inactive, showing, at 50 µM concentration, less than 40% inhibition of the activity of both enzymes. The theobromine-containing furanosyl isonucleotide analogs (**12**, **15**), containing sulfonamide or phosphoramidate groups, as well the isonucleosidyl azide **13**-**α**, were also devoid of any inhibitory effects. Given the fact that the previously reported *N*-isonucleosidyl sulfonamide **B** (Figure 1) showed effective inhibition of AChE, the lack of activity of the 3-*O*-dodecylated analog **12** clearly demonstrates that the presence of the 3-*O*-benzyl group in **B** is crucial for its inhibitory activity. In contrast, replacing a 3-*O*-dodecyl by a 3-*O*-octyl group appears not to cause a significant change on the activity of xylofuranos-5′-yl theobromine derivatives, as demonstrated by the similar effects of isonucleosides **10** and **18**.

**Figure 3:**
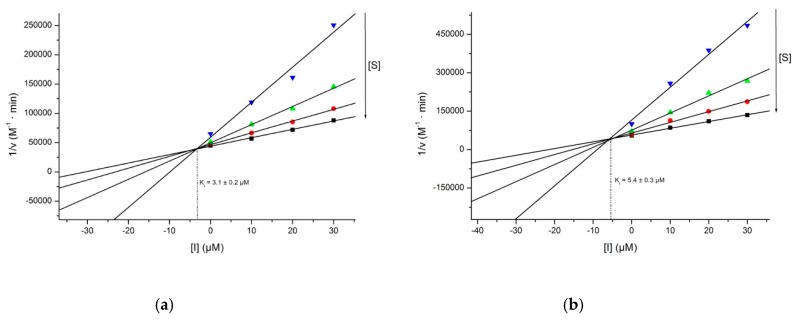
Dixon plots for the inhibition of AChE (**a**) and BChE (**b**) by compound **21**.

With respect to glucopyranos-6′-yl theobromine isonucleosides, the isonucleosidyl acetate **21** displayed noteworthy inhibitory effects on both cholinesterases. It was the most active compound of the series, exhibiting dual inhibition of AChE and BChE by a mixed-type mechanism with a dominant competitive component, with single digit micromolar *K*_i_ values (Figure 3) that are at least ca. 32-fold (*K*_i_ = 3.1 µM) and 11-fold lower (*K*_i_ = 5.4 µM) than their *K*_i_′ values, respectively. It was only twice less active than the standard galantamine hydrobromide on BChE. The inhibition of BChE may provide therapeutic benefits in AD, since an increasing activity of this enzyme with accompanying decreasing levels of its congener AChE occurs over the course of the disease. Thus, the brain acetylcholine levels became gradually dependent on BChE [21] and a dual AChE/BChE inhibition or a selective BChE inhibition may provide a more effective treatment in advanced stages of AD. The *N*-isonucleosidyl sulfonamide **22** was a weaker cholinesterase inhibitor than its precursor **21**, displaying selective effect towards BChE with 53% inhibition of the enzymatic activity at 50 µM.

The compounds were tested for their cytotoxicity against human cancer cell lines, namely A375 (epithelial melanoma), A2780 (ovarian carcinoma), HT29 (colorectal adenocarcinoma), MCF7 (breast adenocarcinoma) and SW1736 (thyroid carcinoma), as well as towards non-malignant mouse embryonic fibroblasts (NIH 3T3), using the sulforhodamine-B (SRB) colorimetric assay. None of the molecules revealed cytotoxic effects at concentrations below 30 µM (cut-off of the assay) to either cancer or healthy cells, being therefore considered inactive (EC_50_ > 30 μM) against the tested cell lines.

### 2.3. Molecular Docking Studies

To further understand the binding interactions on a molecular level of compounds **9** and **21**, a molecular docking was performed with Autodock 4 [22]. The crystal structures of AChE (PDB: 1C2O) and BChE (PDB: 6EMI) were retrieved from the RCSB Protein Databank. Due to its anomeric mixture, compound **21** was docked as α and β anomers.

**Figure 4:**
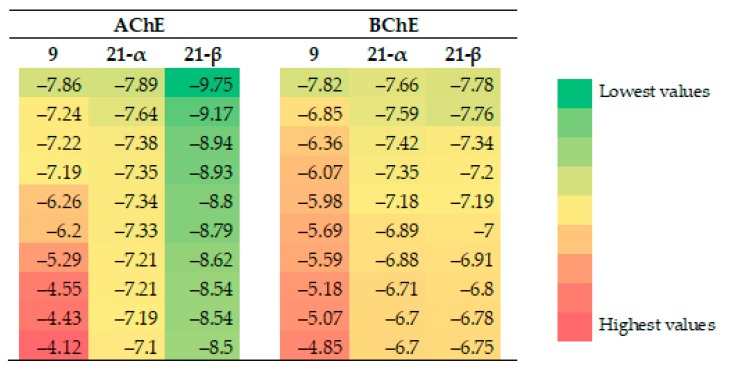
Binding energies (kcal/mol) of the top docking poses of compounds **9**, **21-α** and **21-β** into AChE and BChE.

**Figure 5:**
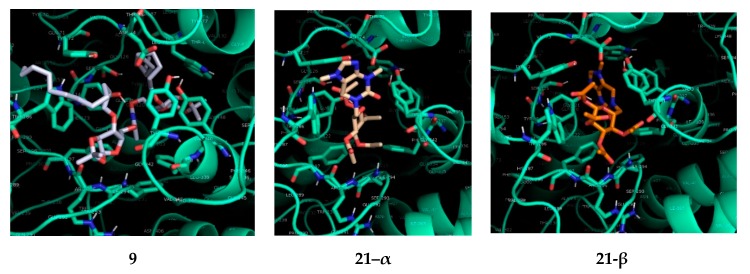
Lowest-energy binding poses of compounds **9**, **21-α** and **21-β** to AChE.

Concerning the AChE docking results, **21**-**β** outperformed compounds **21**-**α** and **9** significantly (Figure 4). With respect to the docking positions of **21**-**β**, this compound seems to fit quite nicely into the entrance of the cavity, where the theobromine moiety is positioned at the entrance of the active site gorge, pointing towards the enzyme’s binding pocket (Figure 5). The main interaction of the theobromine unit is with Tyr124 at the peripheral anionic site (PAS), which appears to be based on an O–H∙∙∙N hydrogen bond with one of the theobromine nitrogen atoms. The tri*-O-*acetylated glucopyranosyl unit is located in front of the binding pocket, where the acetyl groups bind via N–H∙∙∙O hydrogen bonds to Trp286 at the PAS and Arg296, located between the acyl pocket and the PAS, or Phe295 at the acyl binding pocket, where the N–H∙∙∙O binding cannot be differentiated between these residues.

In compound **21**-**α**, the conformation however hinders the theobromine moiety to enter the active site and give a favorable binding position (Figure 5). Therefore, the theobromine system is mainly located outside of the active site of AChE, establishing as key interaction an O–H∙∙∙Ar π-interaction with Tyr72 at the PAS. Another strong binding occurs with one of the acetyl groups with Phe294.

The docking of compound **9** (Figure 5) shows a strong O–H∙∙∙N interaction of Tyr124 at the PAS with one of the pyrimidines nitrogens. Herein it seems that compound **9** is able to enter the binding pocket where one half fills the cavity of the AChE active site while the other furanosyl moiety sticks out and blocks the entrance of the binding pocket, where Phe295 shows an O–H∙∙∙O binding to the furanosyl oxygen.

The docking of **9**, **21**-**α** and **21**-**β** in the structure of BChE resulted in quite similar results, whereas the binding interactions of compound **9** in comparison to **21** fall off significantly (see Figure S20, in the Supplementary Materials). The best docking position however shows a strong binding from π-stacking of the pyrimidine moiety of **9** with Tyr332. This also is a major binding mode of **21**-**β** as this compound also shows a strong π-stacking interaction of the theobromine moiety with Tyr332 and weak interactions of the acetyl groups with sidechains present in the active site of BChE. Compound **21**-**α** shows an interaction of the theobromine nitrogens with the peptide carbonyl of Leu286 and Ser287. However, for the BChE active site, it is difficult to identify strong interactions between the inhibitors and its amino acid residues.

## 3. Discussion

A variety of novel terminal isonucleosides embodying purine derivatives or uracil were synthesized using the Mitsunobu coupling as a key step. Microwave irradiation proved to be a useful tool in the Mitsunobu reaction between 3-*O*-dodecyl/octyl-1,2-*O*-isopropylidene xylofuranose with theobromine or adenine, enabling the access to 5′-isonucleosides in shorter reaction times (30‒50 min) than those needed in conventional stirring for analogous reactions (>16 h) and in moderate yields. In the case of the synthesis of the 3′-*O*-dodecyl-xylofuranos-5′-yl theobromine derivative **3**, a significant increase on the reaction yield (from 16% to 44%) was achieved using MW-assisted heating. Potential nucleotide mimetics, namely theobromine-based *N*-isonucleosidyl sulfonamides or an *N*-isonucleosidyl phosphoramidate, which is a previously unreported type of structure, were synthesized through anomeric functionalization of isonucleosidyl acetates, whose stereochemical outcome was influenced by the remote participating effect of the theobromine carbonyl groups.

Biological evaluation revealed the ability of a theobromine isonucleosidyl acetate possessing a glucopyranose moiety (**21**) to inhibit both AChE and BChE at single-digit micromolar concentrations, reinforcing the potential of theobromine isonucleosides as cholinesterase inhibitors. Since the anomeric mixture was evaluated, it appears that **21**-**β** accounts more for the detected competitive inhibitory effect on AChE, as indicated by the docking simulations. The theobromine unit in **21**-**β** as well as the sugar moiety are important for the binding to the enzyme, establishing interactions at the PAS and at the front of the active site, respectively.

The *N*-isonucleosidyl sulfonamide derivative (**22**) showed significantly weaker effects than its precursor **21**, a result which is in contrast with our previous findings that suggested that an anomerically linked sulfonamide moiety, namely in compound **B** (Figure 1), would lead to an increase on the AChE inhibitory ability of the theobromine isonucleoside. This result as well as the lack of activity of the 3-*O*-dodecyl theobromine *N*-isonucleosidyl sulfonamide **12** indicate that the 3-O-substituent and the sugar ring system in these types of molecules also account for their ChE inhibitory abilities. A uracil-linked pseudodisaccharide (**9**) was the only xylofuranose-based isonucleoside which showed significant ChE inhibitory effect, with selective and moderate activity on AChE, which further demonstrate the AChE inhibitory potential of molecules based on this type of skeleton, notwithstanding its large size. Similarly, to a thymine-linked pseudodisaccharide previously described as a competitive AChE inhibitor [14], the interactions between compound **9** and the enzyme seem also to span both the PAS and the active site, with half of the molecule blocking the entrance to the active site gorge and the pyrimidine unit being involved in a key interaction.

The lack of cytotoxicity of the tested molecules to either cancer or non-malignant cells reflects the lower propensity of terminal nucleosides than non-terminal ones to exhibit cytotoxic effects. It is known that 2′-isonucleosides can be, although in a low extend, intracellularly phosphorylated at the primary hydroxyl group by kinases [9,23] and that isonucleoside triphosphates can be recognized by DNA polymerases and be incorporated into the growing DNA chain arresting its elongation [24], similarly to the mechanism of action of the known anticancer nucleosides [1,2]. The absence of a terminal hydroxyl group able for phosphorylation in these newly synthesized 5′-/6′-isonucleosides precludes them to undergo such biological pathway which eventually would lead to cytotoxic effects.

Nevertheless, the non-toxicity of these molecules to healthy cells motivates studies focusing on other biological properties to further explore their therapeutic interest.

## 4. Materials and Methods 

### 4.1. Chemistry

#### 4.1.1. General Methods

The progress of the reactions was checked by thin layer chromatography (TLC) using silica gel aluminum plates (Merck 60 F_254_) with visualization under UV light (254 nm) and/or by immersion in a 10% H_2_SO_4_ solution in ethanol or in a solution of cerium (IV) sulfate (0.2% *w/v*) and ammonium molybdate (5% *w/v*) in H_2_SO_4_ (6% aq.) followed by heating (200 °C). Microwave experiments were carried out in a CEM Discover SP Microwave Synthesizer. Flash column chromatography was performed on silica gel 60 G (0.040–0.063 mm, E. Merck). Optical rotations (589 nm, 20 °C) were measured on a Perkin–Elmer 343 polarimeter. NMR spectra were acquired using a BRUKER Avance 400 spectrometer operating at 400.13 MHz for ^1^H or 100.62 MHz for ^13^C. The chemical shifts are given in parts per million (ppm). The spectra were calibrated with internal TMS (in the case of ^1^H NMR spectra in CDCl_3_) or with the respective solvent residual peak. Coupling constants (*J*) are given in hertz (Hz). Assignments were made with the help of 2D experiments (COSY, HSQC, HMBC). HRMS spectra were acquired with a QqTOF Impact II mass spectrometer (Bruker Daltonics) equipped with an electrospray ion source (ESI) and were recorded in positive mode.

Synthesis of compound **2** was previously described [19].

#### 4.1.2. General Procedure for the Mitsunobu Coupling of 3-*O*-Octyl/Dodecyl-1,2-*O*-isopropylidene-α-D-xylo/glucofuranose (**2**, **17**) with Purine Derivatives/Uracil

To a solution of partially protected 1,2-*O*-isopropylidene-α-D-xylo/glucofuranose (1 mmol) in THF (28 mL) or DMF (10 mL) under nitrogen atmosphere, PPh_3_ (2 equiv.), diethyl azodicarboxylate (DEAD, 2 equiv.) and purine derivative/uracil (2 equiv.) were added. The mixture was stirred under the conditions indicated further. The mixture was concentrated under vacuum and the crude residue was subjected to column chromatography.

#### 4.1.3. General Microwave-Assisted Procedure for the Mitsunobu Coupling of 3-*O*-Octyl/Dodecyl-1,2-*O*-isopropylidene-α-D-xylofuranose (**2**, **17**) with Theobromine/Adenine

To a solution of partially protected 1,2-*O*-isopropylidene-α-D-xylofuranose (0.2 mmol) in THF/DMF (1:1, 2 mL) or DMF (2 mL), PPh_3_ (2 equiv.), diethyl azodicarboxylate (DEAD, 2 equiv.) and theobromine (2 equiv.) were sequentially added. The mixture was stirred under microwave irradiation (150 W max., P max = 250 Psi) at 65 °C for 30–50 min. The solvent was evaporated, and the crude residue was subjected to column chromatography on silica-gel.

#### 4.1.4. 1-(5-Deoxy-3-*O*-dodecyl-1,2-*O*-isopropylidene-α-D-xylofuranos-5-yl)-3,7-dimethyl-3,7-dihydro-1*H*-purine-2,6-dione (**3**)

Obtained according to the general procedure for Mitsunobu coupling, starting from compound **2** (328 mg, 0.916 mmol), theobromine (340 mg, 1.89 mmol) and using PPh_3_ (502 mg, 1.91 mmol) and DEAD (40% wt. soln. in toluene, 0.89 mL, 1.95 mmol) in THF (25 mL). The reaction mixture was stirred at 50 °C for 48 h. Purification by column chromatography (from AcOEt/hexane 1:1 to 4:1) afforded **5** (76 mg, 16%) as a yellow oil.

Alternatively, the title compound could be obtained using the MW-assisted procedure, starting from compound **2** (79 mg, 0.219 mmol), theobromine (80 mg, 0.44 mmol) and using PPh_3_ (115 mg, 0.43 mmol) and DEAD (40% wt. soln. in toluene, 0.2 mL, 0.44 mmol) in THF/DMF (1:1, 2 mL), within a reaction mixture of 30 min and in 44% yield (50 mg). ${\left[\alpha \right]}_{D}^{20}$ = −5 (c = 1, in CH_2_Cl_2_). ^1^H NMR (CDCl_3_, 400 MHz): *δ* 7.49 (s, 1H, H-8), 5.97 (d, 1 H, H-1′, *J*_1′,2′_ = 3.9), 4.74 (dd, 1 H, H-5′a, *J*_4′,5′a_ = 8.8*, J*_5′a,5′b_ = 14.1), 4.59–4.52 (m, 2 H, H-2’, H-4′), 3.99–3.91 (m, 4 H, C*H*_3_, N^7^, H-5′b, *J*_4′,5′b_ = 1.8), 3.90 (d, 1 H, H-3′, *J*_3′,4′_ = 3.4), 3.69–3.59 (m, 1 H, H-1″a), 3.56 (s, 3 H, C*H*_3_-N^3^)_,_ 3.48–3.38 (ddd, H-1″b), 1.62–1.51 (m 2 H, C*H*_2_-2″), 1.43 (s, 3 H, C*H*_3_, *i*-Pr), 1.38–1.16 (m, 21 H, C*H*_2_-3″ to C*H*_2_-11″, C*H*_3_, *i*-Pr), 0.86 (t, 3 H, C*H*_3_-12″, *J* = 6.7). ^13^C NMR (CDCl_3_, 100 MHz): δ 155.4 (C-6), 151.7 (C-2), 148.9 (C-4), 141.5 (C-8), 111.5 (Cq, *i*-Pr), 107.9 (C-5), 105.3 (C-1′), 83.5 (C-3′), 82.5 (C-2′), 78.6 (C-4′), 70.5 (*C*H_2_-1″), 40.0 (C-5′), 33.7 (*C*H_3_, N^7^), 32.1 (*C*H_2_-2″), 29.8, 29.8, 29.8, 29.8, 29.7, 29.6, 29.5, 26.8 (*C*H_3_, N^3^, C*H*_2_-3″ to C*H*_2_-10″), 26.3, 26.3 (2 × *C*H_3_, *i*-Pr), 22.8 (*C*H_2_-11″), 14.3 (*C*H_3_-12″). HRMS: calcd for C_27_H_44_N_4_O_6_ [M + H]^+^ 521.3334, found 521.3333; calcd for C_27_H_44_N_4_O_6_ [M + Na]^+^ 543.3153, found 543.3149.

#### 4.1.5. 9-(5-Deoxy-3-*O*-dodecyl-1,2-*O*-isopropylidene-α-D-xylofuranos-5-yl)adenine (**4**)

Obtained according to the general MW-assisted procedure for Mitsunobu coupling, starting from compound **2** (80 mg, 0.22 mmol), adenine (60 mg, 0.44 mmol) and using PPh_3_ (117 mg, 0.44 mmol) and DEAD (40% wt. soln. in toluene, 0.2 mL, 0.44 mmol), in THF/DMF (1:1, 2 mL). The reaction mixture was stirred for 50 min. Purification by column chromatography (from AcOEt/cyclohexane 2:1 to AcOEt) afforded **4** (33 mg, 31%) as a colorless oil. ^1^H NMR (CDCl_3_, 400 MHz): *δ* 8.36 (s, 1 H, H-2), 7.93 (s, 1 H, H-8), 5.96 (d, 1 H, H-1′, *J*_1’,2’_ = 3.6), 5.69 (br.s, 2 H, N*H*_2_), 4.52 (d, 1 H, H-1′, *J*_1’,2’_ = 3.2), 4.64–4.50 (m, 3 H, H-2′, H-5′a, H-4′), 4.35 (dd, 1 H, H-5′b, *J*_5’a,5’b_ = 15.9, *J*_4’,5’b_ = 10.0), 3.87 (d, 1 H, H-3′, *J*_2′,3′_ = 2.8 ), 3.69–3.60 (m, 1 H, H-1″a), 3.47–3.38 (m, 1 H, H-1″b), 1.62–1.53 (m 2 H, C*H*_2_-2″), 1.42–1.20 (m, 26 H, C*H*_2_-3″ to C*H*_2_-11″, 2 × C*H*_3_, *i*-Pr), 0.87 (t, 4.2 H, C*H*_3_-12″, *J* = 6.7). ^13^C NMR (CDCl_3_, 100MHz): δ 155.4 (C-6), 153.0 (C-2), 150.3 (C-4), 141.7 (C-8), 119.6 (C-5), 112.0 (Cq, *i*-Pr), 105.3 (C-1′), 82.7 (C-3′), 82.2 (C-2′), 78.4 (C-4′), 70.6 (*C*H_2_-1″), 42.9 (C-5′), 32.1 (*C*H_2_-2″), 29.8, 29.8, 29.8, 29.8, 29.7, 29.6, 29.5, 26.9, 26.3, 26.3, 22.8 (C*H*_2_-3″ to C*H*_2_-11″, 2 × *CH*_3_, *i*-Pr), 14.3 (*C*H_2_-12″). HRMS: calcd for C_25_H_41_N_5_O_4_ [M + H]^+^ 476.3231, found 476.3226. calcd for C_25_H_41_N_5_O_4_ [M + Na]^+^ 498.3051, found 498.3043. 

#### 4.1.6. 2-Acetamido-6-chloro-9-(5-deoxy-3-*O*-dodecyl-1,2-*O*-isopropylidene-α-D-xylofuranos-5-yl)purine (**5**)

Obtained according to the general procedure for Mitsunobu coupling, starting from compound **2** (337 mg, 0.94 mmol), 2-acetamido-6-chloropurine (399 mg, 1.88 mmol) and using PPh_3_ (504 mg, 1.92 mmol) and DEAD (40% wt. soln. in toluene, 0.82 mL, 1.8 mmol) in THF (25 mL). The reaction mixture was stirred at 50 °C for 16 h. Purification by column chromatography (from AcOEt/petroleum ether 1:2) afforded **5** (265 mg, 51%) as a yellow oil.

${\left[\alpha \right]}_{D}^{20}$ = −14 (c = 1, in CH_2_Cl_2_). ^1^H NMR (400 MHz, CDCl_3_) δ 8.11 (s, 1 H, H-8), 5.95 (d, 1 H, H-1’, *J*_1′,2′_ = 3.7), 4.60 (d, 1 H, H-2′), 4.56–4.46 (m, 2 H, H-4′, H-5′a), 4.39 (dd, 1 H, H-5′b, *J*_4′,5′b_ = 9.0*, J*_5′a,5′b_ = 15.0), 3.85 (d, 1 H, H-3′, *J*_3′,4′_ = 3.0), 3.68–3.58 (m, 1 H, H-1″a), 3.45–3.35 (ddd, H-1″b), 2.54 (s, 3 H, C*H*_3_, NHAc), 1.60–1.49 (m 2 H, C*H*_2_-2″), 1.40 (s, 3 H, C*H*_3_, *i*-Pr), 1.36–1.16 (m, 21 H, C*H*_2_-3″ to C*H*_2_-11″, C*H*_3_, *i*-Pr), 0.87 (t, 3 H, C*H*_3_-12″, *J* = 6.7). ^13^C NMR (100 MHz, CDCl_3_) *δ*: 153.0 (C-4), 152.0, 151.3 (C-2, C-6), 145.7 (C-8), 128.0 (C-5), 112.2 (Cq, *i*-Pr), 105.3 (C-1′), 82.6 (C-3′), 82.0 (C-2′), 77.8 (C-4′), 70.6 (*C*H_2_-1″), 43.2 (C-5′), 32.0 (*C*H_2_-2″), 29.8, 29.8, 29.8, 29.8, 29.7, 29.6, 29.5, 26.9 (C*H*_2_-3″ to C*H*_2_-10″), 26.3, 26.2 (2 × *C*H_3_, *i*-Pr), 25.3 (*C*H_3_, NHAc), 22.8 (*C*H_2_-11″), 14.3 (*C*H_3_-12″). HRMS: calcd for C_27_H_42_ClN_5_O_5_ [M + H]^+^ 552.2947, found 552.2957; calcd for C_27_H_42_ClN_5_O_5_ [M + Na]^+^ 574.2767, found 574.2778.

#### 4.1.7. 1-(5-Deoxy-3-*O*-dodecyl-1,2-*O*-isopropylidene-α-D-xylofuranos-5-yl)uracil (**6**), 3-(5-deoxy-3-*O*-dodecyl-1,2-*O*-isopropylidene-α-D-xylofuranos-5-yl)uracil (**7**), 1,3-bis-(5-deoxy-3-*O*-dodecyl-1,2-*O*-isopropylidene-α-D-xylofuranos-5-yl)uracil (**8**) and 2,4-bis-*O*-(5-deoxy-3-*O*-dodecyl-1,2-*O*-isopropylidene-α-D-xylofuranos-5-yl)uracil (**9**)

Obtained according to the general procedure for Mitsunobu coupling, starting from compound **2** (286 mg, 0.798 mmol), uracil (182 mg, 1.62 mmol) and using PPh_3_ (425 mg, 1.62 mmol) and DEAD (40% wt. soln. in toluene, 0.7 mL, 1.54 mmol) in THF (25 mL). The reaction mixture was stirred at 50 °C for 18 h. Purification by column chromatography (from AcOEt/petroleum ether 1:5 to AcOEt) afforded **9** (17 mg, 5%), **8** (91 mg, 29%), **6** (42 mg, 12%) and **7** (46 mg, 13%) as colorless oils.

Compound **6**: ${\left[\alpha \right]}_{D}^{20}$ = −2 (c = 1, in CH_2_Cl_2_). ^1^H NMR (CDCl_3_, 400 MHz): *δ* 8.99 (s, 1 H, NH), 7.30 (d, 1 H, H-6, *J*_5,6_ = 7.9), 5.91 (d, 1 H, H-1’, *J*_1’,2’_ = 3.7), 5.66 (d, 1 H, H-5), 4.56 (d, 1 H, H-2’), 4.41 (dt, 1 H, H-4′, *J*_4′,5′b_ = 8.8), 4.33 (dd, 1 H, H-5′a, *J*_4′,5′a_ = 2.6*, J*_5′a,5′b_ = 14.6), 3.85 (d, 1 H, H-3′, *J*_3′,′_ = 3.2), 3.69–3.57 (m, 2 H, H-1″a, H-5′b), 3.44–3.34 (ddd, H-1″b), 1.60–1.50 (m 2 H, C*H*_2_-2″), 1.46 (s, 3 H, C*H*_3_, *i*-Pr), 1.34–1.18 (m, 21 H, C*H*_2_-3″ to C*H*_2_-11″, C*H*_3_, *i*-Pr), 0.87 (t, 3 H, C*H*_3_-12″, *J* = 6.7). ^13^C NMR (CDCl_3_, 100MHz): δ 163.8 (C-4), 151.1 (C-2), 145.7 (C-6), 112.1 (Cq, *i*-Pr), 105.2 (C-1′), 102.1 (C-5), 82.9 (C-3′), 82.2 (C-2′), 78.1 (C-4′), 70.6 (*C*H_2_-1″), 48.1 (C-5′), 32.0 (*C*H_2_-2″), 29.8, 29.8, 29.8, 29.7, 29.7, 29.5, 29.5, 26.8 (C*H*_2_-3″ to C*H*_2_-10″) 26.3, 26.2 (2 × *C*H_3_, *i*-Pr), 22.8 (*C*H_2_-11″), 14.3 (*C*H_3_-12″). HRMS: calcd for C_24_H_40_N_2_O_6_ [M + Na]^+^ 475.2779, found 475.2782; calcd for C_24_H_40_N_2_O_6_ [M + H]^+^ 453.2959, found 453.2962.

Compound **7**: ${\left[\alpha \right]}_{D}^{20}$ = +6 (c = 1, in CH_2_Cl_2_).^1^H NMR (CDCl_3_, 400 MHz): *δ* 9.98 (br.d, 1 H, N*H*), 7.20 (dd, 1 H, H-6, *J*_NH,6_ = 5.8, *J*_5,6_ = 7.6), 5.94 (d, 1 H, H-1′, *J*_1′,2′_ = 3.8), 5.72 (dd, 1 H, H-5, *J*_NH,5_ = 1.2, *J*_5,6_ = 7.6 ), 4.59–4.46 (m, 3 H, H-2′, H-4′, H-5′a), 4.00–3.90 (m, 1 H, H-5′b) 3.88 (d, 1 H, H-3′, *J*_3′,4′_ = 2.4), 3.68–3.59 (m, 2 H, H-1″a), 3.47–3.37 (ddd, H-1″b), 1.64–1.51 (m 2 H, C*H*_2_-2″), 1.45 (s, 3 H, C*H*_3_, *i*-Pr), 1.37–1.16 (m, 21 H, C*H*_2_-3″ to C*H*_2_-11″, C*H*_3_, *i*-Pr), 0.86 (t, 3 H, C*H*_3_-12″, *J* = 6.7). ^13^C NMR (CDCl_3_, 400 MHz): δ 163.6 (C-4), 153.0 (C-2), 139.1 (C-6), 111.7 (Cq, *i*-Pr), 105.2 (C-1′), 102.0 (C-5), 83.3 (C-3′), 82.4 (C-2′), 78.2 (C-4′), 70.6 (*C*H_2_-1″), 40.1 (C-5′), 32.0 (*C*H_2_-2″), 29.8, 29.8, 29.8, 29.8, 29.7, 29.6, 29.5, 26.8 (C*H*_2_-3″ to C*H*_2_-10″), 26.3, 26.2 (2 × *C*H_3_, *i*-Pr), 22.8 (*C*H_2_-11″), 14.3 (*C*H_3_-12″). HRMS: calcd for C_24_H_40_N_2_O_6_ [M + Na]^+^ 475.2779, found 475.2788; calcd for C_24_H_40_N_2_O_6_ [M + H]^+^ 453.2959, found 453.2969.

Compound **8**: ${\left[\alpha \right]}_{D}^{20}$ = −4 (c = 1, in CH_2_Cl_2_). ^1^H NMR (CDCl_3_, 400 MHz): *δ* 7.24 (d, 1 H, H-6, *J*_5,6_ = 7.9), 5.93, 5.90 (2 d, 2 H, H-1′, H-1″, *J*_1′,2′_ = 3.8, *J*_1″,2″_ = 3.8 ), 5.69 (d, 1 H, H-5), 4.63–4.46 (m, 4 H, H-5″a, H-2′ H-2″, H-4″), 4.44 (ddd, 1 H, H-4′), 4.31 (dd, 1 H, H-5′a, *J*_5′a,5′b_ = 14.7, *J*_4′,5′a_ = 2.6), 3.92 (dd, 1 H, H-5″b, *J*_5″a,5″b_ = 13.8, *J*_4″,5″a_ = 1.6), 3.86 (d, 1 H, H-3″, *J*_3″,4″_ = 3.3), 3.82 (d, 1 H, H-3′, *J*_3′,4′_ = 3.1), 3.68–3.55 (m, 3 H, H-5’b, H-1′′′a, H-1″″a), 3.46–3.32 (m, 2 H, H-1′′′b, H-1″″b), 1.60–1.49 (m 4 H, C*H*_2_-2′′′, C*H*_2_-2″″) 1.45, 1.43 (2 s, 2 × 3 H, 2 × C*H*_3_, *i*-Pr), 1.35–1.16 (m, 42 H, C*H*_2_-3′′′ to C*H*_2_-11′′′, C*H*_2_-3″″ to C*H*_2_-11″″, 2 × C*H*_3_, *i*-Pr), 0.86 (t, 3 H, C*H*_3_-12′′′, C*H*_3_-12″″, *J* = 6.7). ^13^C NMR (CDCl_3_, 100MHz): δ 163.2 (C-4), 151.9 (C-2), 143.4 (C-6), 112.0, 111.5 (Cq, *i*-Pr), 105.3, 105.3, (C-1′, C-1″), 101.8 (C-5), 83.5 (C-3″), 82.9 (C-3′), 82.4, 82.1 (C-2′, C-2″), 78.3 (C-4′), 78.1 (C-4″), 70.5 (*C*H_2_-1′′′, *C*H_2_-1″″), 49.0 (C-5′), 40.8 (C-5″), 32.0 (*C*H_2_-2′′′, *C*H_2_-2″″), 29.8, 29.8, 29.8, 29.7, 29.7, 29.7, 29.7, 29.6, 29.5, 29.5, 29.5, 26.8 (C*H*_2_-3′′′ to C*H*_2_-10′′′, C*H*_2_-3″″ to C*H*_2_-10″″), 26.3, 26.3, 26.2, 26.2 (4 × *C*H_3_, *i*-Pr), 22.8 (*C*H_2_-11′′′, *C*H_2_-11″″), 14.3 (*C*H_3_-12′′′, *C*H_3_-12″″). HRMS: calcd for C_44_H_76_N_2_O_10_ [M + Na]^+^ 815.5392, found 815.5420; calcd for C_44_H_76_N_2_O_10_ [M + H]^+^ 793.5573, found 793.5584.

Compound **9**: ${\left[\alpha \right]}_{D}^{20}$ = −31 (c = 1, in CH_2_Cl_2_). ^1^H NMR (CDCl_3_, 400 MHz): *δ* = 8.18 (d, 1H, H-6, *J* = 5.7), 6.41 (d, 1 H, H-5), 5.97–5.92 (m, 2 H, H-1′, H-1″, *J* = 3.9, *J* = 4.2), 4.69 (dd, 1H, H-5′a, *J*_5a,5b_ = 10.5, *J*_4,5a_ = 3.9), 4.63–4.42 (m, 7 H, H-2′, H-2″, H-4′, H-4″, H-5′b, H-5″a, H-5″b), 3.95, 3.89 (2 d, 2 × 1 H, H-3′, H-3″, *J* = 2.2 Hz, *J* = 2.8), 3.66–3.52 (m, 2 H, H-1′′′a, H-1″″a), 3.48–3.36 (m, 2 H, H-1′′′b, H-1″″b), 1.59–1.45 (m, 10 H, 2 × C*H*_3,_ *i*-Pr, C*H*_2_-2′′′, C*H*_2_-2″″); 1.36–1.14 (m, 42 H, 2 × C*H*_3_, *i*-Pr, C*H*_2_-3′′′ to C*H*_2_-11′′′, C*H*_2_-3′′′ to C*H*_2_-11″″); 0,87 (t, 6 H, C*H*_3_-12′′′, C*H*_3_-12″″, *J* = 6.5) ppm. ^13^C NMR (CDCl_3_, 400 MHz): δ 170.9 (C-4), 164.8 (C-2), 158.7 (C-6), 111.9, 111.8 (2 × Cq, *i*-Pr), 105.4, 105.3 (C-1′, C-1″), 102.54 (C5), 82.7, 82.6, 82.4, 82.4 (C-2′, C-2″, C-3′, C3″), 78.4, 78.3 (C-4′, C4″), 70.8, 70.7 (*C*H_2_-1′′′/ *C*H_2_-1″″), 64.7, 64.4 (C-5′, C-5″), 32.1, 29.8, 29.8, 29.7, 29.7, 29.5, 27.0, 26.5, 26.4, 26.2, 26.1 (4 × *C*H_3_, *i*-Pr, *C*H_2_-2′′′ to *C*H_2_-10′′′, *C*H_2_-2″″ to *C*H_2_-10″″), 22.8 (*C*H_2_-11′′′, *C*H_2_-11″″), 14.4 (*C*H_3_-12′′′, *C*H_3_-12″″) ppm. HRMS: calcd for C_44_H_76_N_2_O_10_ [M + H]^+^ 793.5573, found 793.5592; calcd for C_44_H_76_N_2_O_10_ [M + Na]^+^ 815.5392, found 815.5414. 

#### 4.1.8. 1-(5-Deoxy-3-*O*-dodecyl-α,β-D-xylofuranos-5-yl)-3,7-dimethyl-3,7-dihydro-1*H*-purine-2,6-dione (**10**)

A solution of 1-(5-deoxy-3-*O*-dodecyl-1,2-*O*-isopropylidene-α-D-xylofuranos-5-yl)-3,7-dimethyl-3,7-dihydro-1*H*-purine-2,6-dione (**3**, 39 mg, 0.075 mmol) in aq. trifluoroacetic acid (TFA, 60%, 3 mL) was stirred at room temp. for 2 h. The solvents were co-evaporated with toluene and the residue was subjected to column chromatography (from AcOEt/hexane, 10:1 to AcOEt/MeOH, 12:1) to afford **10** (30 mg, 83%, anomeric mixture, α/β ratio, 1:0.9) as colorless oil. ^1^H NMR (400 MHz, CDCl_3_) *δ*: 7.54 (s, 1.9 H, H-8 α, β), 5.56 (br.s, 1 H, H-1′ α), 5.11 (1, 0.9 H, H-1′ β), 4.67–4.38 (m, 3.8 H, H-4′ α, H-4′ β, H-5′a α, H-5′a β), 4.34–4.19 (m, 1.9 H, H-2′ α, H-2′ β), 4.08–3.90 (m, 9.5 H, H-3′ α, H-3′ β, H-5′b α, H-5’b β, C*H*_3_-N^7^ α, β), 3.71–3.42 (m, 9.5 H, C*H*_2_-1″ α, C*H*_2_-1″ β, C*H*_3_-N^3^ α, β) 1.70–1.60 (m 3.8 H, C*H*_2_-2″ α, C*H*_2_-2″ β), 1.37–1.20 (m, 34.2 H, C*H*_2_-3″ to C*H*_2_-11″), 0.87 (t, 5.7 H, C*H*_3_-12″, *J* = 6.7). ^13^C NMR (100 MHz, CDCl_3_) *δ*: 155.9 (C-6 α, β), 152.0, 151.9 (C-2 α, β), 149.1, 148.9 (C-4 α, β), 142.1 (C-8 α, β), 107.9, 107.8 (C-5 α, β), 103.5 (C-1′ β), 96.4 (C-1′ α), 84.6 (C-3’ β), 84.1 (C-3’ α), 80.1 (C-2′ α), 76.8 (C-4′ α, β), 75.2 (C-2′ β), 71.5 (*C*H_2_-1″ α), 70.9 (*C*H_2_-1″ β), 43.0 (C-5′, α, β), 33.8, 33.7 (*C*H_3_, N^7^, α, β), 32.1 (*C*H_2_-2″, α, β), 30.1, 29.9, 29.9, 29.8, 29.8, 29.8, 29.8, 29.7, 29.6, 29.6, 29.5, 26.2, 22.8, (*C*H_3_, N^3^, C*H*_2_-3″ to C*H*_2_-11″, α, β), 14.3 (*C*H_2_-12″, α, β). HRMS: calcd for C_24_H_40_N_4_O_6_ [M + Na]^+^ 503.2840, found 503.2836; calcd for C_24_H_40_N_4_O_6_ [M + H]^+^ 481.3021, found 481.3018.

#### 4.1.9. 1-(1,2-Di-*O*-acetyl-5-deoxy-3-*O*-dodecyl-α,β-D-xylofuranos-5-yl)-3,7-dimethyl-3,7-dihydro-1*H*-purine-2,6-dione (**11**)

A solution of 1-(5-deoxy-3-*O*-dodecyl-α,β-D-xylofuranos-5-yl)-3,7-dimethyl-3,7-dihydro-1*H*-purine-2,6-dione (**10**, 41 mg, 0.078 mmol) in pyridine (3 mL) and acetic anhydride (1.5 mL) was stirred at room temp. for 1.5 h. After co-evaporation of the solvents with toluene, the residue was subjected to column chromatography (AcOEt/hexane, 5:1) to give **11** (33 mg, 69%, anomeric mixture, α/β ratio, 1:0.5) as a colorless oil. ^1^H NMR (400 MHz, CDCl_3_) *δ*: 7.50, 7.48 (2 s, 1.5 H, H-8 α, β), 6.38 (dd, 1 H, H-1′ α, *J*_1’,2’__α_ = 4.5), 5.99 (1, 0.5 H, H-1′ β), 5.27 (t, 1 H, H-2′ α), 5.23 (s, 0.5 H, H-2′ β), 4.84–4.58 (m, 3 H, H-4′ α, H-4′ β, H-5′a α, H-5′a β), 4.19 (t, 1 H, H-3, *J*_2’,3’__α_ ~ *J*_2’,4’__α_ = 5.5), 4.01 (br.d, 0.5 H, H-3′ β, *J*_3’,4’__β_ = 5.0), 3.99–3.84 (m, 6 H, C*H*_3_-N^7^ α, β, H-5′b α, H-5′b β), 3.74–3.65 (m, 0.5 H, H-1′′a β), 3.65–3.44 (m, 7 H, C*H*_2_-1″ α, H-1″b β, C*H*_3_-N^3^ α, β), 2.18 (s, 1.5 H, CH_3_, Ac, β), 2.05 (s, 4.5 H, CH_3_, Ac, α, β), 1.99 (s, 3 H, CH_3_, Ac, α), 1.63–1.50 (m, 3 H, C*H*_2_-2″, α, β), 1.41–1.16 (m, 27 H, C*H*_2_-3″ to C*H*_2_-11″), 0.85 (t, 4.5 H, C*H*_3_-12″, *J* = 6.8). ^13^C NMR (100 MHz, CDCl_3_) *δ*: 170.5, 169.8 (CO, Ac, β), 169.7, 169.4 (CO, Ac, α), 155.3, 155.3 (C-6 α, β), 151.7 (C-2 α, β), 148.9, 148.8 (C-4 α, β), 141.6, 141.4 (C-8 α, β), 107.8 (C-5 α, β), 99.0 (C-1′ β), 84.2 (C-1′ α), 82.0 (C-3’ β), 80.7 (C-3’ α), 80.2 (C-4′ β), 79.8 (C-2′ β), 76.5 (C-2’ α), 76.2 (C-4′ α), 71.2, 71.0 (*C*H_2_-1″, α, β), 41.9 (C-5′ β), 41.3 (C-5′ α), 33.7, 33.7 (*C*H_3_, N^7^, α, β), 32.0 (*C*H_2_-2″, α, β), 29.9, 29.8, 29.8, 29.8, 29.7, 29.7, 29.6, 29.5, 26.3, 26.2, 22.8, (*C*H_3_, N^3^, C*H*_2_-3″ to C*H*_2_-11″, α, β), 21.5, 21.0, 20.9, 20.7 (*C*H_3_, Ac, α, β), 14.2 (*C*H_2_-12″, α, β).

#### 4.1.10. *N*-[2-*O*-Acetyl-1,5-dideoxy-5-(3,7-dimethyl-3,7-dihydro-2,6-dioxo-1*H*-purin-1-yl)-3-*O*-dodecyl-α,β-D-xylofuranos-1-yl]methanesulfonamide (**12**)

To a solution of 1-(1,2-di-*O*-acetyl-5-deoxy-3-*O*-dodecyl-α,β-D-xylofuranos-5-yl)-3,7-dimethyl-3,7-dihydro-1*H*-purine-2,6-dione (**11**, 22 mg, 0.039 mmol) in CH_2_Cl_2_/acetonitrile (1.2 mL, 4:1) under nitrogen and at 0 °C, BF_3_·Et_2_O (0.03 mL, 0.24 mmol) and methanesulfonamide (21 mg, 0.22 mmol) were added. The mixture was stirred at room temp. for 2 h. Then, it was diluted with CH_2_Cl_2_ and washed with a satd. aq. NaHCO_3_ soln. The aqueous phase was extracted with CH_2_Cl_2_ (2×) and the combined organic layers were dried with anhydrous MgSO_4_. After filtration and evaporation of the solvent, the residue was subjected to column chromatography (AcOEt/hexane, 15:1) to afford **12** (19 mg, 81%, anomeric mixture, β/α ratio, 1:0.4) as a yellow oil. ^1^H NMR (400 MHz, CDCl_3_) *δ*: 7.52, 7.51 (2 s, 1.4 H, H-8 α, β), 5.73 (d, 1 H, NH, β, *J*_1’,NH β_ = 11.1), 5.61 (dd, 0.4 H, H-1′ α, *J*_1’,2’ α_ = 3.5, *J*_1’,NH α_ = 11.3), 5.23 (d, 1 H, H-1′ β), 5.20 (s, 1 H, H-2′ β), 5.14 (dd, 0.4 H, H-2′ α, *J*_1’,2’ α_ = 3.5, *J*_2’,3’ α_ = 0.9), 5.07 (d, 0.4 H, N*H* α), 4.72 (dd, 1 H, H-5′a β, *J*_5’a,5’b β_ = 13.9, *J*_4’,5’a β_ = 9.5), 4.63–4.45 (m, 2.2 H, H-5′a α, H-5′b α, H-4′ α, H-4′ β, *J*_5’a,5’b α_ = 13.8, *J*_4’,5’a α_ = 8.7), 4.02–3.91 (m, 6.6 H, H-3′ α, H-3′ β, H-5’b β, C*H*_3_-N^7^ α, β), 3.81–3.72 (m, 1.4 H, H-1′′a α, H-1″a β), 3.61–3.49 (m, 5.6 H, H-1″b α, H-1″b β, C*H*_3_-N^3^ α, β)_,_ 3.11 (s, 3 H, S-C*H*_3_, β), 3.03 (s, 1.2 H, S-C*H*_3_, α), 2.11 (s, 1.2 H, CH_3_, Ac, α), 2.07 (s, 3 H, CH_3_, Ac, β), 1.70–1.60 (m 2.8 H, C*H*_2_-2″ α, C*H*_2_-2″ β), 1.37–1.20 (m, 25.2 H, C*H*_2_-3″ to C*H*_2_-11″, α, β), 0.87 (t, 4.2 H, C*H*_3_-12″, α, β, *J* = 6.7). ^13^C NMR (100 MHz, CDCl_3_) *δ*: 169.6, 168.9 (CO, Ac, α, β) 155.3 (C-6 α, β), 151.7 (C-2 α, β), 149.1, 149.0 (C-4 α, β), 141.8, 141.7 (C-8 α, β), 107.7 (C-5 α, β), 88.0 (C-1′ β), 82.9 (C-1′ α), 81.5 (C-3’ α), 81.0 (C-3’ β), 79.4 (C-4′ β), 78.9 (C-2′ β), 71.8 (*C*H_2_-1″, α, β), 43.0 (S*C*H_3,_ β), 42.3, 41.7 (S*C*H_3,_ α, C-5′ β), 33.8 (*C*H_3_-N^7^, α, β), 32.0 (*C*H_2_-2″, α, β), 29.9, 29.9, 29.8, 29.8, 29.7, 29.7, 29.6, 29.6, 29.5, 26.1, 22.8 (*C*H_3_-N^3^, C*H*_2_-3″ to C*H*_2_-11″, α, β) 21.0, 21.0 (*C*H_3_, Ac, α, β), 14.3 (*C*H_2_-12″, α, β). HRMS: calcd for C_27_H_45_N_5_O_8_S [M + H]^+^ 600.3062, found 600.3058; calcd for C_27_H_45_N_5_O_8_S [M + Na]^+^ 622.2881, found 622.2878.

#### 4.1.11. 1-(2-*O*-Acetyl-1-azido-1,5-dideoxy-3-*O*-dodecyl-α-D-xylofuranos-5-yl)-3,7-dimethyl-3,7-dihydro-1*H*-purine-2,6-dione (**13**) and 1-(2-*O*-acetyl-5-deoxy-3-*O*-dodecyl-D-xylono-1,4-lacton-5-yl)-3,7-dimethyl-3,7-dihydro-1*H*-purine-2,6-dione (**14**)

To a solution of 1-(1,2-di-*O*-acetyl-5-deoxy-3-*O*-dodecyl-α,β-D-xylofuranos-5-yl)-3,7-dimethyl-3,7-dihydro-1*H*-purine-2,6-dione (**11**, 30 mg, 0.053 mmol) in acetonitrile (5 mL), trimethylsilyl azide (TMSN_3_, 0.06 mL, 0.43 mmol) and trimethylsilyl triflate (TMSOTf, 0.08 mL, 0.044 mmol) were sequentially added. The mixture was stirred at 65 °C under microwave irradion (150 W, *P*max = 250 Psi) for 1 h 40 min. The solution was then diluted with CH_2_Cl_2_ and washed with a sat. aq. NaHCO_3_ soln. The aqueous phase was extracted with CH_2_Cl_2_ (3×) and the combined organic phases were dried with anhydrous MgSO_4_. After filtration and evaporation, the residue was subjected to column chromatography (from AcOEt/cyclohexane, 1:1 to AcOEt) to afford **13-α** (11 mg, 38%) and a mixture (18 mg) containing **13-β** and the xylonolactone derivative **14** in a 3.3:1 ratio (corresponding to 14 mg of **13-β** (48%) and 4 mg of **14** (14%)).

Data for **13-α**: ${\left[\alpha \right]}_{D}^{20}$ = +74 (c = 1, in CH_2_Cl_2_). ^1^H NMR (400 MHz, CDCl_3_) *δ*: 7.52 (s, 1 H, H-8), 5.58 (d, 1 H, H′-1, *J*_1′,2′_ = 4.7), 5.12 (t, 1 H, H′-2, *J*_1′,2′_ ~ *J*_2′,3′_), 4.71–4.56 (m, 2 H, H′-4, H′-5a), 4.12 (t, 1 H, H′-3, *J*_2′,3′_ ~ *J*_3′,4′_ ~ 4.5), 3.99 (s, 3 H, C*H*_3-_N^7^), 3.91 (d, 1 H, H′-5b, *J* = 12.2), 3.66–3.53 (m, 4 H, H-1″a, C*H*_3-_N^3^), 3.54–3.45 (m, 1 H, H-1″b), 2.13 (s, 3 H, CH_3_, OAc), 1.62–1.52 (m, 2 H, C*H*_2_-2″), 1.38–1.18 (m, 18 H, C*H*_2_-3″ to C*H*_2_-11″), 0.87 (t, 3 H, C*H*_3_-12″, *J* = 6.7). ^13^C NMR (100 MHz, CDCl_3_) *δ*: 170.1 (CO, OAc), 155.4 (C-6), 151.7 (C-2), 149.0 (C-4), 141.7 (C-8), 107.8 (C-5), 89.6 (C-1′), 81.4 (C-3′), 77.7 (C′-2), 76.5 (C′-4); 71.2 (C-1″), 41.2 (C-5ʹ), 33.8 (*C*H_3_-N^7^), 32.1, 29.9, 29.9, 29.8, 29.8, 29.7, 29.6, 29.5, 26.2, 22.8 (*C*H_3_-N^3^, C*H*_2_-2″ to C*H*_2_-11″), 20.8 (*C*H_3,_ OAc), 14.3 (C-12″). HRMS: calcd for C_26_H_41_N_7_O_6_ [*M* + H]^+^ 548.3191, found 548.3187; calcd for C_26_H_41_N_7_O_6_ [M + Na]^+^ 570.3011, found 570.3004.

Data for **13-β**: ^1^H NMR (400 MHz, CDCl_3_) *δ*: 7.50 (s, 1 H, H-8), 5.11 (s, 1 H, Hʹ-2), 5.02 (s, 1 H, H′-1) 4.83 (dd, 1 H, H-5′a, *J*_5′a,5′b_ = 14.3, *J*_5′a,4′_ = 8.3), 4.59 (ddd, 1 H, H-4′), 4.07 (dd, 1 H, H-5′b, *J*_5′b,4′_ = 1.9), 3.99 (s, 3 H, C*H*_3-_N^7^), 3.91 (d, 1 H, H′-3, *J*_3′,4′_ = 4.3), 3.79–3.70 (m, 1 H, H-1″a), 3.58 (s, 3 H, C*H*_3-_N^3^), 3.54–3.45 (m, 1 H, H-1″b), 2.07 (s, 3 H, CH_3_, OAc), 1.65–1.56 (m, 2 H, C*H*_2_-2″), 1.44–1.17 (m, 18 H, C*H*_2_-3″ to C*H*_2_-11″), 0.86 (t, 3 H, C*H*_3_-12″, *J* = 6.7)*. ^13^C NMR (100 MHz, CDCl_3_) *δ*: 169,7 (CO, OAc), 155.3 (C-6), 151.8 (C-2), 149.0 (C-4), 141.6 (C-8), 107.8 (C-5), 93.3 (C-1′), 81.9 (C-4′), 81.4 (C′-3), 79.8 (C′-2); 70.9 (C-1″), 41.2 (C-5′), 33.8 (*C*H_3_-N^7^), 32.1, 29.9, 29.8, 29.8, 29.7, 29.6, 29.5, 26.2, 22.8 (*C*H_3_-N^3^, C*H*_2_-2″ to C*H*_2_-11″), 20.8 (*C*H_3,_ OAc), 14.3 (C-12″)*. HRMS: calcd for C_26_H_41_N_7_O_6_ [M + H]^+^ 548.3191, found 548.3186.

Data for **14:** ^1^H NMR (400 MHz, CDCl_3_) *δ*: 7.51 (s, 1 H, H-8), 5.59 (d, 1 H, H′-2, *J*_2′,3′_ = 6.7), 5.07 (ddd, 1 H, H′-4), 4.83 (dd, 1 H, H-5′a, *J*_5′a,5′b_ = 14.3, *J*_5′a,4′_ = 10.9), 4.37 (t, 1 H, H′-3, *J*_2′,3′_ ~ *J*_3′,4′_), 4.07 (dd, 1 H, H-5′b, *J*_5′b,4′_ = 3.0), 3.97 (s, 3 H, C*H*_3-_N^7^), 3.65–3.53 (m, 5 H, C*H*_3-_N^3^, C*H*_2_-1″), 2.17 (s, 3 H, CH_3_, OAc), 1.66–1.54 (m, 2 H, C*H*_2_-2″), 1.47–1.15 (m, 18 H, C*H*_2_-3″ to C*H*_2_-11″), 0.87 (t, 3 H, C*H*_3_-12″, *J* = 6.7)*. ^13^C NMR (100 MHz, CDCl_3_) *δ*: 170.0 (C-1′), 169.4 (CO, Ac), 155.1 (C-6), 151.6 (C-2), 149.1 (C-4), 141.8 (C-8), 107.7 (C-5), 78.7 (C-3′), 75.9 (C-4′), 72.2 (C′-2), 71.3 (C-1″), 40.5 (C-5′), 33.8 (*C*H_3_-N^7^), 32.1, 29.9, 29.8, 29.8, 29.7, 29.5, 29.5, 26.1, 22.8 (*C*H_3_-N^3^, C*H*_2_-2″ to C*H*_2_-11″), 20.7 (*C*H_3,_ OAc), 14.3 (C-12″)*. HRMS: calcd for C_26_H_40_N_4_O_7_ [M + H]^+^ 521.2970, found 521.2966.

* Data extracted from the spectrum of a mixture containing **13-β/14**.

#### 4.1.12. Dimethyl *N*-[2-*O*-acetyl-1,5-dideoxy-3-*O*-dodecyl -5-(3,7-dimethyl-3,7-dihydro-2,6-dioxo-1*H*-purin-1-yl)-α,β-D-xylofuranos-1-y]phosphoramidate (**15**)

To a solution of 1-(2-*O*-acetyl-1-azido-1,5-dideoxy-3-*O*-dodecyl-α-D-xylofuranos-5-yl)-3,7-dimethyl-3,7-dihydro-1*H*-purine-2,6-dione (**13-α**, 11 mg, 0.02 mmol) in CH_2_Cl_2_ (2 mL), trimethyl phosphite (0.03 mL, 0.25 mmol) was added. The solution was stirred at room temperature for 23 h. The solution was then concentrated under vacuum and the residue was subjected to column chromatography (from AcOEt/hexane, 15:1 to AcOEt/MeOH, 15:1) to afford **15** (7 mg, 56%, anomeric mixture, α/β ratio, 1:0.3) as a colorless oil. ^1^H NMR (400 MHz, CDCl_3_) *δ*: 7.50 (s, 1.3 H, H-8 α, β), 5.33 (ddd, 1 H, H-1′ α, *J*_1′,2′ α_ = 3.9 Hz, *J*_1′,P_ α = 7.5 Hz, *J*_1′,N*H*_ α = 11.6), 5.14 (s, 0.3 H, H-2′ β), 5.06 (d, 1 H, H-2′ α, *J*_1′,2′ α_ = 3.4 Hz), 4.96 (dd, 0.3 H, H-1′ β, *J*_1′,P β_ = 7,8 Hz, *J*_1′,NH β_ = 11.2), 4.73–4.57 (m, 1.3 H, H-5′a, α, β, *J*_5′a,5′b α_ = 13.9, *J*_4′,5′a α_ = 9.1, *J*_5′a,5′b β_ = 13.8, *J*_4′,5′a β_ = 8.9), 4.54–4.42 (m, 1.3 H, H-4′, α, β), 4.15 (t, 0.3 H, N*H*, β, *J*_1′,NH β_ ~ *J*_NH,P β_ = 11.2 Hz), 4.05–3.85 (m, 6.5 H, H-5′b α, β, H-3′ α, β, C*H*_3_-N^7^ α, β), 3.77–3.46 (m, 15.6 H, 2 × OC*H*_3_ α, 2 × OC*H*_3_ β, C*H*_2_-1″ α, β, H-3′ α, β, C*H*_3_-N^7^ α, β), 2.11 (s, 3 H, C*H*_3_, Ac, α), 2.06 (s, 0.9 H, Ac, β), 1.66–1.54 (m, 2.6 H, C*H*_2_-2″ α, β), 1.38–1.17 (m, 23.4 H, C*H*_2_-3″ to C*H*_2_-11″ α, β), 0,87 (t, 3.9 H, C*H*_2_-12″ α, β, *J* = 6.7). ^13^C NMR (100 MHz, CDCl_3_) *δ*: 169.9, 169.7 (CO, Ac α, β), 155.4 (C-6 α, β), 151.7 (C-2 α, β); 149.0, 148.9 (C-4 α, β), 141.6, 141.5 (C-8 α, β), 107.8 (C-5 α, β), 87.4 (C-1′ β), 82.4 (C-1ʹ α), 82.2, 81.5 (C-3′ α, β), 79.9 (C-2′ β); 78.4 (C-4′ α or β), 76.2, 76.0, 76.0 (C-2′ α, C-4′ α or β), 71.4, 71.3 (*C*H_2_-1″ α, β); 53.4, 53.4, 53.3, 53.2 (4 d, 2 × O*C*H_3_ α, β, *J*_C,P_ = 4.8), 41.7, 40-8 (C-5′ α, β), 33.7 (*C*H_3_-N^7^ α, β), 32.1, 29.8, 29.8, 29.8, 29.6, 29.6, 29.5, 26.2, 26.2, 22.8 (*C*H_3_-N^3^ α, β, C*H*_2_-2″ to C*H*_2_-11″), 21.1, 21.0 (*C*H_3_, Ac, β), 14.3 (C*H*_3_-12″ α, β). ^31^P NMR (162 MHz, CDCl_3_) *δ*: 8.49. HRMS: calcd for C_28_H_48_N_5_O_9_P [M + H]^+^ 630.3262, found 630.3270; calcd for C_28_H_48_N_5_O_9_P [M + Na]^+^ 652.3082, found 652.3090.

#### 4.1.13. 3-*O*-Octyl-1,2-*O*-isopropylidene-α-D-glucofuranose (**16**)

To a solution of 1,2:5,6-di-*O*-isopropylidene-α-D-glucofuranose (**15**, 3.0 g, 11.53 mmol) in anhydrous DMF (30 mL) under nitrogen atmosphere and at 0 °C and, NaH (60%, 0.69 g, 17.25 mmol) was added. The suspension was stirred at 0 °C for 10 min. Then, octyl bromide (2.38 mL, 13.79 mmol) was added and the mixture was stirred at room temperature for 22 h. It was then diluted with CH_2_Cl_2_ and washed with water and brine solution. The aqueous phase was extracted with CH_2_Cl_2_ (3×) and the combined organic layers were dried with anhydrous MgSO_4_, filtered and concentrated. To the resulting residue, aq. acetic acid (70% soln., 38 mL) was added and the resulting solution was stirred at room temperature for 26 h. After co-evaporation with toluene, the residue was subjected to column chromatography (EtOAc/hexane, 1:3) to afford 16 (3.37 g, 88%, 2 steps) as a colorless oil. ^1^H NMR (CDCl_3_, 400 MHz): 5.82 (d, 1 H, H-1, *J*_1,2_ = 3.7), 4.47 (d, 1 H, H-2), 4.03 (dd, 1 H, H-4, *J*_3,4_ = 3.2, *J*_4,5_ = 7.8), 3.95–3.85 (m, 2 H, H-3, H-5), 3.73 (dd, part A of AB system, H-6a, *J*_5,6a_ = 3.0, *J*_6a,6b_ = 11.6), 3.63 (dd, part B of AB system, H-6b, *J*_5,6b_ = 5.6), 3.59–3.49 (m, 1 H, H-1′a), 3.49–3.14 (m, 3 H, H-1′b, O*H*-5, O*H*-6), 1.57–1.44 (m, 2 H, C*H*_2_-2′), 1.41 (s, 3 H, C*H*_3_, i-Pr), 1.31–1.12 (m, 13 H, C*H*_2_-3′ to C*H*_2_-7′, C*H*_3_, *i*-Pr), 0.81 (t, 3 H, C*H*_3_-8′, *J* = 6.6). ^13^C NMR (CDCl_3_, 100 MHz): 111.6 (Cq, *i*-Pr), 105.0 (C-1), 82.6 (C-3), 82.1 (C-2), 79.7 (C-4), 70.6 (C-1′), 69.3 (C-5), 64.2 (C-6), 31.8, 29.7, 29.3, 29.2, 26.7, 26.2, 26.0, 22.6 (C-2′ to C-7′, 2 × *C*H_3_, i-Pr), 14.1 (C-8′). HRMS: calcd for C_17_H_32_O_6_ [M + H]^+^ 333.2272, found 333.2266; calcd for C_17_H_32_O_6_ [M + Na]^+^ 355.2091, found 355.2081.

#### 4.1.14. 3-*O*-Octyl-1,2-*O*-isopropylidene-α-D-xylofuranose (**17**)

To a solution of 3-*O*-octyl-1,2-*O*-isopropylidene-α-D-glucofuranose (**16**, 3.14 g, 9.45 mmol) in 60% aq. THF (22 mL), at 0 °C, sodium metaperiodate (4.85 g, 22.68 mmol) was added. The mixture was stirred for 4 h at room temperature. Then, it was diluted with EtOAc and washed with water and brine solution. The aqueous phase was extracted with EtOAc (3×) and the combined organic layers were dried with anhydrous MgSO_4_. After filtration and concentration under vacuum, the residue was dissolved in EtOH/H_2_O (57 mL, 2:1). To the resulting solution at 0 °C, NaBH_4_ (0.461 g, 12 mmol) was added and the mixture was stirred at room temperature for 1.5 h. Then, EtOAc was added. The mixture was washed with brine soln. and the aqueous phase was extracted with EtOAc (2×). The combined organic layers were dried with anhydrous MgSO_4_, filtered and concentrated. The residue was subjected to column chromatography (EtOAc/petroleum ether, 2:1) to afford **17** (1.85 g, 65%, 2 steps) as a colorless oil. ^1^H NMR (CDCl_3_, 400 MHz): 5.98 (d, 1 H, H-1, *J*_1,2_ = 3.8), 4.57 (d, 1 H, H-2), 4.27 (br. q, 1 H, H-4), 4.00–3.85 (m, 2 H, H-3, H-5a, H-5b, *J*_3,4_ = 3.3, *J*_4,5a_ = 4.6, *J*_4,5b_ = 4.4, *J*_5a,5b_ = 12.3), 3.68–3.58 (m, 1 H, H-1′a), 3.47–3.38 (m, 1 H, H-1′b), 1.61–1.51 (m, 2 H, C*H*_2_-2′), 1.49 (s, 3 H, C*H*_3_, i-Pr), 1.38–1.19 (m, 13 H, C*H*_2_-3′ to C*H*_2_-7′, C*H*_3_, *i*-Pr), 0.88 (t, 3 H, C*H*_3_-8′, *J* = 6.6). ^13^C NMR (CDCl_3_, 100 MHz): 111.8 (Cq, *i*-Pr), 105.2 (C-1), 84.5 (C-3), 82.5 (C-2), 79.9 (C-4), 70.6 (C-1′), 61.3 (C-5), 31.9, 29.8, 29.5, 29.3, 27.0, 26.5, 26.2, 22.8 (C-2′ to C-7′, 2 × *C*H_3_, i-Pr), 14.2 (C-8′). HRMS: calcd for C_16_H_30_O_5_ [M + H]^+^ 303.2166, found 303.2156; calcd for C_16_H_30_O_5_ [M + Na]^+^ 325.1985, found 325.1974.

#### 4.1.15. 1-(5-Deoxy-3-*O*-dodecyl-α-D-xylofuranos-5-yl)-3,7-dimethyl-3,7-dihydro-1*H*-purine-2,6-dione (**18**)

A solution of 3-*O*-octyl-1,2-*O*-isopropylidene-α-D-xylofuranose (**17**, 80 mg, 0.26 mmol) in DMF (2 mL) was subjected to the MW-assisted protocol for Mitsunobu coupling, according to the general procure, using PPh_3_ (138 mg, 0.526 mmol), DEAD (40 % wt soln in toluene, 0.2 mL, 0.44 mmol) and theobromine (95 mg, 0.527 mmol). The mixture was stirred for 30 min. Column chromatography was performed using (AcOEt/petroleum ether, 2:1 as eluent. To the obtained residue, aq. TFA (60% soln., 3 mL) was added and the resulting solution was stirred at room temperature for 4 h. After co-evaporation of the solvents with toluene, the residue was subjected to column chromatography (from AcOEt/petroleum ether, 20:1 to AcOEt/MeOH, 12:1) to afford **10** (22 mg, 20%, 2 steps, anomeric mixture, α/β ratio, 1:1) as a colorless oil. ^1^H NMR (400 MHz, CDCl_3_) *δ*: 7.54, 7.51 (s, 2 H, H-8 α, β), 5.56 (d, 1 H, H-1′ α, *J*_1′,2′α_ = 4.0), 5.11 (d, 1 H, H-1′ β, *J*_1′,2′β_ = 1.5), 4.68–4.41 (m, 4 H, H-4′ α, H-4′ β, H-5′a α, H-5′a β), 4.30 (dd, 1 H, H-2′ β, *J*_2′,3′β_ = 3.6), 4.22 (br.t, 1 H, H-2′ α), 4.08–3.91 (m, 10 H, H-3′ α, H-3′ β, H-5′b α,H-5’b β, C*H*_3_-N^7^ α, β), 3.71–3.61 (m, 2 H, H-1″a α, H-1″a β), 3.60–3.43 (m, 8 H, H-1″b α, H-1″b β, C*H*_3_-N^3^ α, β) 1.66–1.50 (m 4 H, C*H*_2_-2″ α, C*H*_2_-2″ β), 1.41–1.16 (m, 20 H, C*H*_2_-3″ to C*H*_2_-7″), 0.87 (t, 6 H, C*H*_3_-8″, *J* = 6.6). ^13^C NMR (100 MHz, CDCl_3_) *δ*: 155.9, 155.5 (C-6 α, β), 152.0, 151.9 (C-2 α, β), 149.2, 149.0 (C-4 α, β), 142.0, 141.8 (C-8 α, β), 107.9, 107.8 (C-5 α, β), 103.4 (C-1′ β), 96.2 (C-1′ α), 84.6 (C-3’ β), 84.2 (C-3’ α), 80.3 (C-2′ β), 77.1 (C-4′ α, β), 75.3 (C-2′ α), 71.5 (*C*H_2_-1″ α), 70.9 (*C*H_2_-1″ β), 43.1, 41.5 (C-5′, α, β), 33.8, 33.8 (*C*H_3_, N^7^, α, β), 32.0 (*C*H_2_-2″, α, β), 30.0, 30.0, 29.9, 29.6, 29.4, 29.4, 26.2, 26.2, 22.8, (*C*H_3_, N^3^, C*H*_2_-3″ to C*H*_2_-7″, α, β), 14.3 (*C*H_2_-8″, α, β). HRMS: calcd for C_20_H_32_N_4_O_6_ [M + H]^+^ 425.2395, found 425.2390; calcd for C_20_H_32_N_4_O_6_ [M + Na]^+^ 447.2214, found 447.2208.

#### 4.1.16. 1-(6-Deoxy-3-*O*-dodecyl-1,2-*O*-isopropylidene-α-D-glucofuranos-6-yl)-3,7-dimethyl-3,7-dihydro-1*H*-purine-2,6-dione (**19**)

A solution of 3-*O*-octyl-1,2-*O*-isopropylidene-α-D-glucofuranose (**16**, 80 mg, 0.241 mmol) in DMF (2 mL) was subjected to the protocol for Mitsunobu coupling, according to the general procure, using PPh_3_ (138 mg, 0.526 mmol), DEAD (40 % wt soln in toluene, 0.24 mL, 0.526 mmol) and theobromine (95 mg, 0.527 mmol). The mixture was stirred under reflux for 20 h. Purification by column chromatography (from AcOEt/petroleum ether 3:1 to 4:1) afforded **19** (19 mg, 16%; isolated yield: 11 mg, 9%) as a colorless oil. ^1^H NMR (CDCl_3_, 400 MHz): 7.51 (s, 1 H, H-8), 5.97 (d, 1 H, H-1′, *J*_1,2_ = 3.7), 4.54 (d, 1 H, H-2′), 4.43 (dd, part A of AB system, H-6′a, *J*_5′,6′a_ = 7.3, *J*_a,b_ = 17.4), 4.29–4.19 (m, 2 H, H-5′, H-6′b), 4.11 (dd, 1 H, H-4′, *J*_3′,4′_ = 3.2, *J*_4′,5′_ = 6.3), 4.03 (d, 1 H, H-3′), 3.98 (s, 3 H, C*H*_3_-N^7^), 3.67–3.47 (m, 5 H, C*H*_2_-1″, C*H*_3_-N^3^), 1.72–1.52 (m, 2 H, C*H*_2_-2″), 1.50 (s, 3 H, C*H*_3_, *i*-Pr), 1.36–1.16 (m, 13 H, C*H*_2_-3″ to C*H*_2_-7″, C*H*_3_, *i*-Pr), 0.86 (t, 3 H, C*H*_3_-8″, *J* = 6.6). ^13^C NMR (CDCl_3_, 100 MHz): 153.4 (C-6), 149.9 (C-4), 141.7 (C-8), 111.7 (Cq, *i*-Pr), 105.5 (C-1′), 83.1 (C-3′), 82.4 (C-2′), 81.3 (C-4′), 70.9 (C-1″), 69.0 (C-5′), 45.2 (C-6′), 33.8 (*C*H_3_, N^7^), 31.9, 30.0, 29.9, 29.8, 29.6, 29.5, 29.3, 27.0, 26.5, 26.2, 22.8 (*C*H_3_, N^3^, C-2″ to C-7″, 2 × *C*H_3_, i-Pr), 14.2 (C-8″). HRMS: calcd for C_24_H_38_N_4_O_7_ [M + H]^+^ 495.2813, found 495.2808; calcd for C_24_H_38_N_4_O_7_ [M + Na]^+^ 517.2633, found 517.2626.

#### 4.1.17. 1-(6-Deoxy-3-*O*-dodecyl-α-D-glucopyranos-6-yl)-3,7-dimethyl-3,7-dihydro-1*H*-purine-2,6-dione (**20**)

A solution of 1-(6-deoxy-3-*O*-dodecyl-1,2-*O*-isopropylidene-α-D-glucofuranos-6-yl)-3,7-dimethyl-3,7-dihydro-1*H*-purine-2,6-dione **(19**, 20 mg, 0.04 mmol) in aq. trifluoroacetic acid (TFA, 60%, 3 mL) was stirred at room temp. for 5 h. The solvents were co-evaporated of with toluene and the residue was subjected to column chromatography (from AcOEt/petroleum ether 15:1 to AcOEt/MeOH, 20:1) to afford **20** (16 mg, 87%, anomeric mixture, α/β ratio, 1:0.9) as colorless oil. ^1^H NMR (CDCl_3_, 400 MHz): 7.62, 7.61 (2 s, 1.9 H, H-8, α, β), 5.28 (br.s, 1 H, H-1′α), 4.61 (d, 0.9 H, H-1′β, *J*_1′,2′__β_ = 7.5), 4.52 (dd, 0.9 H, H-6′a β, *J*_5′,6′a β_ = 2.1, *J*_6a,6b__β_ = 14.0), 4.45 (dd, 1 H, H-6′a α, *J*_5′,6′a α_ = 3.5, *J*_6a,6b__α_ = 14.4), 4.38–4.28 (m, 1.9 H, H-26′b α, H-6′b β), 4.13 (dt, 1 H, H-5′α, *J*_4′,5′__α_ = 9.7, *J*_5′,6′a__α_ = *J*_5′,6′b__α_ = 3.5), 4.01, 4.00 (2 s, 5.7 H, C*H*_3_-N^7^), 3.95–3.73 (m, 3.8 H, C*H*_2_-1″, α, β), 3.67–3.57 (s, 7.6 H, H-3′ β, H-4′ β, C*H*_3_-N^3^), 3.54–3.49 (m, 2.9 H, H-2′ α, H-3′ α, H-5 β), 3.46–3.01 (m, 7.6 H, H-′ β, H-4′ α, O*H*-1, O*H*-2, O*H*-4), 1.66–1.55 (m, 3.8 H, C*H*_2_-2″, α, β), 1.39–1.20 (m, 19 H, C*H*_2_-3″ to C*H*_2_-7″, α, β), 0.86 (t, 5.7 H, C*H*_3_-8″, α, β, *J* = 6.5). ^13^C NMR (CDCl_3_, 100 MHz): 156.0 (C-6 α, β), 152.4 (C-2 α, β), 149.0 (C-4 α, β), 142.1 (C-8 α, β), 107.7 (C-5 α, β), 107.7 (C-5 α, β), 97.2 (C-1′ β), 92.6 (C-1′ α), 83.5 (C-3′ β), 81.2 (C-3′ α), 75.1 (C-4′ β), 74.8 (C-2′ β), 73.8 (C-1″), 73.7 (C-5′ β), 72.2 (C-2′ α), 72.1 (C-2′ β), 72.1 (C-4′ α), 71.3 (C-5′ α), 41.6 (C-6′ α, β), 33.9 (*C*H_3_, N^7^, α, β), 32.0, 31.0, 30.2, 29.9, 29.6, 29.4, 26.2, 22.8 (*C*H_3_, N^3^, C-2″ to C-7″, α, β), 14.3 (C-8″, α, β). HRMS: calcd for C_21_H_34_N_4_O_7_ [M + H]^+^ 455.2500, found 455.2494; calcd for C_21_H_34_N_4_O_7_ [M + Na]^+^ 477.2320, found 477.2313.

#### 4.1.18. 1-(1,2,4-Tri-*O*-acetyl-6-deoxy-3-*O*-dodecyl-α-d-glucopyranos-6-yl)-3,7-dimethyl-3,7-dihydro-1*H*-purine-2,6-dione (**21**)

A solution of 1-(6-deoxy-3-*O*-dodecyl-α-D-glucopyranos-6-yl)-3,7-dimethyl-3,7-dihydro-1*H*-purine-2,6-dione (**20**, 27 mg, 0.059 mmol) in pyridine (1.6 mL) and acetic anhydride (1 mL) was stirred at room temp. for 2.5 h. After co-evaporation with toluene, the residue was subjected to column chromatography (AcOEt/hexane, 15:1 to AcOEt) to give **21** (29 mg, 84%, anomeric mixture, β/α ratio, 1:0.7) as a yellow oil. ^1^H NMR (CDCl_3_, 400 MHz): 7.53, 7.52 (2 s, 1.7 H, H-8, α, β), 6.24 (br.s, 0.7 H, H-1′α, *J*_1′,2′__α_ = 3.6), 5.51 (d, 1 H, H-1′β, *J*_1′,2′__β_ = 8.1), 5.16–4.97 (m, 3.4 H, H-2′α, H-2′β, H-4′α, H-4′β, *J*_2′,3′__α_ = 9.9, *J*_3′,4′__α_ = 9.8, *J*_2′,3′__β_ ~ *J*_3′,4′__β_ ~ *J*_4′,5′__β_ ~ 9.5), 4.63–4.49 (m, 1.7 H, H-6′a α, H-6′a β, *J*_6a,6b β_ = 13.8, *J*_6a,6b__α_ = 14.0, *J*_5,6a__α_ = 9.8, *J*_5,6a__β_ = 9.3), 4.33 (td, 0.7 H, H-5′ α, *J*_4′,5′ α_ = *J*_5′,6′a__α_ = 9.8, *J*_5′,6′b__α_ = 2.1), 4.04–3.94 (m, 6.1 H, C*H*_3_-N^7^, H-5′ β), 3.90–3.75 (m, 2.4 H, H-6′b α, H-6′b β, H-3 α), 3.66–3.50 (m, 9.5 H, C*H*_2_-1″, α, β, H-3′ β, C*H*_3_-N^3^), 2.18, 2.16, 2.08, 2.07, 2.04, 2.04 (6 s, 15.3 H, 3 x C*H*_3_, 3 × Ac, α, β), 1.55–1.42 (m, 3.4 H, C*H*_2_-2″, α, β), 1.37–1.19 (m, 17 H, C*H*_2_-3″ to C*H*_2_-7″, α, β), 0.86 (t, 5.1 H, C*H*_3_-8″, α, β, *J* = 6.7). ^13^C NMR (CDCl_3_, 100 MHz): 169.9, 169.6, 169.4. 169.1, 169.0 (CO, Ac, α, β), 155.2, 155.1 (C-6 α, β), 151.6, 151.6 (C-2 α, β), 149.0, 148.9 (C-4 α, β), 141.7, 141.7 (C-8 α, β), 107.7, 107.7 (C-5 α, β), 92.3 (C-1′ β), 89.6 (C-1′ α), 80.4 (C-3′ β), 77.0 (C-3′ α), 73.1, 72.9 (C-1″ α, β), 72.6 (C-5′ β), 72.0, 72.0, 71.6, 71.5 (C-2′, C-4′, α, β), 69.6 (C-5′ α), 42.0, 41.9 (C-6′ α, β), 33.8, 33.7 (*C*H_3_, N^7^, α, β), 32.0, 31.1, 30.4, 30.3, 29.9, 29.8, 29.6, 29.5, 29.4, 29.4, 26.1, 26.1, 22.8 (*C*H_3_, N^3^, C-2″ to C-7″, α, β), 21.1, 21.1, 20.9, 20.8 (*C*H_3_, Ac, α, β), 14.2 (C-8″, α, β). HRMS: calcd for C_27_H_40_N_4_O_10_ [M + H]^+^ 581.2817, found 581.2819; calcd for C_27_H_40_N_4_O_10_ [M + Na]^+^ 603.2637, found 603.2633.

#### 4.1.19. *N*-[2,4-Di-*O*-acetyl-1,6-dideoxy-6-(3,7-dimethyl-3,7-dihydro-2,6-dioxo-1*H*-purin-1-yl)-3-*O*-dodecyl-α,β-D-glucopyranos-1-yl]methanesulfonamide (**22**)

To a solution of 1-(1,2,4-tri-*O*-acetyl-6-deoxy-3-*O*-dodecyl-α-d-glucopyranos-6-yl)-3,7-dimethyl-3,7-dihydro-1*H*-purine-2,6-dione (**21**, 20 mg, 0.034 mmol) in CH_2_Cl_2_/acetonitrile (1.2 mL, 4:1) under nitrogen and at 0 °C, BF_3_·Et_2_O (0.02 mL, 0.16 mmol) and methanesulfonamide (19 mg, 0.19 mmol) were added. The mixture was stirred at room temp. for 3.5 h. Then, it was diluted with CH_2_Cl_2_ and washed with a satd. aq. NaHCO_3_ soln. The aqueous phase was extracted with CH_2_Cl_2_ (2×) and the combined organic layers were dried with anhydrous MgSO_4_. After filtration and evaporation of the solvent, the residue was subjected to column chromatography (AcOEt/petroleum ether, 10:1) to afford **22** (8 mg, 38%, anomeric mixture, β/α ratio, 1:0.4) as a yellow oil. ^1^H NMR (400 MHz, CDCl_3_) *δ*: 7.53 (s, 1.4 H, H-8 α, β), 6.21 (br.s, 0.4 H, N*H* α), 5.52 (d, 1 H, NH, β, *J*_1’,NH β_ = 9.6), 5.06–4.89 (m, 1.8 H, H-4′α, H-4′β, H-1 α), 4.82 (t,1 H, H-2 ′ β, *J*_1’,2’ β_ ~*J*_2’,3’ α_ ~ 9.3), 4.61–4.51 (m, 1.4 H, H-1′ β, H-2 α), 4.46 (dd, 1 H, H-6′a β, *J*_6a,6b β_ = 13.8, *J*_5,6a β_ = 9.8), 4.37–4.25 (m, 0.8 H, H-5′ α, H-6′a α), 3.89 (d, 1 H, H-6′b β), 3.98, 3.95 (2 s, 4.2 H, C*H*_3_-N^7^ α, β), 3.82–3.73 (m, 1.8 H, H-5′ β, H-6′b α, H-3′ α), 3.66–3.47 (m, 8 H, H-3′ β, C*H*_3_-N^3^ α, β, C*H*_2_-1″, α, β), 2.85 (s, 4.2 H, S-C*H*_3_, α, β), 2.18, 2.11, 2.06, 2.01 (s, 8.4 H, C*H*_3_, Ac, α), 1.52–1.42 (m 2.8 H, C*H*_2_-2″ α, C*H*_2_-2″ β), 1.36–1.14 (m, 14 H, C*H*_2_-3″ to C*H*_2_-7″, α, β), 0.88 (t, 4.2 H, C*H*_3_-8″, α, β, *J* = 6.6). ^13^C NMR (100 MHz, CDCl_3_) *δ*: 171.0, 170.1, 169.0 (CO, Ac, α, β), 155.1 (C-6 α, β), 151.5 (C-2 α, β), 149.2 (C-4 α, β), 142.1 (C-8 α, β), 107.6 (C-5 α, β), 83.1 (C-1′ β), 80.4 (C-3’ β), 74.3 (C-5′ β), 73.4 (*C*H_2_-1′′, α, β), 72.0 (C-2′ β), 71.8 (C-4′ β), 43.2 (S*C*H_3,_ β), 42.1, 42.1 (C-6′ β, C-6′ α), 42.0 (S*C*H_3,_ α), 33.7 (*C*H_3_, N^7^, α, β), 32.0, 30.3, 29.9, 29.9, 29.8, 29.6, 29.4, 26.1, 22.8 (*C*H_3_, N^3^, C-2″ to C-7″, α, β), 21.1, 21.0 (*C*H_3_, Ac, α), 14.2 (C-8″, α, β). HRMS: calcd for C_26_H_41_N_5_O_10_S [M + H]^+^ 616.2647, found 616.2645; calcd for C_26_H_41_N_5_O_10_S [M + Na]^+^ 638.2466, found 638.2464.

^1^H NMR and ^13^C NMR Spectra for compounds **3**–**12**, **13**-**α**, **15**–**22** can be found in the Supplementary Materials.

### 4.2. Biological Assays

#### 4.2.1. Cholinesterase Inhibition Assays

A TECAN Spectra-FluorPlus working on the kinetic mode and measuring the absorbance at λ = 415 nm was used for the enzymatic studies. Acetylcholinesterase (from *Electrophorus electricus*), 5,5′-dithiobis-(2-nitrobenzoic acid) (DTNB) and acetylthiocholine iodide were purchased from Fluka. Butyrylcholinesterase (from equine serum) was bought from Sigma. Experimental details for preparation of the solutions and the procedures for the enzyme assays can be found in the Supplementary Materials.

#### 4.2.2. Cytotoxicity Assays

The cytotoxicity of the compounds was evaluated using the sulforhodamine-B (SRB) colorimetric assay. The EC_50_ values in µM from SRB assays were determined after 96 h of treatment and were averaged from three independent experiments performed each in triplicate; confidence interval CI = 95%, cut-off the assay 30 µM. Compounds with EC_50_ > 30 µM are considered inactive. The cell lines were kindly provided by Th. Müller (Dep. of Haematology/Oncology, Martin Luther Universität Halle-Wittenberg). Human cancer cell lines: A375 (epithelial melanoma), A2780 (ovarian carcinoma), HT29 (colorectal adenocarcinoma), MCF7 (breast adenocarcinoma), SW1736 (thyroid carcinoma); non-malignant: NIH 3T3 (mouse embryonic fibroblasts).

### 4.3. Molecular Docking Studies

From the RCSB Protein Databank the crystal structures of AChE (PDB: 1C2O) and BChE (PDB: 6EMI) were selected. The compounds were built using Datawarrior and geometry optimized using MMFF94 force field. For the enzyme preparation, water and co-crystallized ligands were removed, gasteiger charges were added and non-polar hydrogens were merged with into the corresponding heavy atom.

The search space consisted of a 126 × 126 × 126 grid with 0.2 angstrom spacing centered on either the binding site of AChE or BChE. Dockings were executed with Lamarckian GA (200 population size; 25,000,000 evaluations; 15 runs). The pose showing the best binding energy (pose 1, ranked by energy) is shown in the manuscript.

## 5. Conclusions

In conclusion, purine/uracil 5′-isonucleosides and theobromine-containing 5′/6′-isonucleosides and isonucleotide analogs were synthesized in few steps from easily available *xylo*- or *gluco*-configured furanose precursors. A theobromine 6′-isonucleoside was shown to act as a dual AChE/BChE inhibitor at single-digit micromolar concentration range, with a *K*_i_ value for BChE being only ca. two-fold lower than that of galantamine hydrobromide. This finding, along with the rather short synthetic pathway for theobromine terminal isonucleosides, encouraging structural optimization, points to the interest of this type of scaffold in the search for stable and non-cytotoxic cholinesterase inhibitor lead compounds.

## Figures and Tables

**Table 1:** Results of the evaluation of the inhibitory activities of the compounds on cholinesterases.

	AChE		BChE	
Compound	% Inhibition ^a^	*K*_i_ (µM) [*K*_i_′ (µM)] (type of inhibition)	% Inhibition ^a^	*K*_i_ (µM) [*K*_i_′ (µM)] (type of inhibition)
Galantamine hydrobromide	96.7	0.2 ± 0.1 (competitive)	86.3	2.4 ± 0.0 (competitive)
Xylofuranos-5′-yl isonucleosides				
*Purine derivatives*				
**4**	16.3	n.d.	11.9	n.d.
**5**	25.3	n.d.	32.5	n.d.
*Uracil derivatives*				
**6**	22.5	n.d.	28.5	n.d.
**7**	17.8	n.d.	7.5	n.d.
**8**	26.1	n.d.	32.8	n.d.
**9**	40.3	17.5 ± 0.7 [103.0 ± 4.3] (mixed-type)	29.8	n.d.
*Theobromine derivatives*				
**3**	13.8	n.d.	11.7	n.d.
**10**	2.6	n.d.	22.9	n.d.
**13**-**α**	18.9	n.d.	35.0	n.d.
**18**	14.5	n.d.	19.6	n.d.
Glucopyranos-6′-yl theobromine isonucleoside				
**21**	66.5	3.1 ± 0.2 [>100] (mixed-type)	75.1	5.4 ± 0.3 [>60] (mixed-type)
Theobromine-containing isonucleotide analogs				
**12**	1.0	n.d.	4.6	n.d.
**15**	12.4	n.d.	35.9	n.d.
**22**	28.3	n.d.	52.6	n.d.

^a^ % Inhibition at 50 µM. AChE, acetylcholinesterase; BChE, butyrylcholinesterase.

## Supplementary Materials

The following are available online at https://www.mdpi.com/1424-8247/12/3/103/s1.
