# Supplementary material for: Synthesis and Biological Evaluation of Structurally Varied 5′-/6′-Isonucleosides and Theobromine-Containing N-Isonucleosidyl Derivatives
Source: Pharmaceuticals (Basel). 2019 Jul 2;12(3):103. doi: 10.3390/ph12030103 (PMC6790002; doi:10.3390/ph12030103)
Supplement: Supplementary file 1 [file pharmaceuticals-12-00103-s001.pdf]

Supplementary Materials

Synthesis and Biological Evaluation of Structurally Varied 5'-/6'-Isonucleosides and Theobromine-containing *N*-Isonucleosidyl Derivatives

Nuno M. Xavier [1,2,*], Eduardo C. de Sousa [1], Margarida P. Pereira [1,2], Anne Loesche [3], Immo Serbian [3], René Csuk [3] and M. Conceição Oliveira [4]

[1] Centro de Química e Bioquímica, Faculdade de Ciências, Universidade de Lisboa, Ed. C8, 5º Piso, Campo Grande, 1749-016 Lisboa, Portugal
[2] Centro de Química Estrutural, Faculdade de Ciências, Universidade de Lisboa, Ed. C8, 5º Piso, Campo Grande, 1749-016 Lisboa, Portugal
[3] Bereich Organische Chemie, Martin-Luther-Universität Halle-Wittenberg, Kurt-Mothes-Str. 2, D-06120 Halle (Saale), Germany
[4] Centro de Química Estrutural, Instituto Superior Técnico, Universidade de Lisboa, Av. Rovisco Pais, 1049-001 Lisboa, Portugal
* Correspondence: nmxavier@fc.ul.pt; Tel.: +351 217500853

Table of Contents

1. ^{1}H NMR and ^{13}C NMR Spectra for Compounds **3-12**, **13-α**, **15-22** **S2 – S20**

2. Enzymatic Studies **S21**

3. Best Docking Poses for Compounds **9**, **21-α** and **21-β** into BChE **S22**

1. ^{1}H NMR and ^{13}C NMR Spectra for Compounds 3-12, 13-α, 15-22

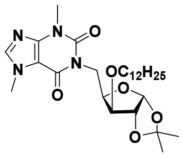

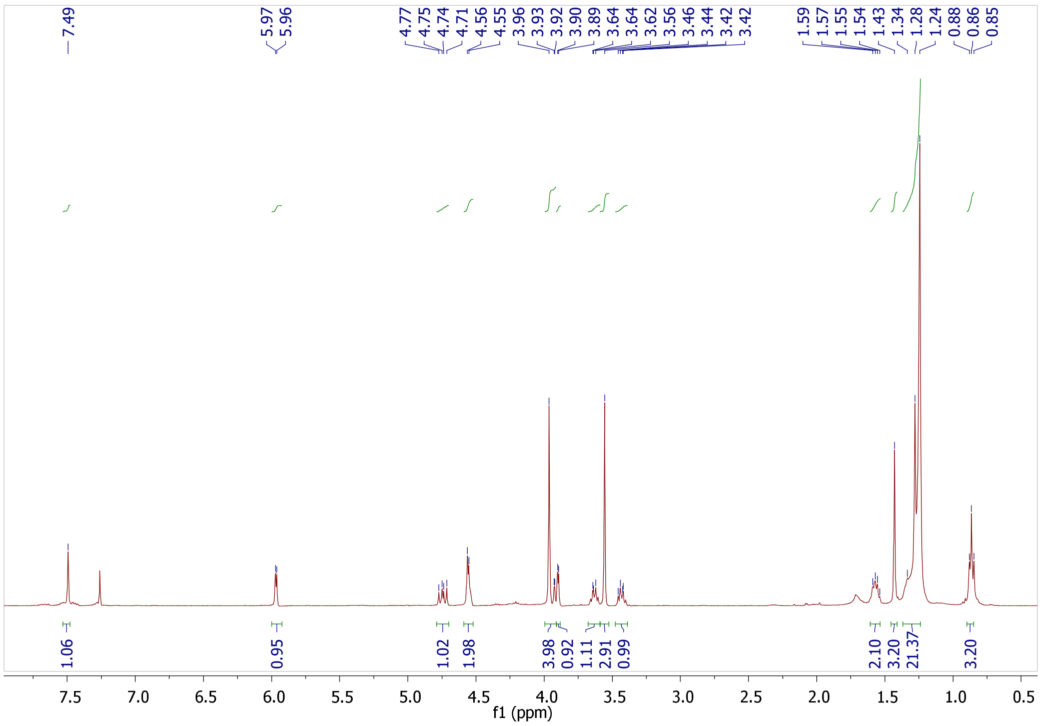

Figure S1 A. ^{1}H NMR Spectrum of compound **3**.

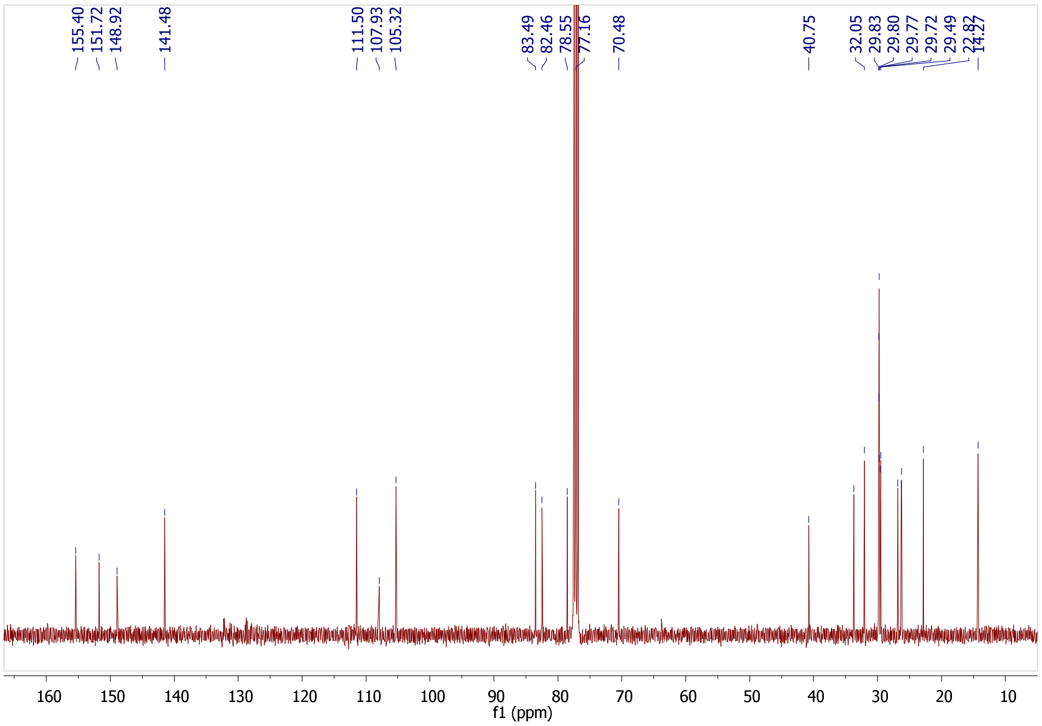

Figure S1 B. ^{13}C NMR Spectrum of compound **3**.

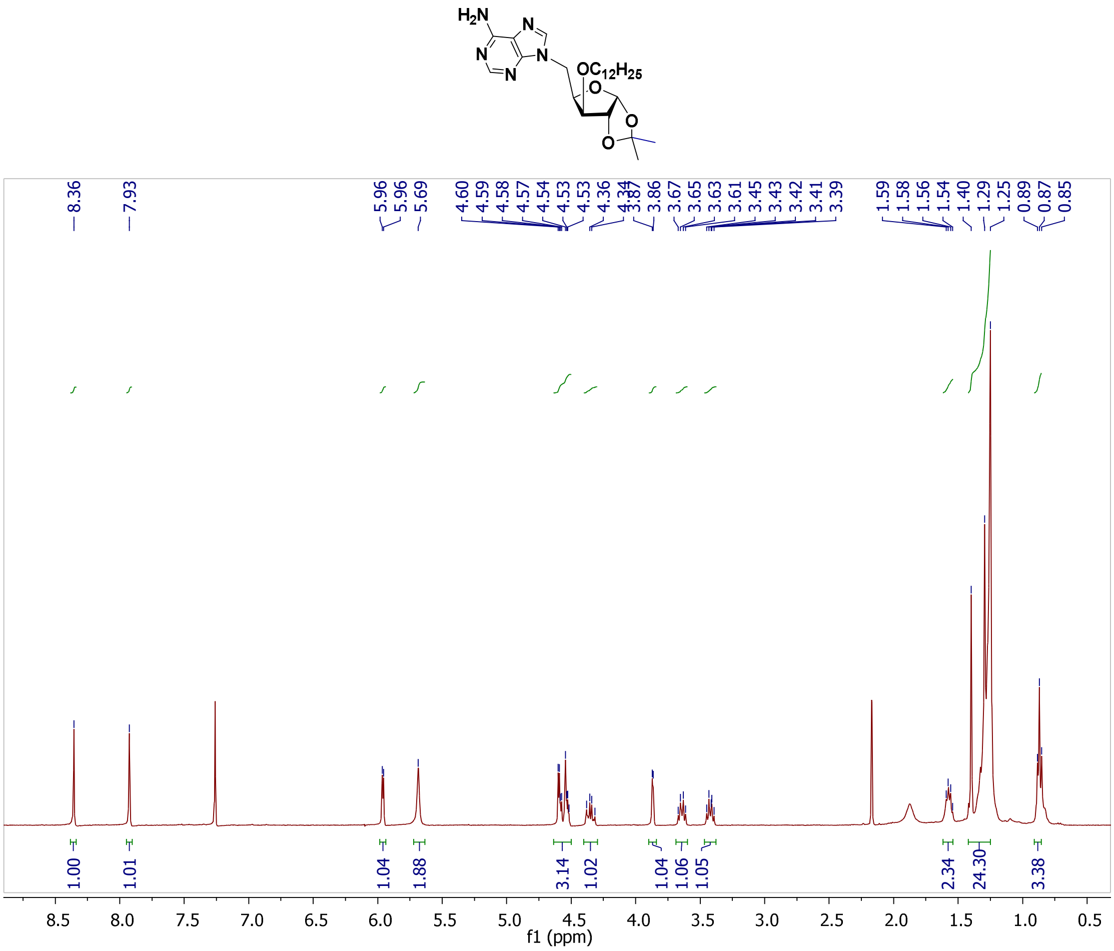

Figure S2 A. ^{1}H NMR Spectrum of compound **4**.

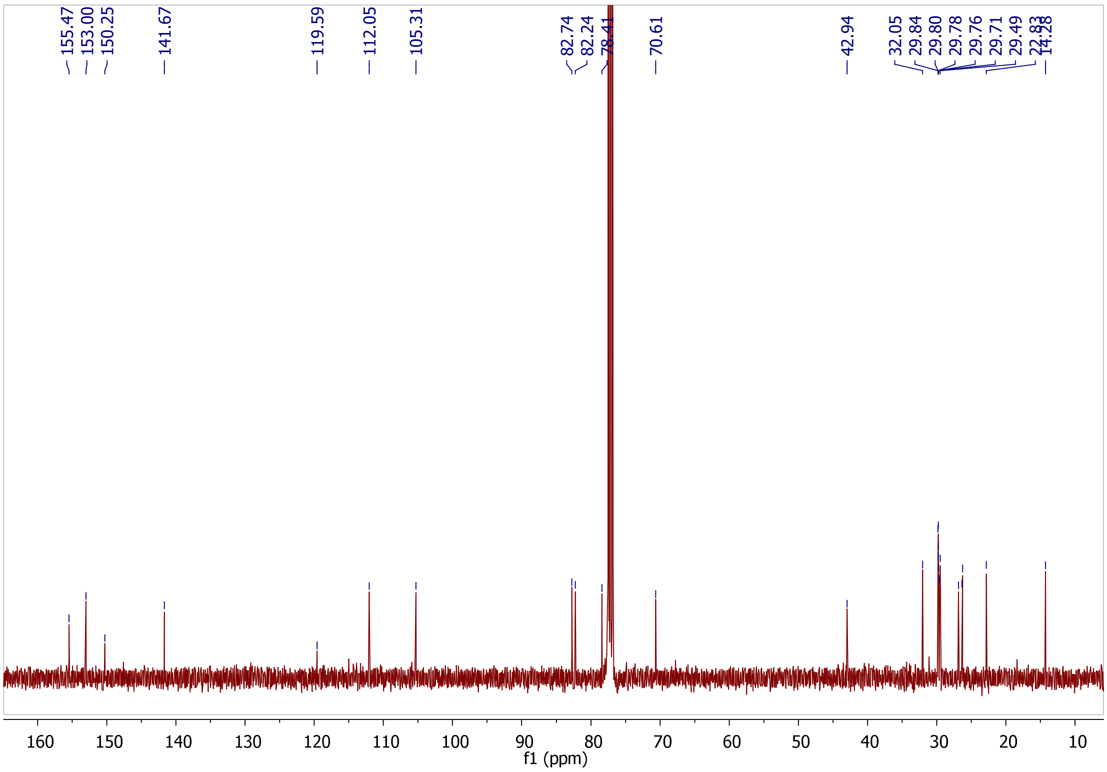

Figure S2 B. ^{13}C NMR Spectrum of compound **4**.

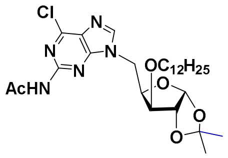

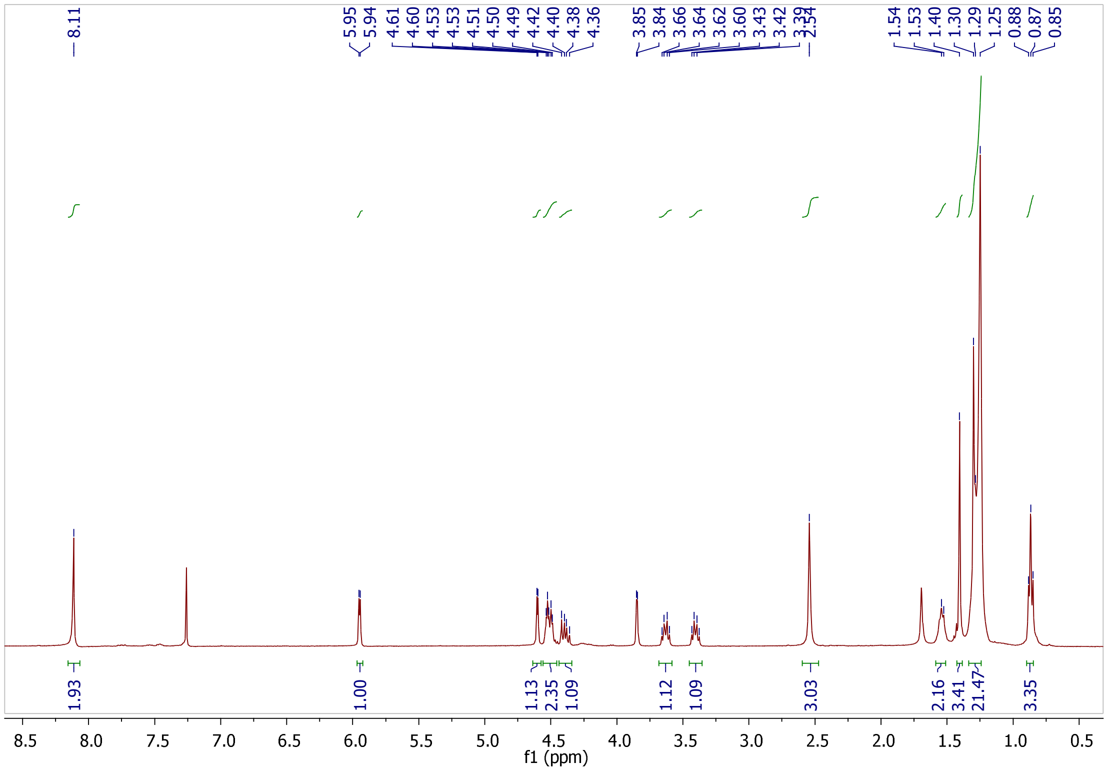

Figure S3 A. ^{1}H NMR Spectrum of compound **5**.

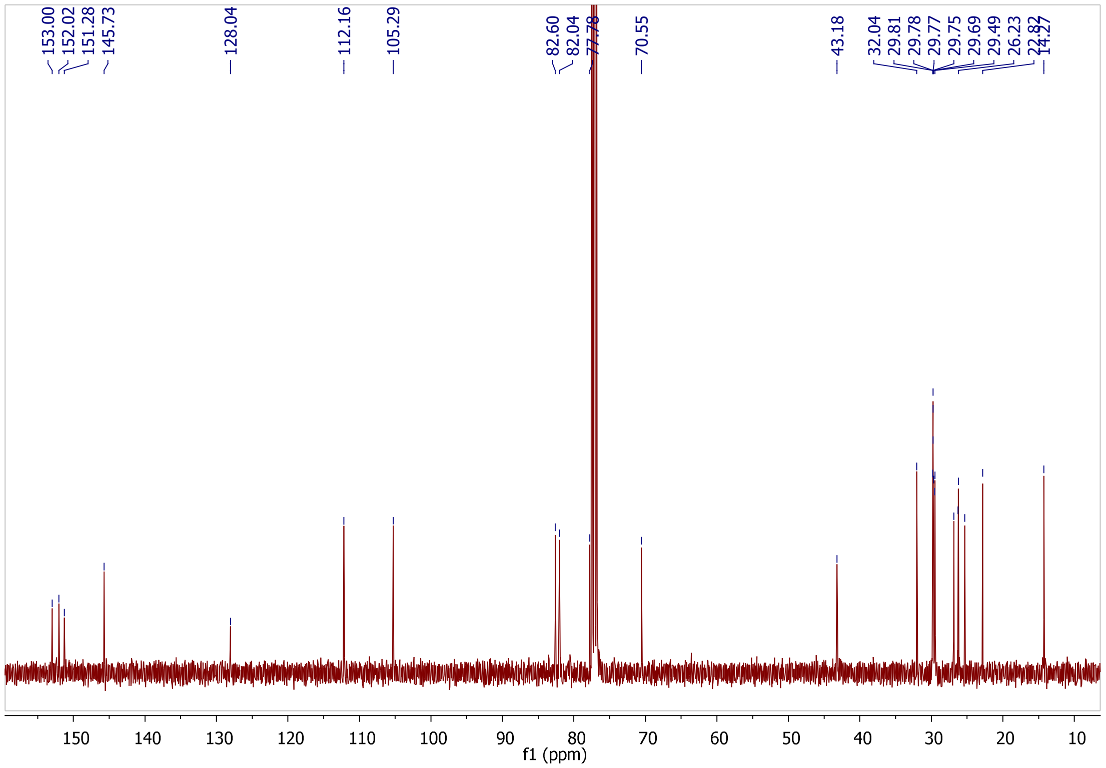

Figure S3 B. ^{13}C NMR Spectrum of compound **5**.

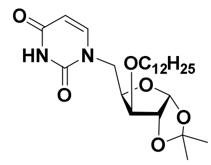

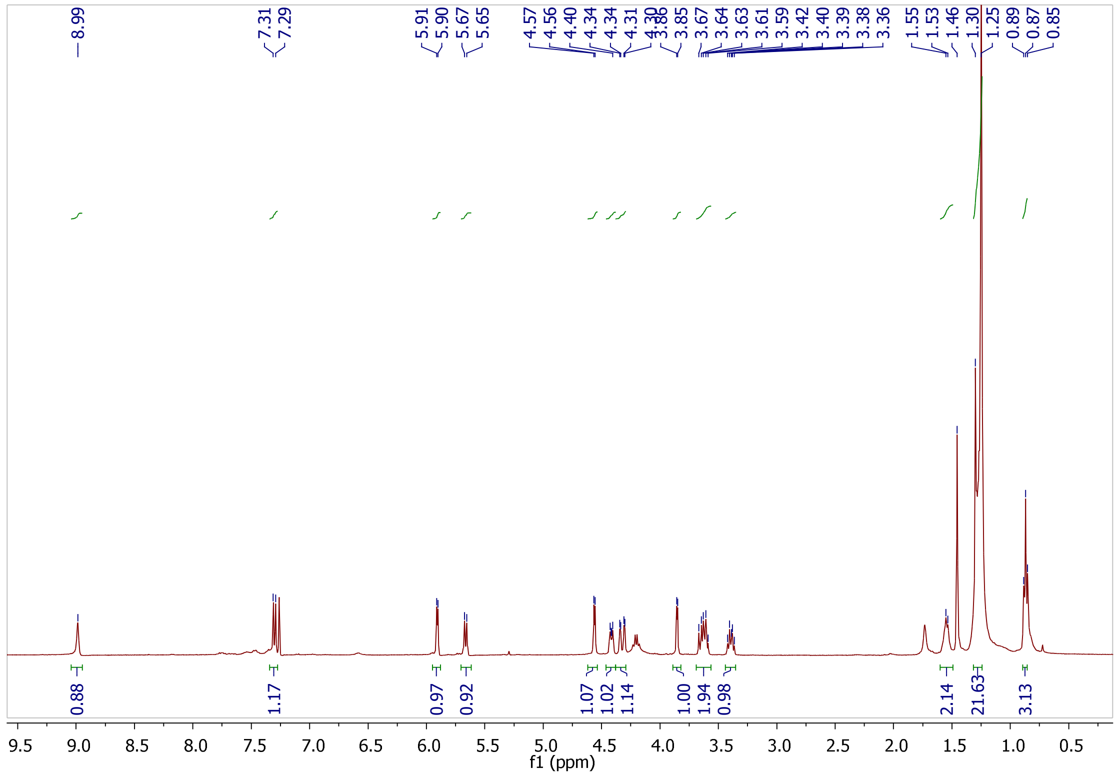

Figure S4 A. ^{1}H NMR Spectrum of compound **6**.

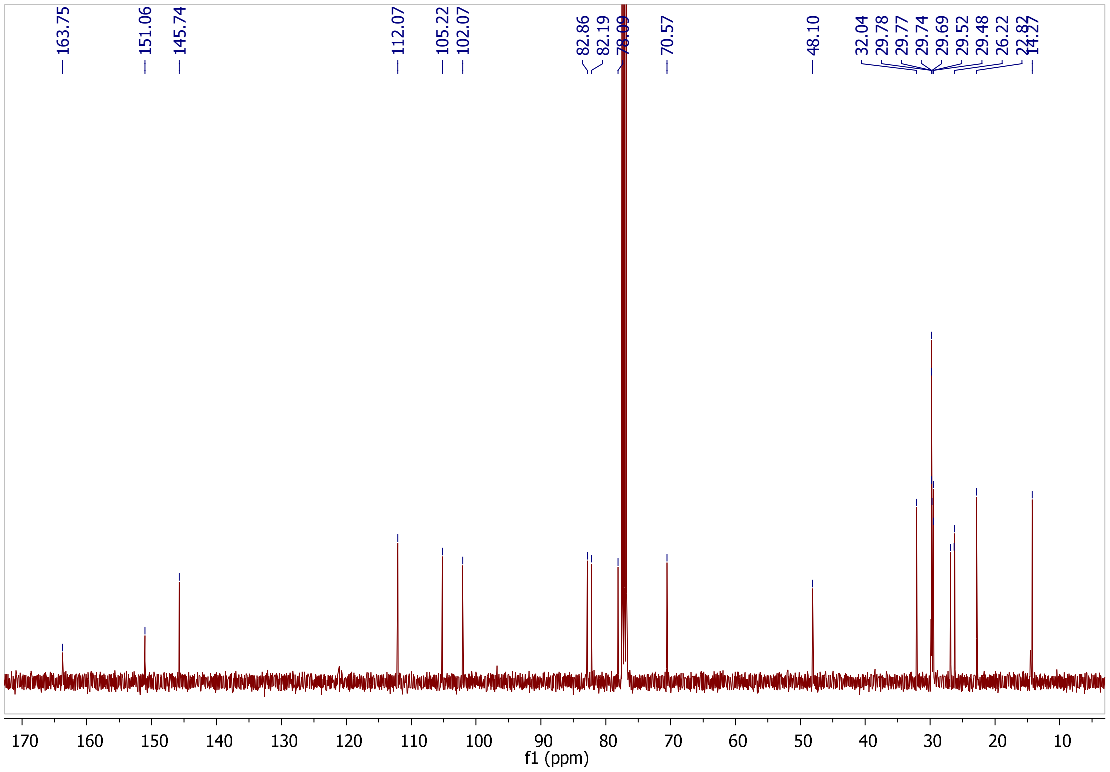

Figure S4 B. ^{13}C NMR Spectrum of compound **6**.

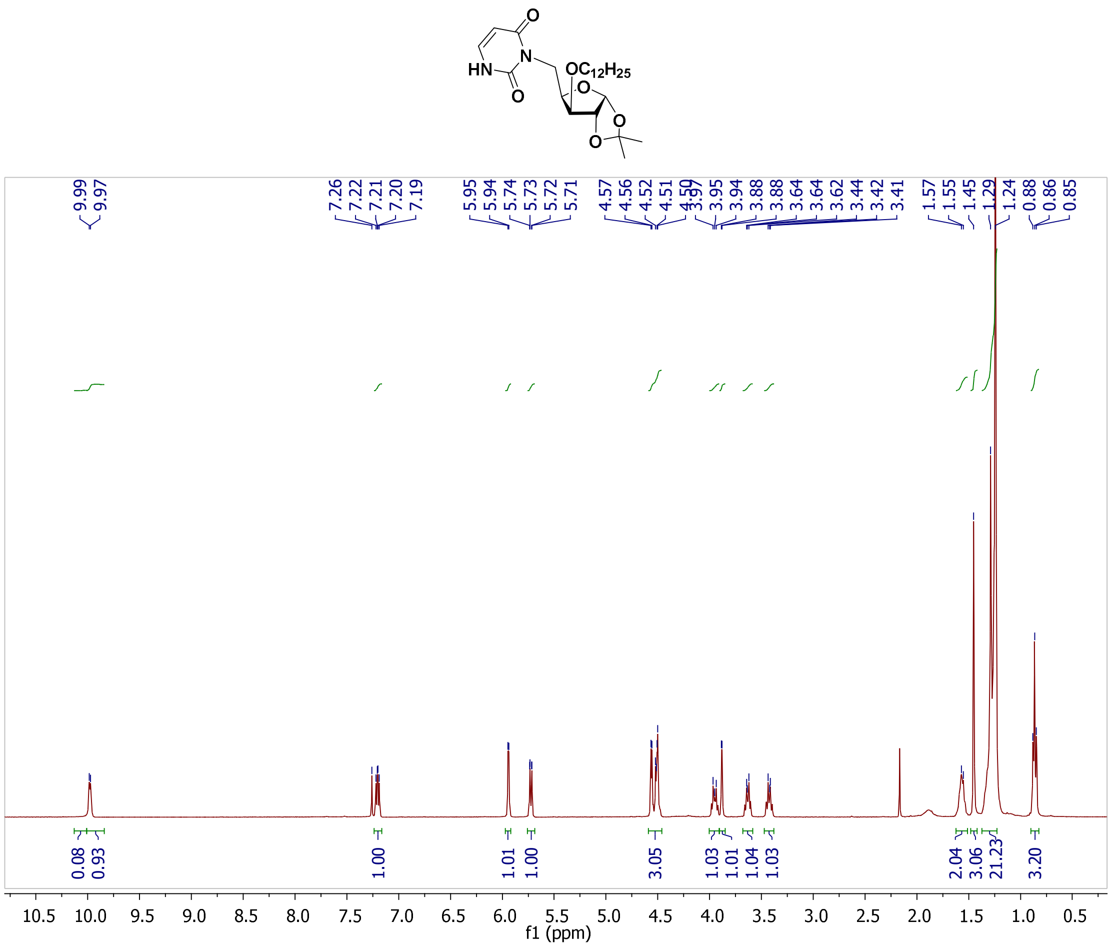

Figure S5 A. ^{1}H NMR Spectrum of compound **7**.

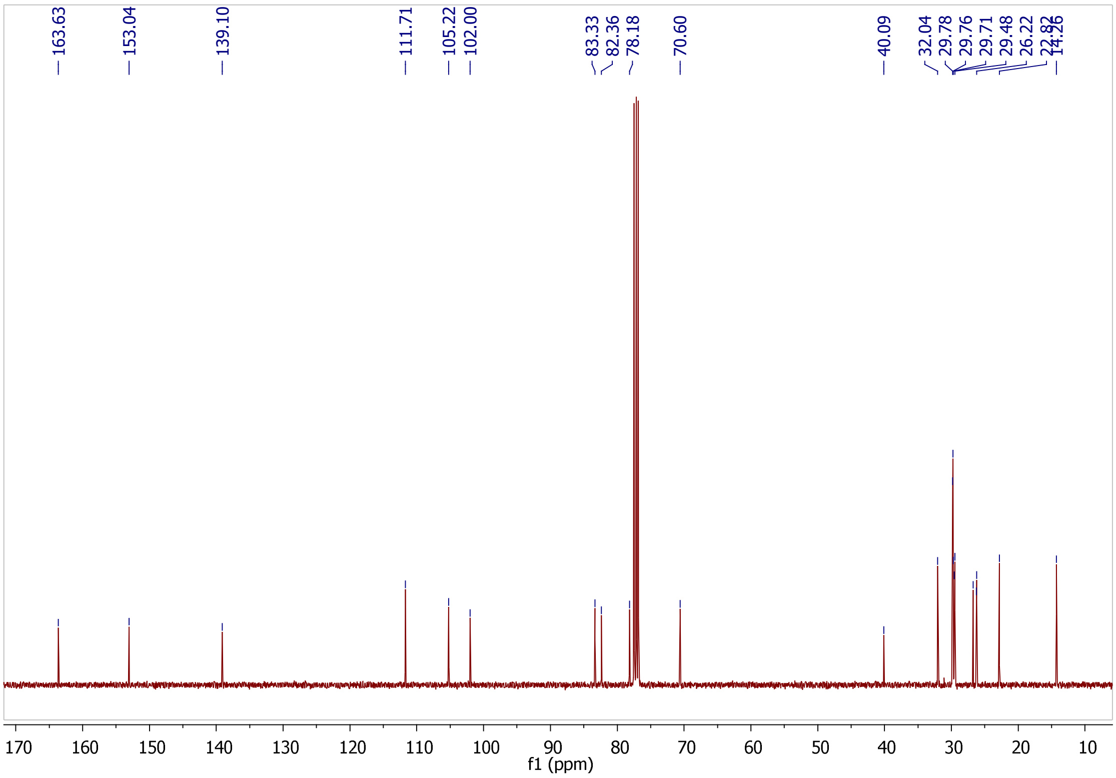

Figure S5 B. ^{13}C NMR Spectrum of compound **7**.

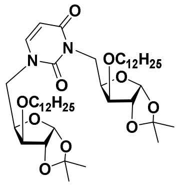

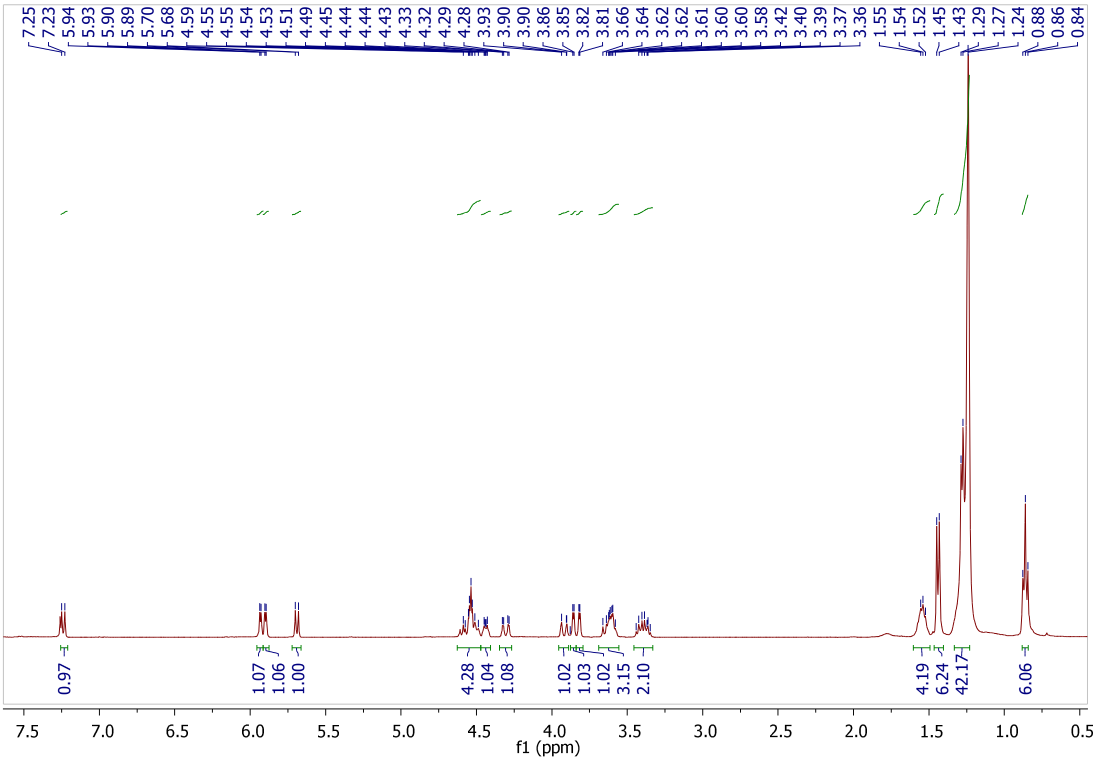

Figure S6 A. ^{1}H NMR Spectrum of compound **8**.

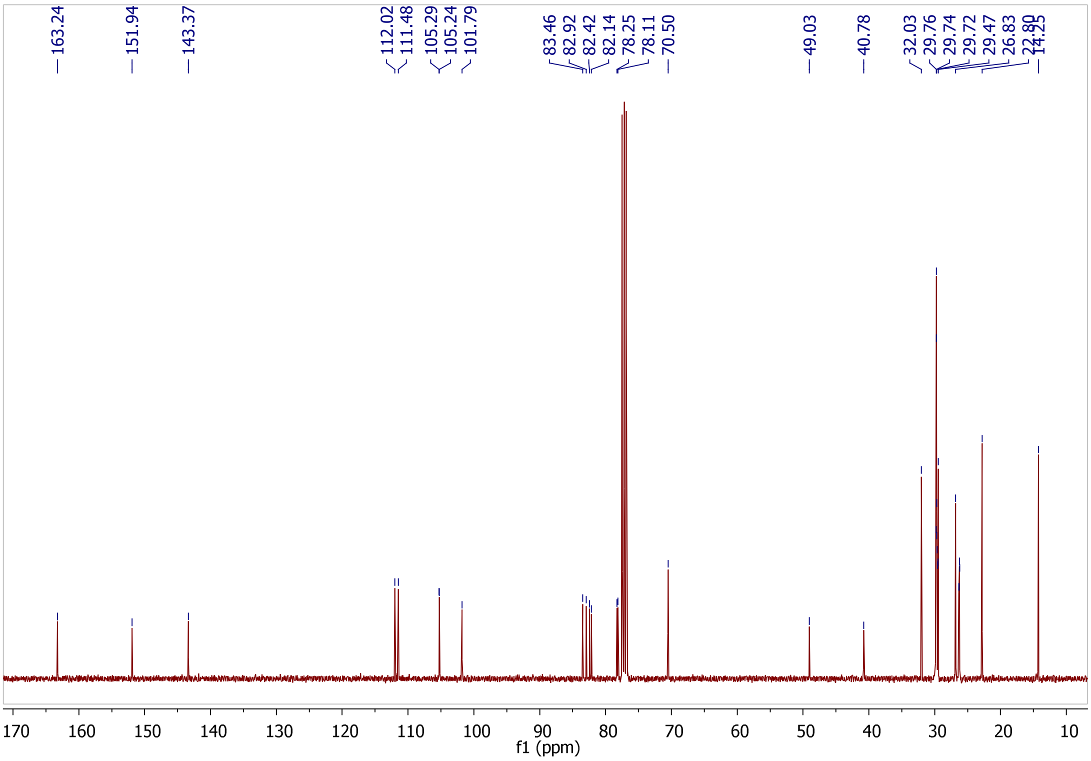

Figure S6 B. ^{13}C NMR Spectrum of compound **8**.

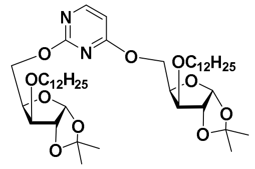

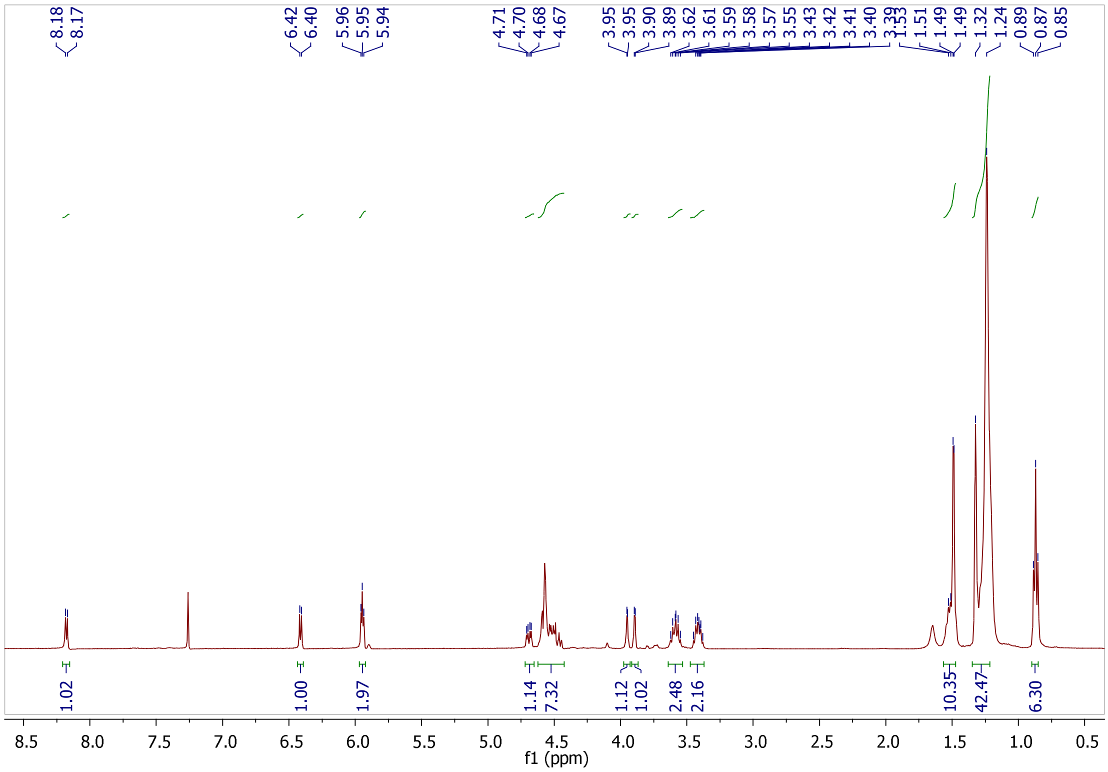

Figure S7 A. ¹H NMR Spectrum of compound **9**.

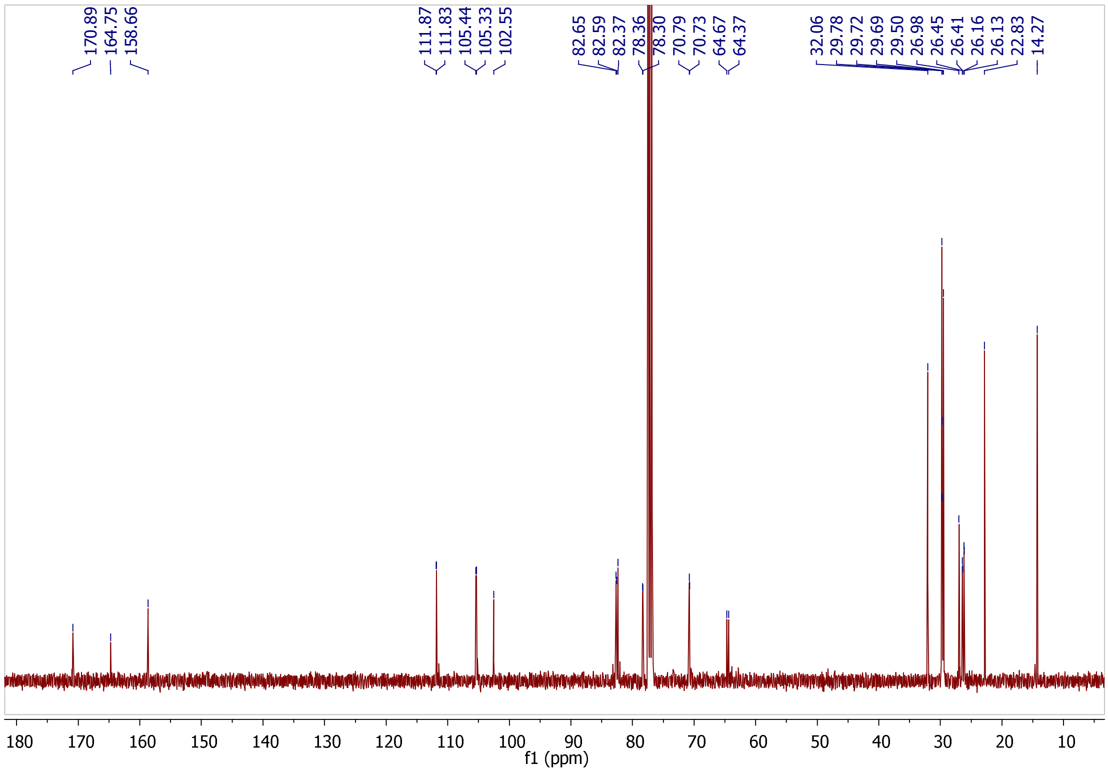

Figure S7 B. ¹³C NMR Spectrum of compound **9**.

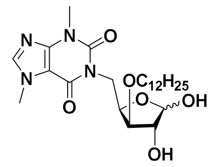

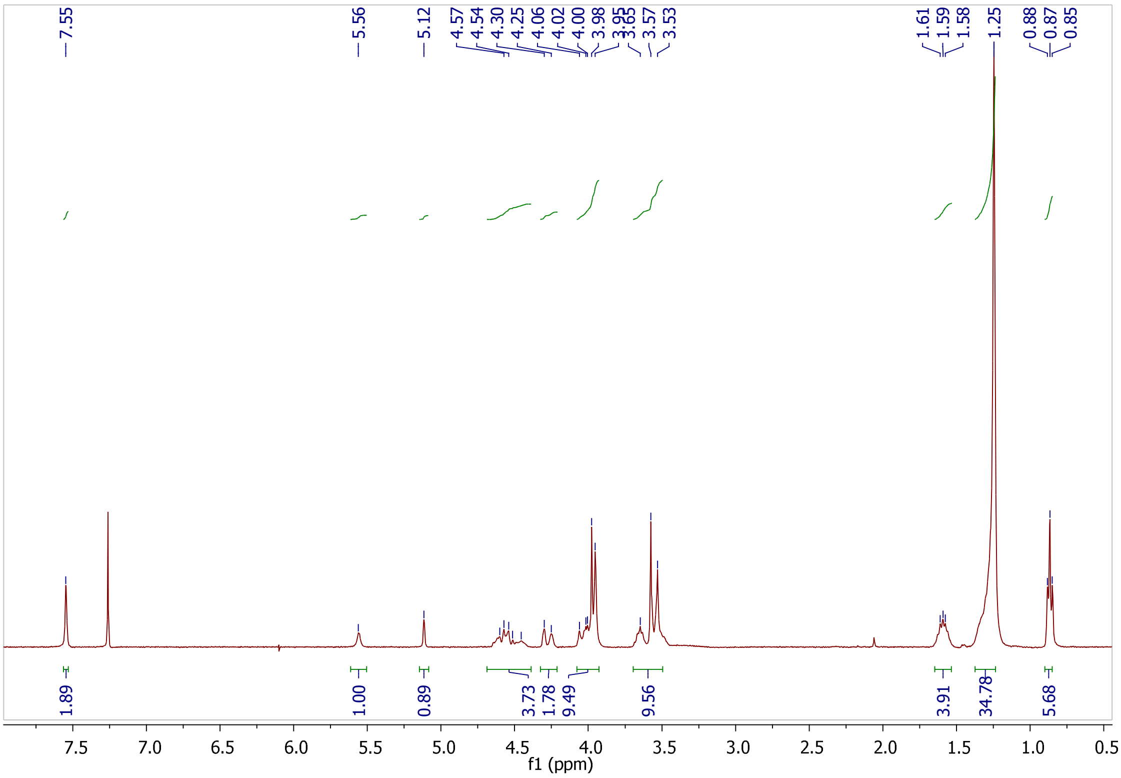

Figure S8 A. ^{1}H NMR Spectrum of compound **10**.

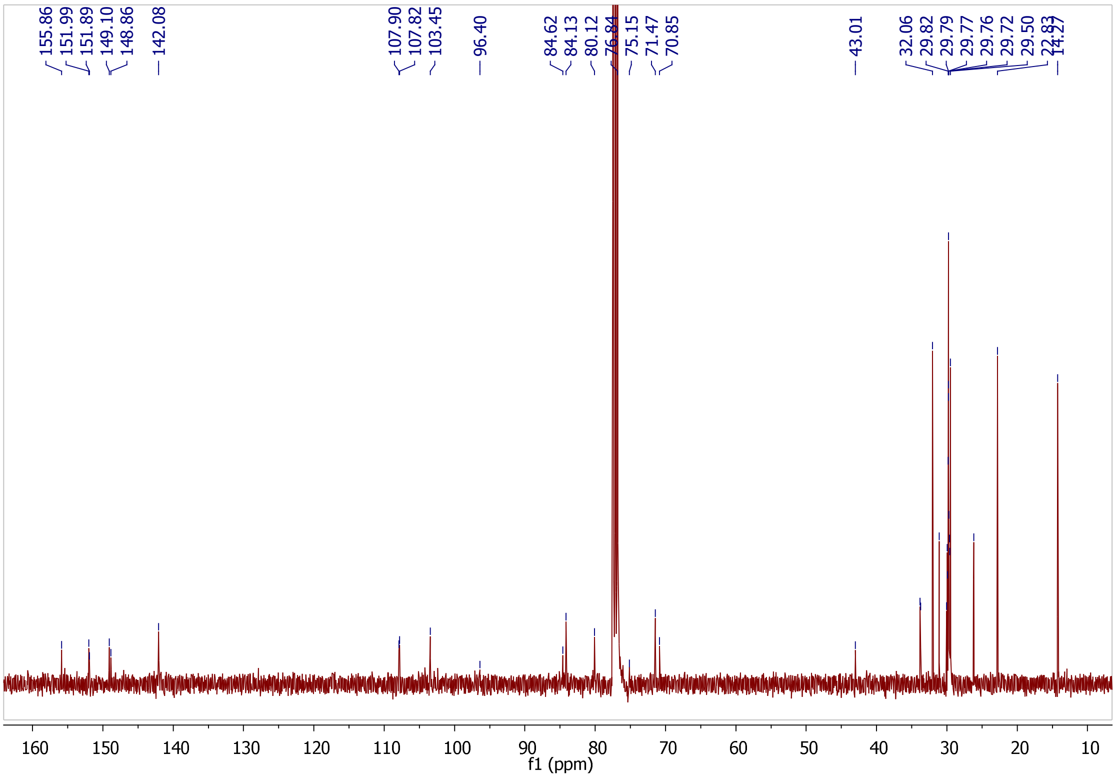

Figure S8 B. ^{13}C NMR Spectrum of compound **10**.

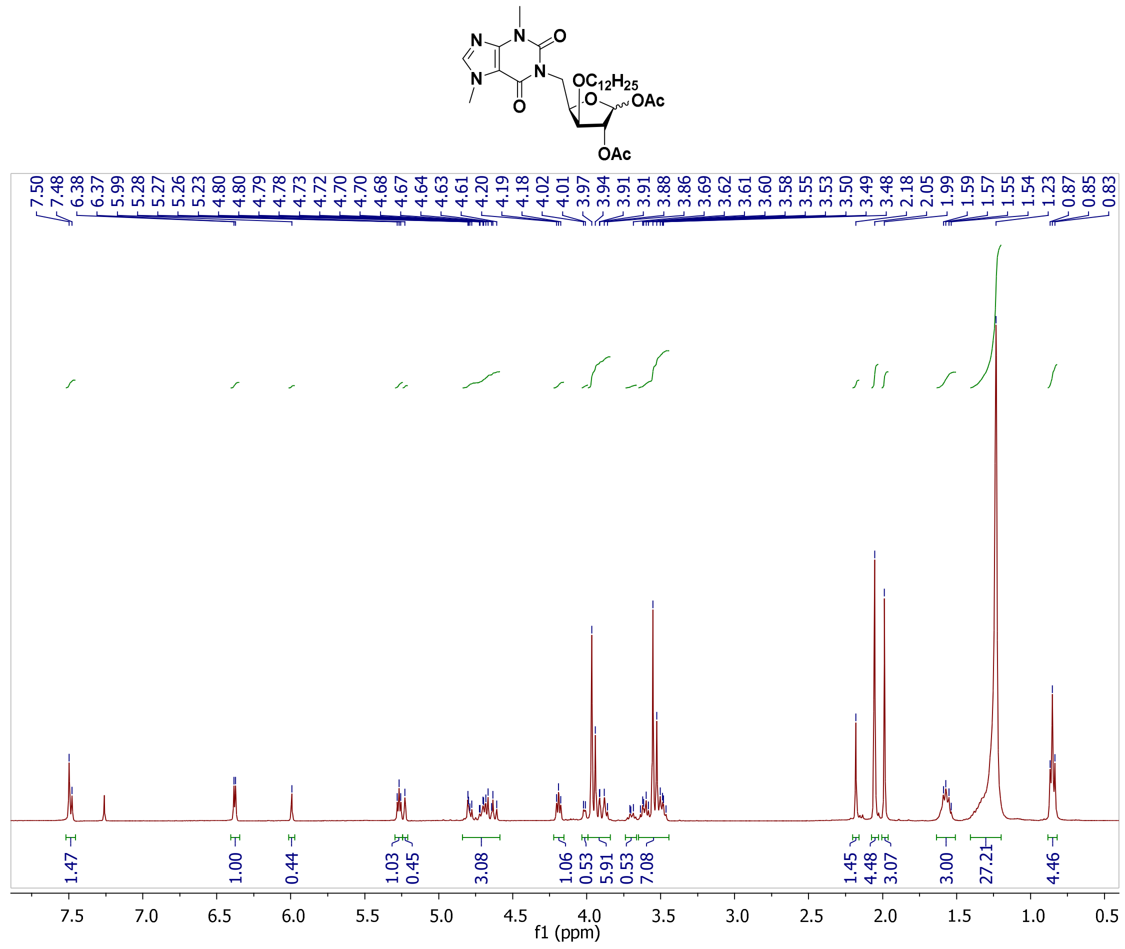

Figure S9 A. ¹H NMR Spectrum of compound **11**.

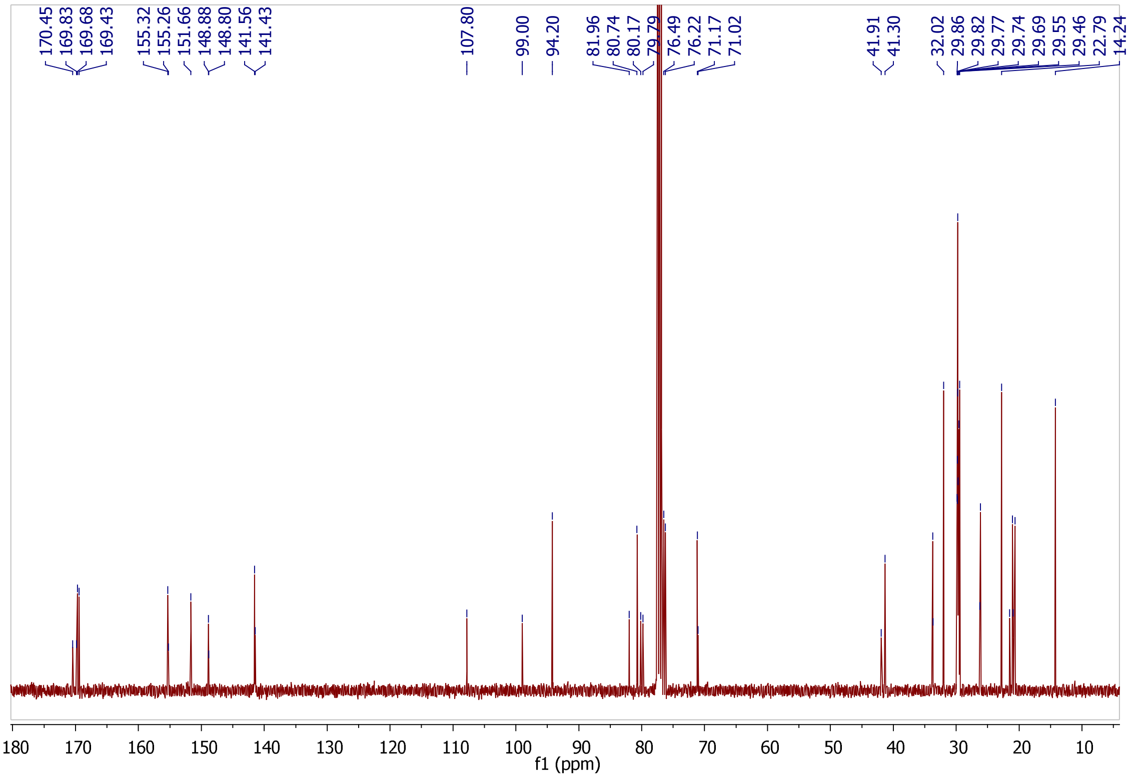

Figure S9 B. ¹³C NMR Spectrum of compound **11**.

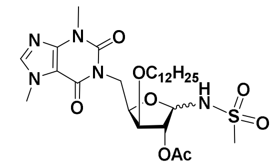

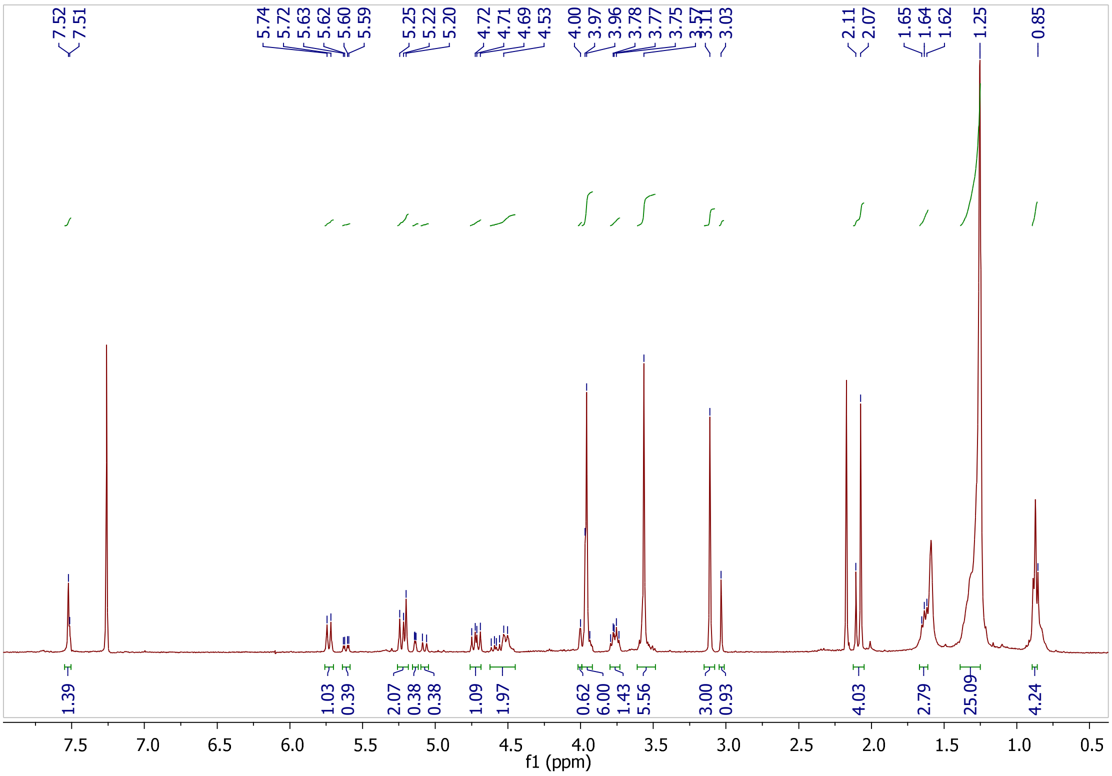

Figure S10 A. ^{1}H NMR Spectrum of compound **12**.

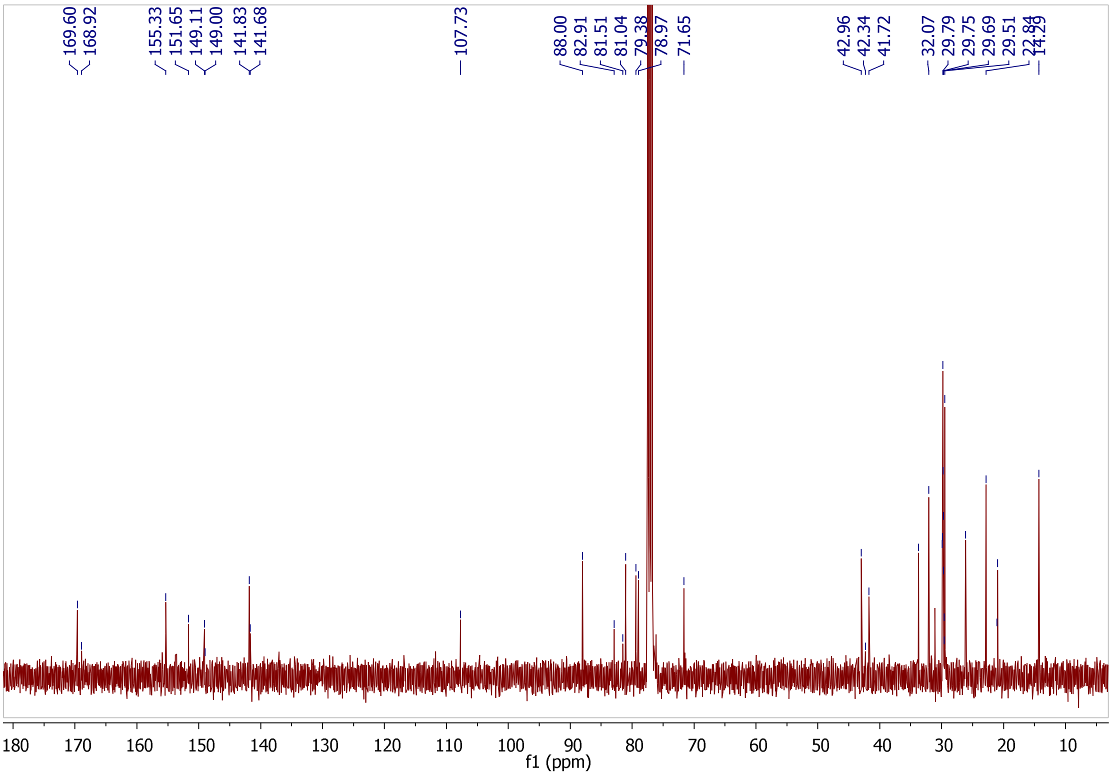

Figure S10 B. ^{13}C NMR Spectrum of compound **12**.

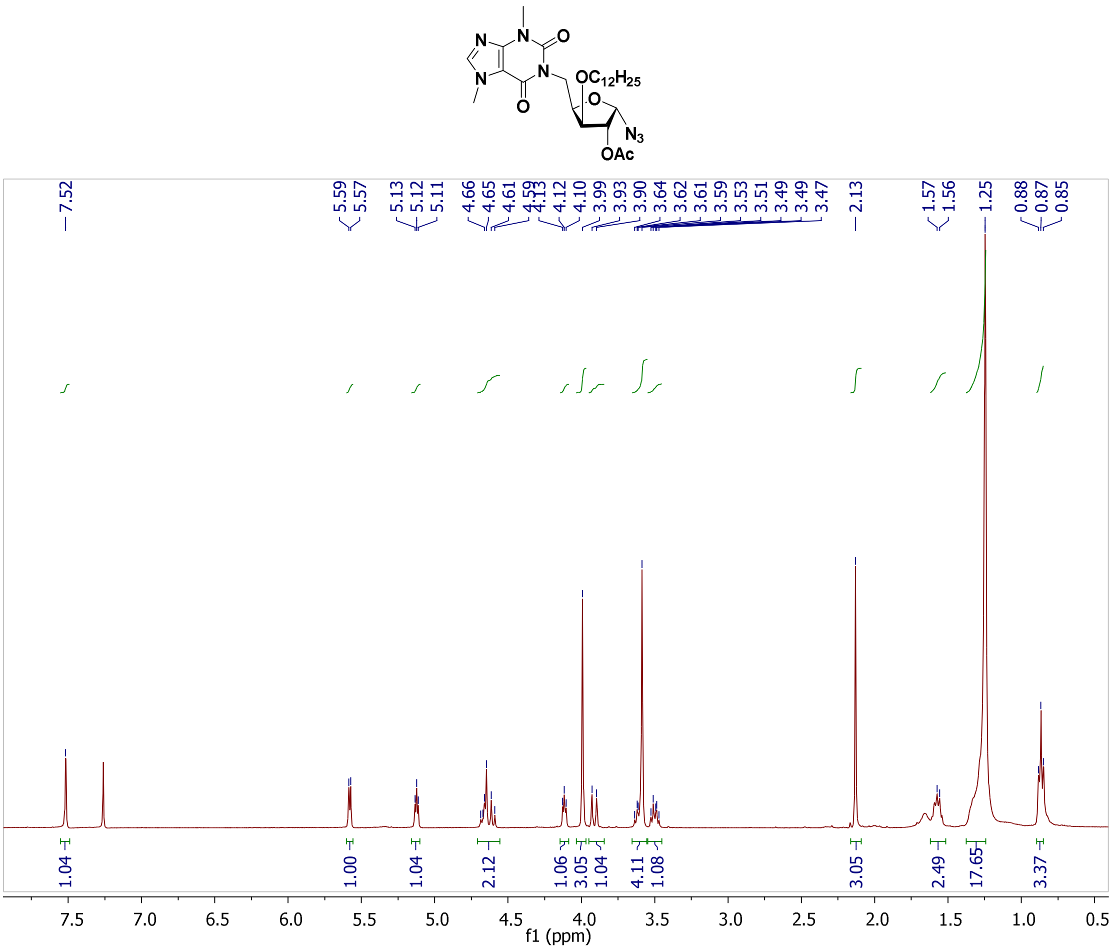

Figure S11 A. ^{1}H NMR Spectrum of compound **13-α**.

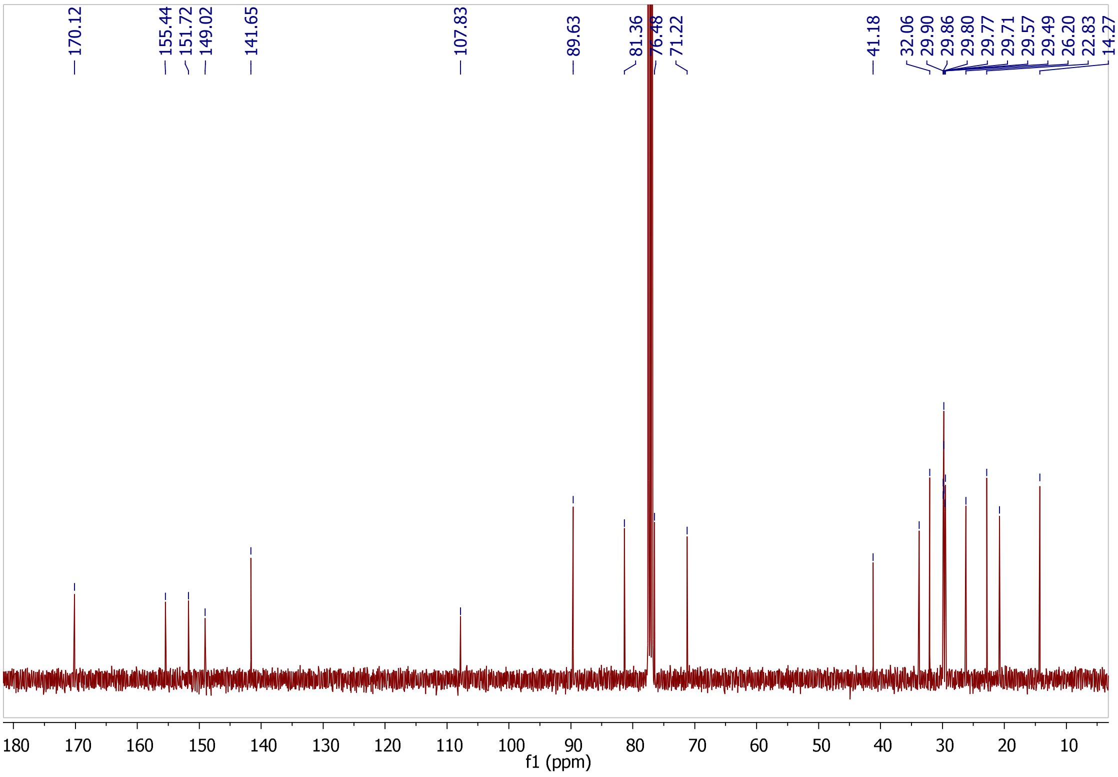

Figure S11 B. ^{13}C NMR Spectrum of compound **13-α**.

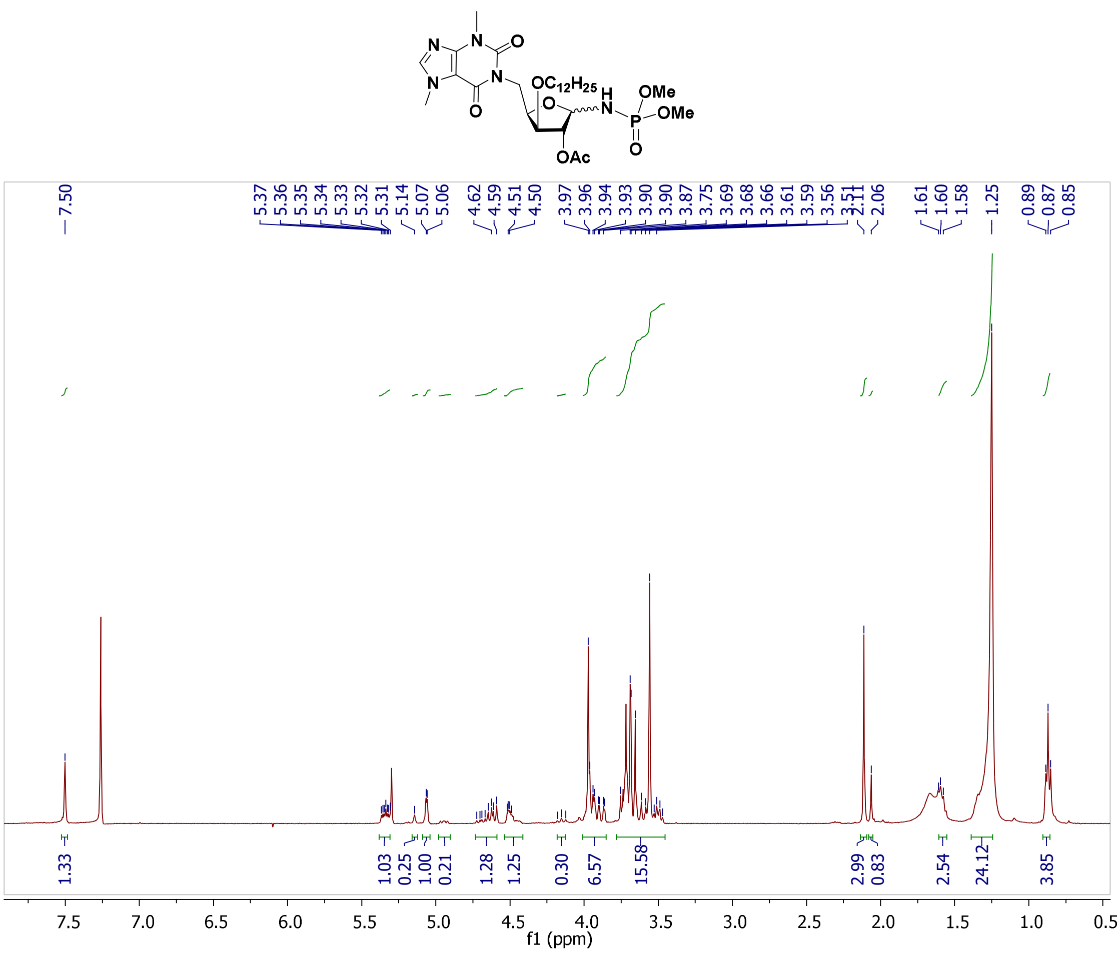

Figure S12 A. ^{1}H NMR Spectrum of compound **15**.

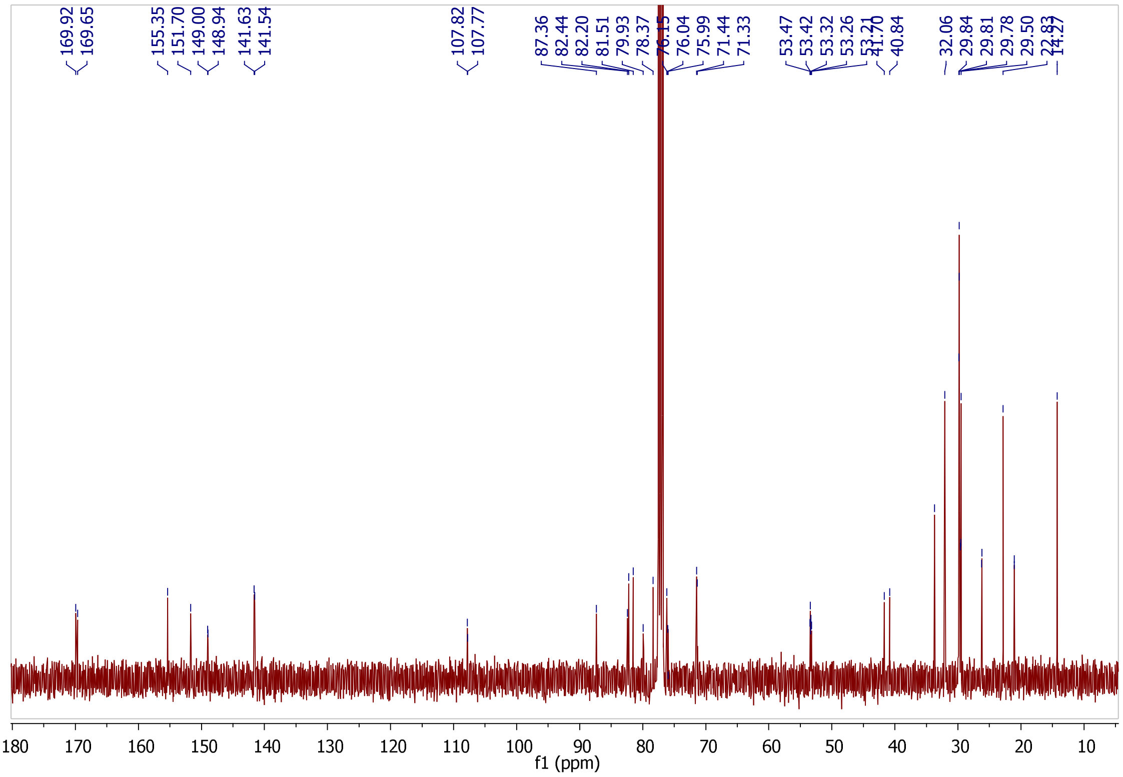

Figure S12 B. ^{13}C NMR Spectrum of compound **15**.

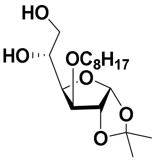

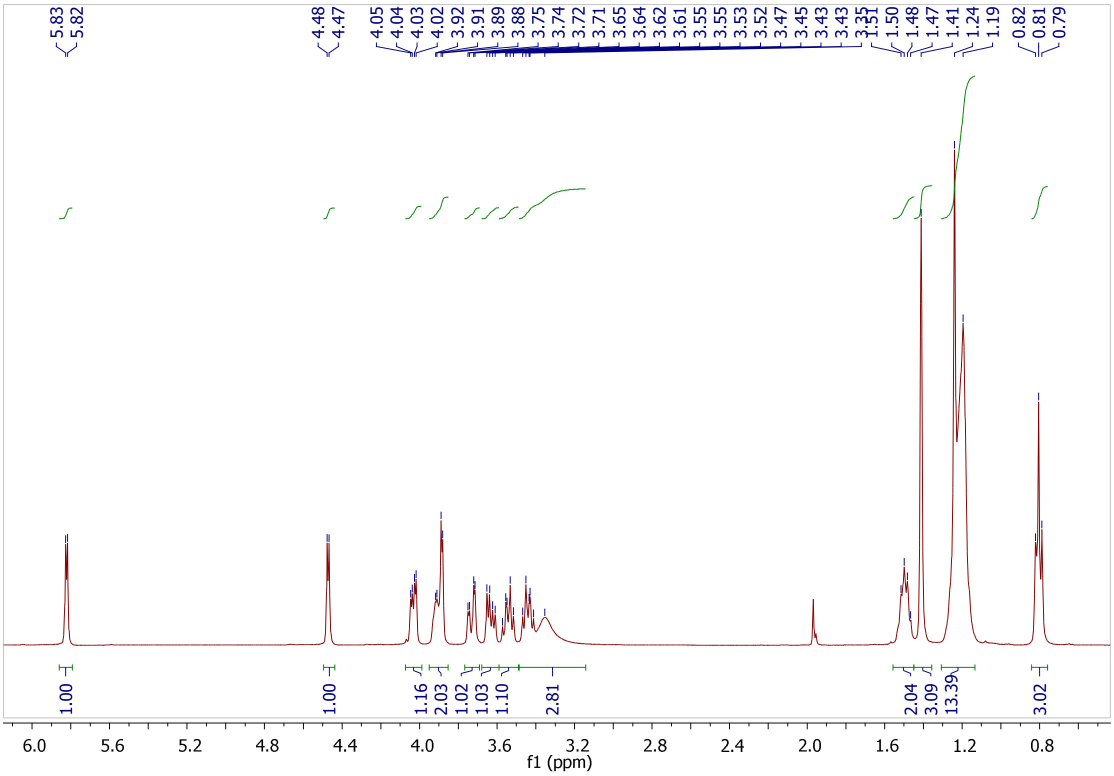

Figure S13 A. ^{1}H NMR Spectrum of compound **16**.

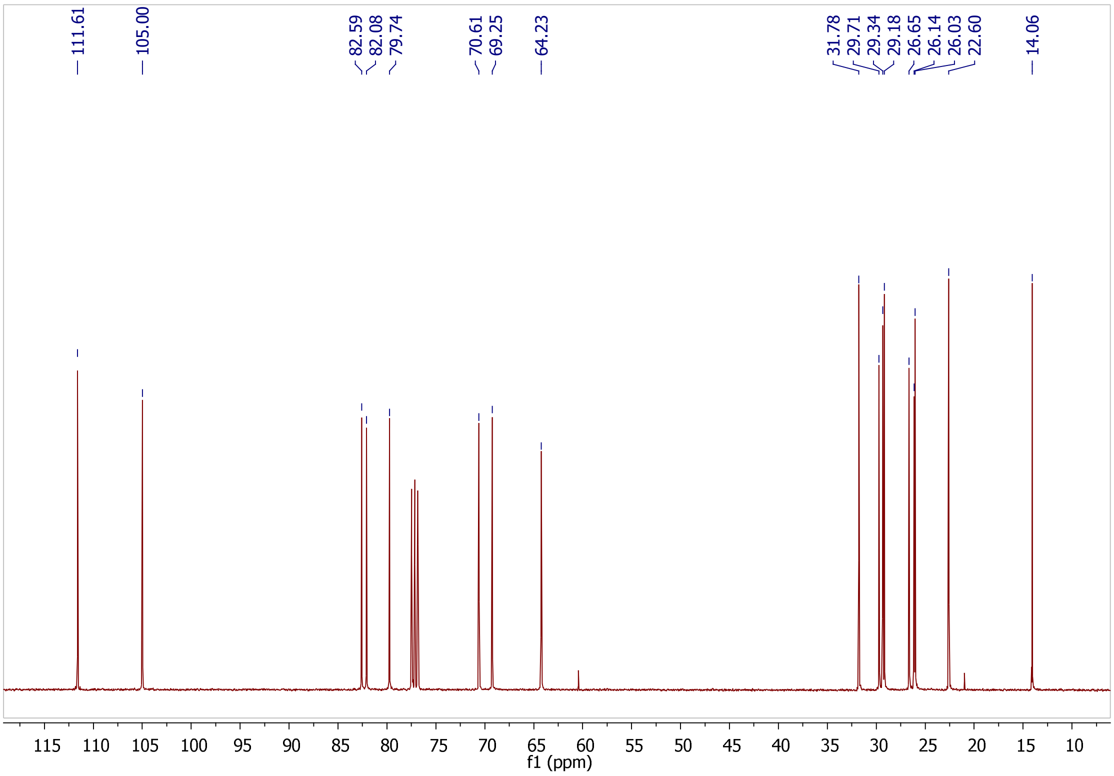

Figure S13 B. ^{13}C NMR Spectrum of compound **16**.

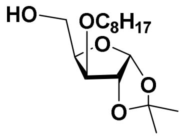

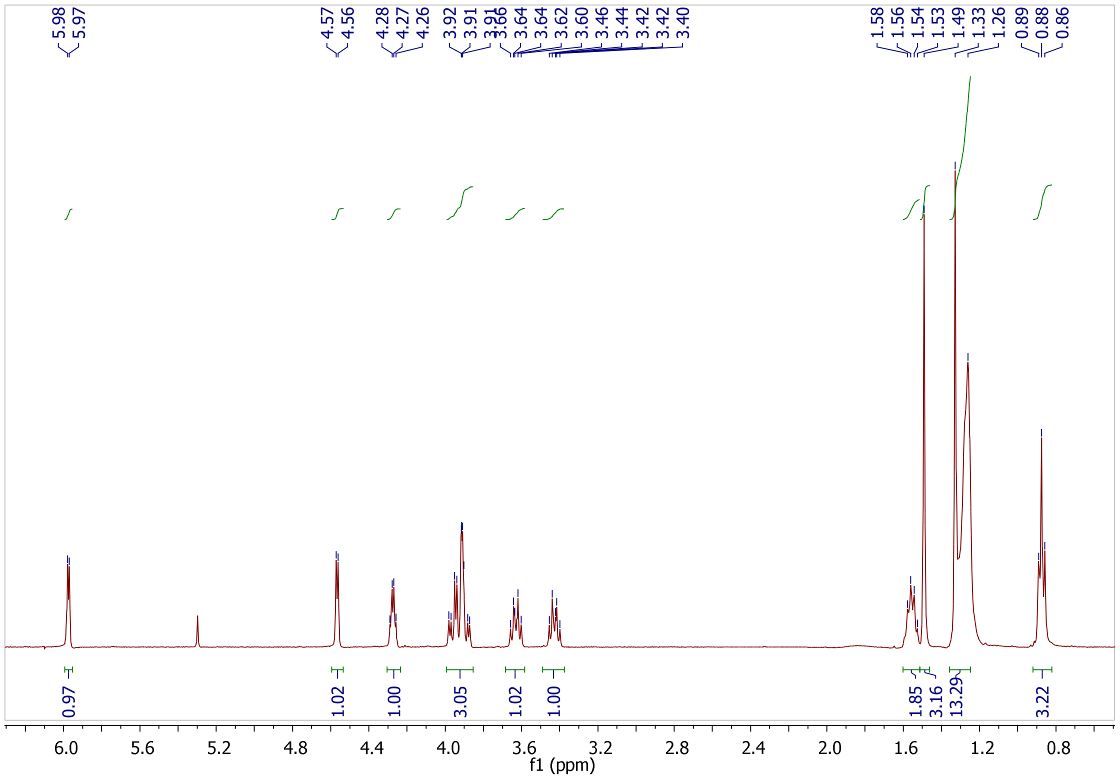

Figure S14 A. ¹H NMR Spectrum of compound **17**.

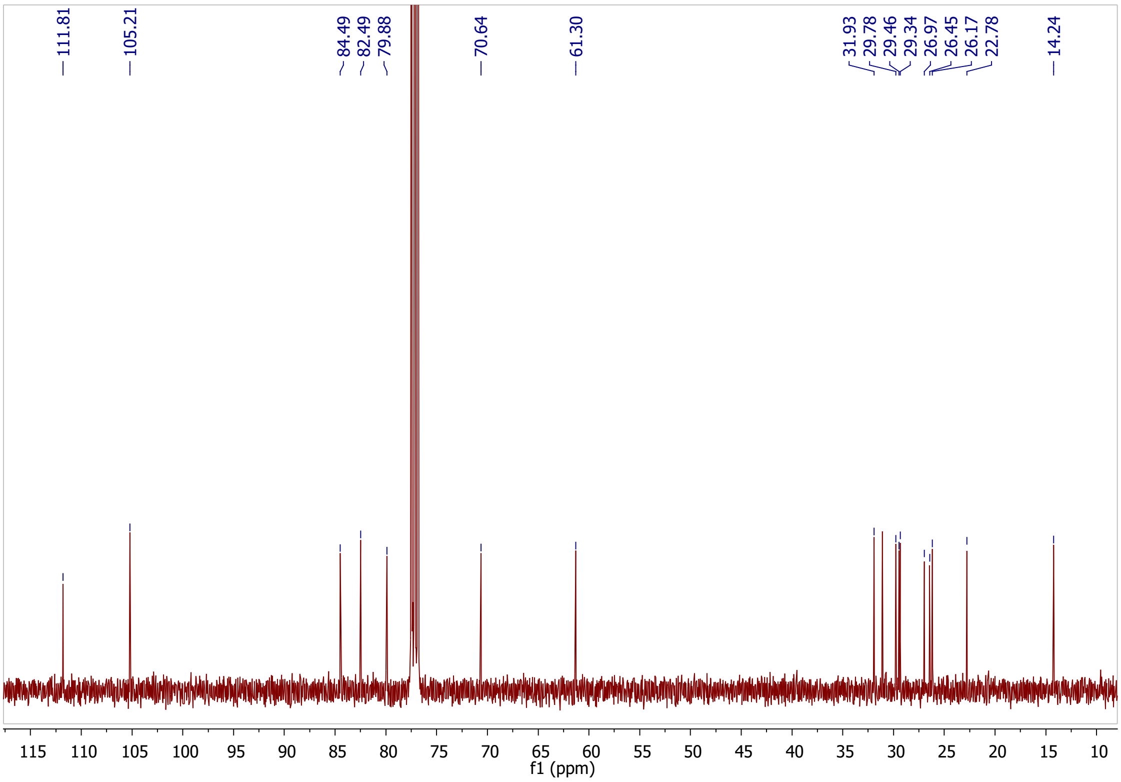

Figure S14 B. ¹³C NMR Spectrum of compound **17**.

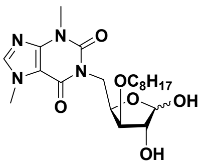

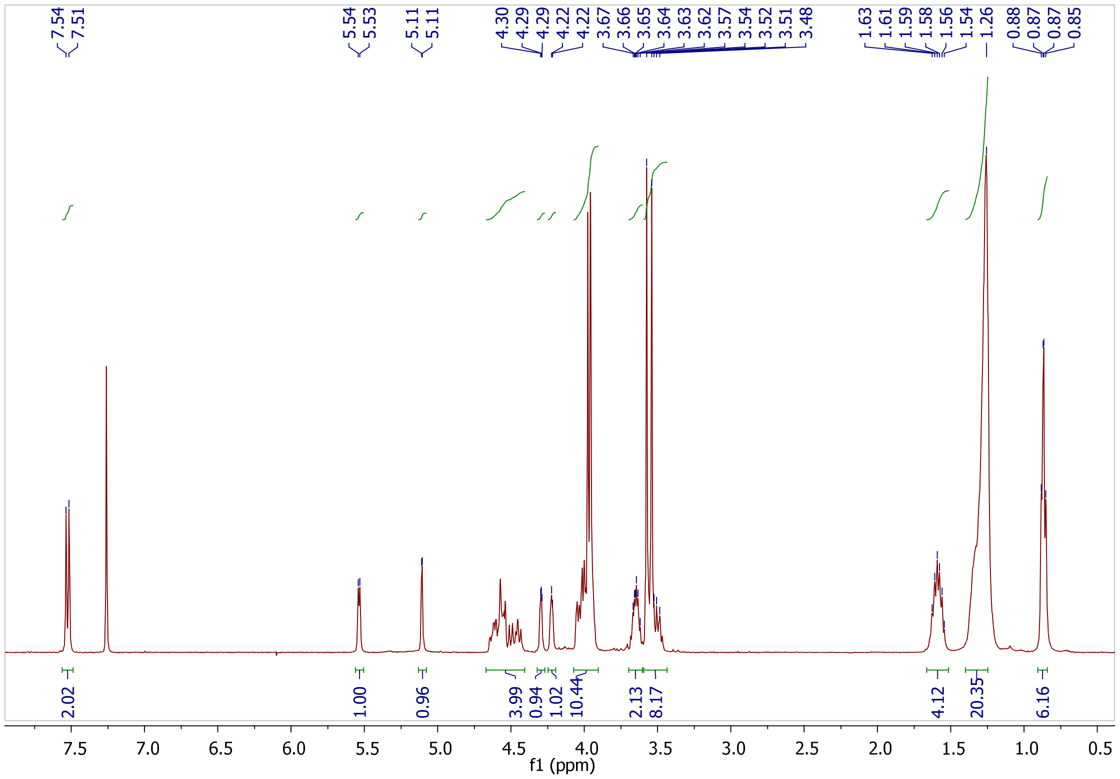

Figure S15 A. ^{1}H NMR Spectrum of compound **18**.

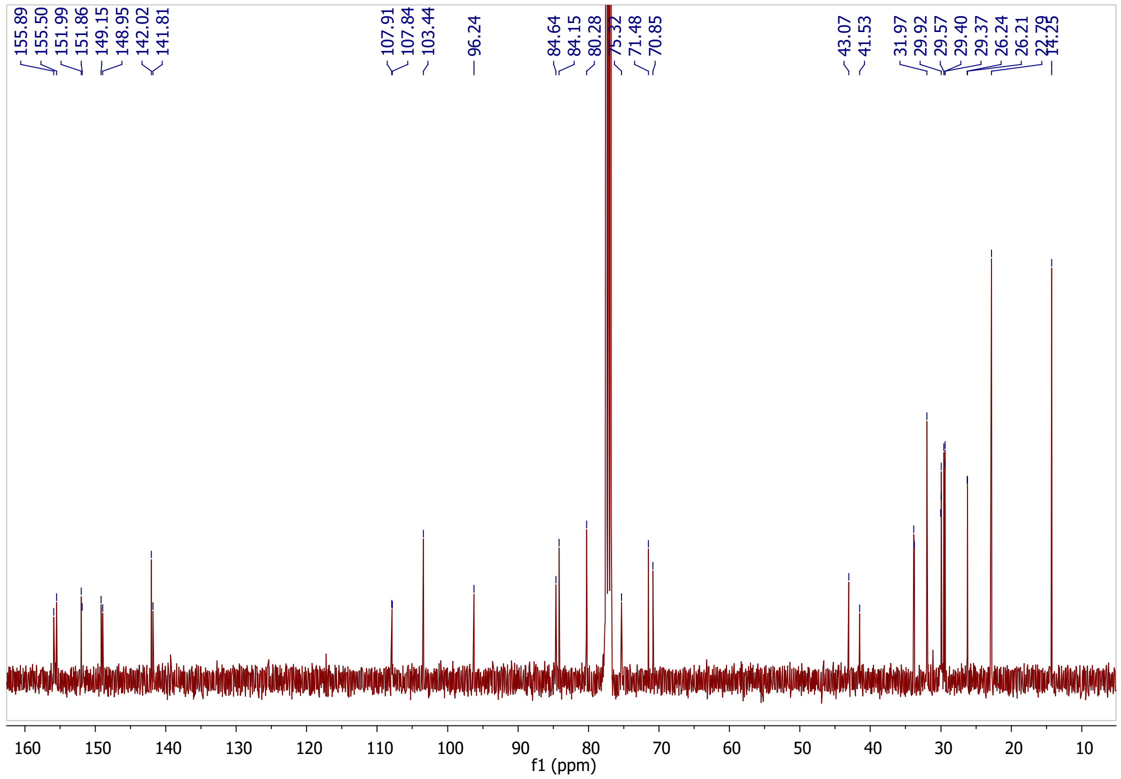

Figure S15 B. ^{13}C NMR Spectrum of compound **18**.

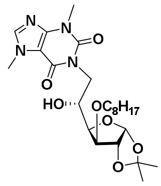

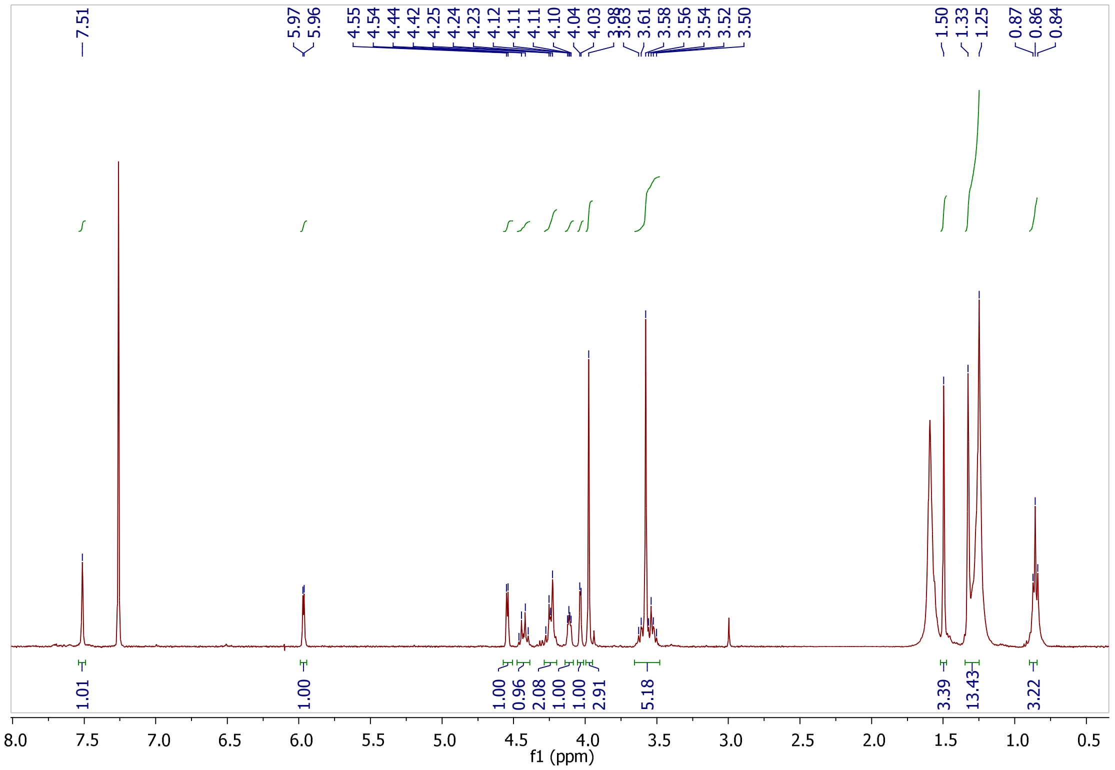

Figure S16 A. ^{1}H NMR Spectrum of compound **19**.

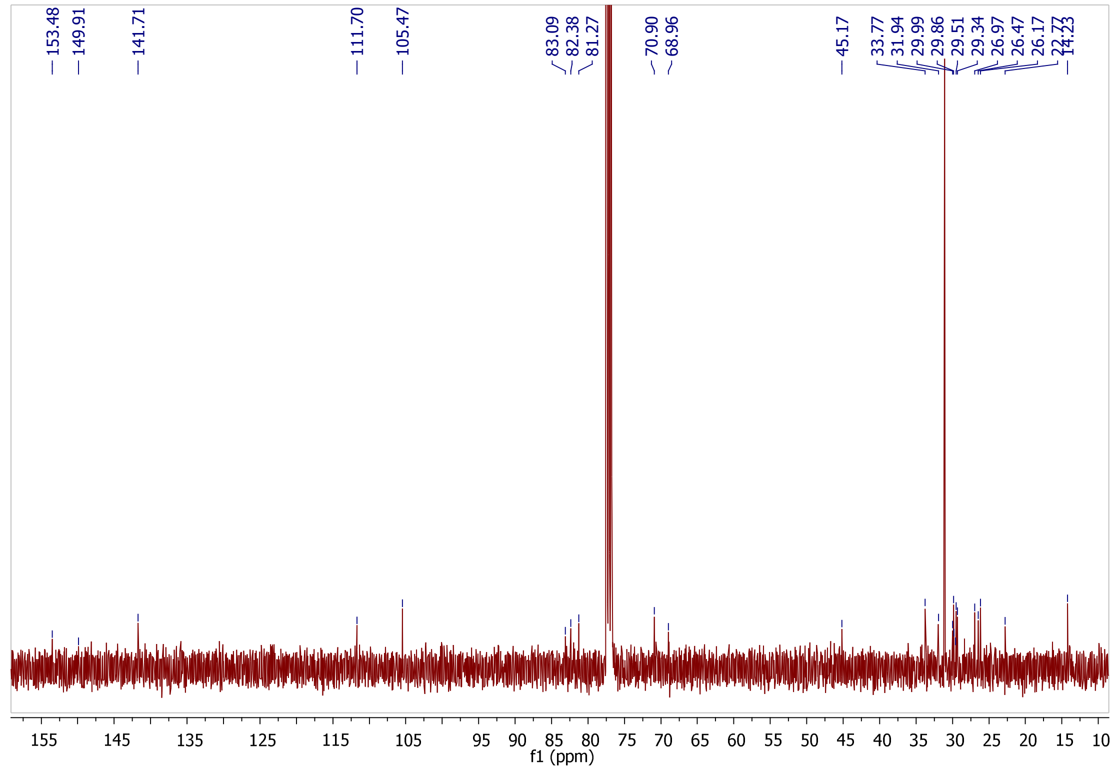

Figure S16 B. ^{13}C NMR Spectrum of compound **19**.

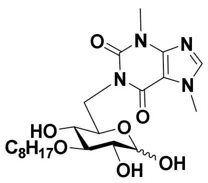

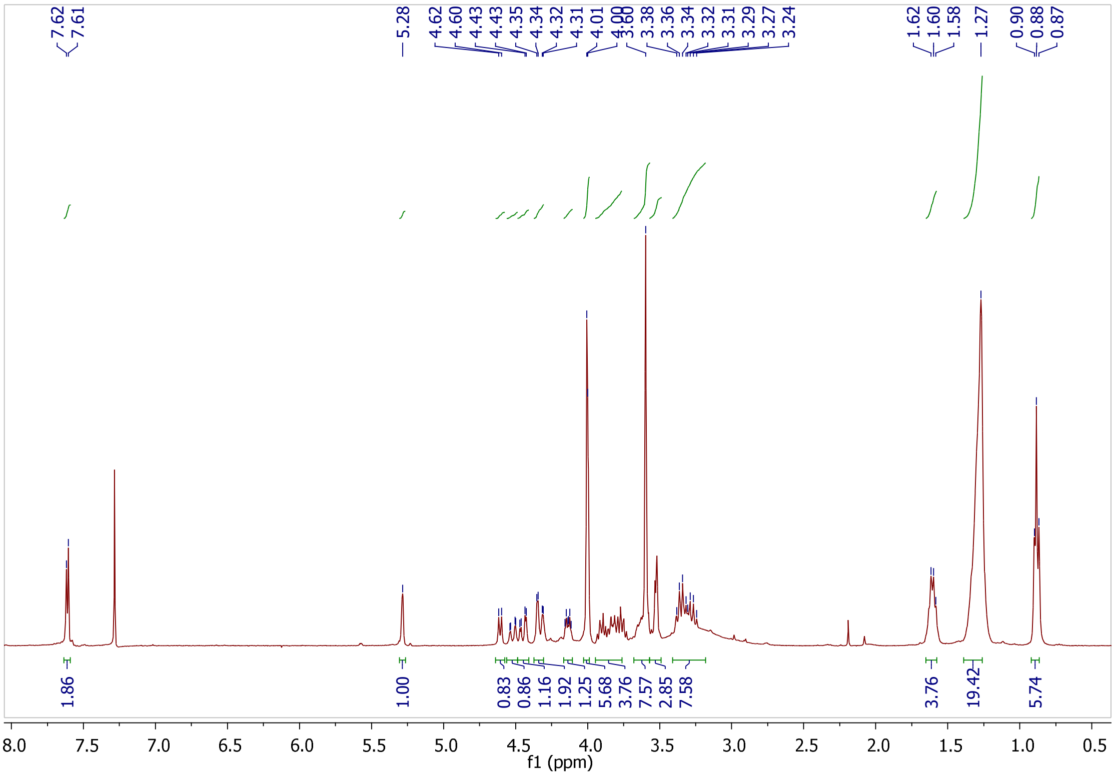

Figure S17 A. ¹H NMR Spectrum of compound **20**.

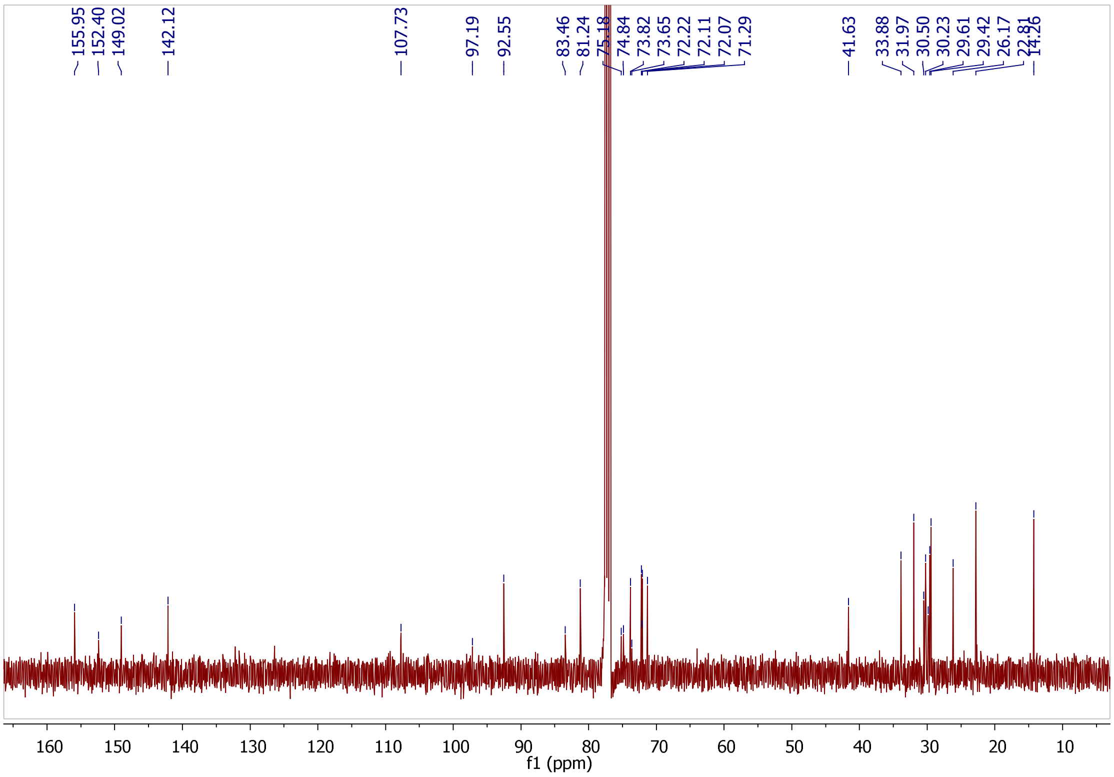

Figure S17 B. ¹³C NMR Spectrum of compound **20**.

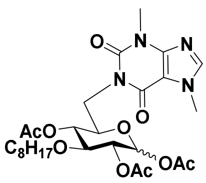

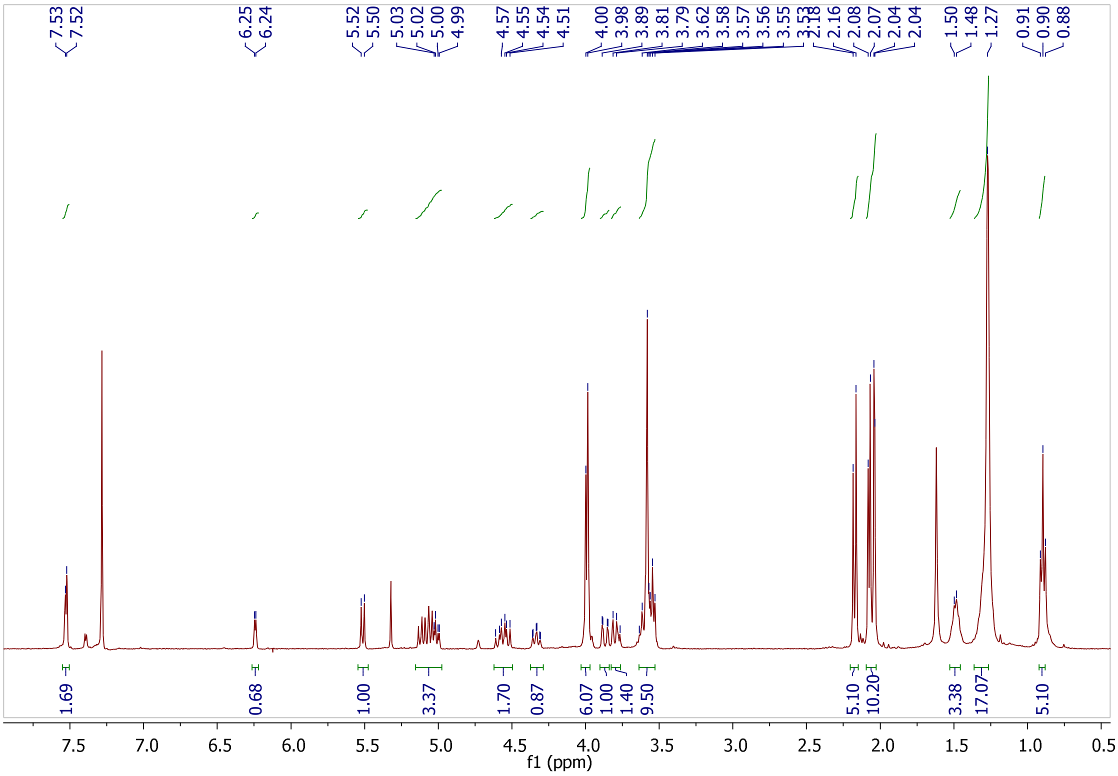

Figure S18 A. ^{1}H NMR Spectrum of compound **21**.

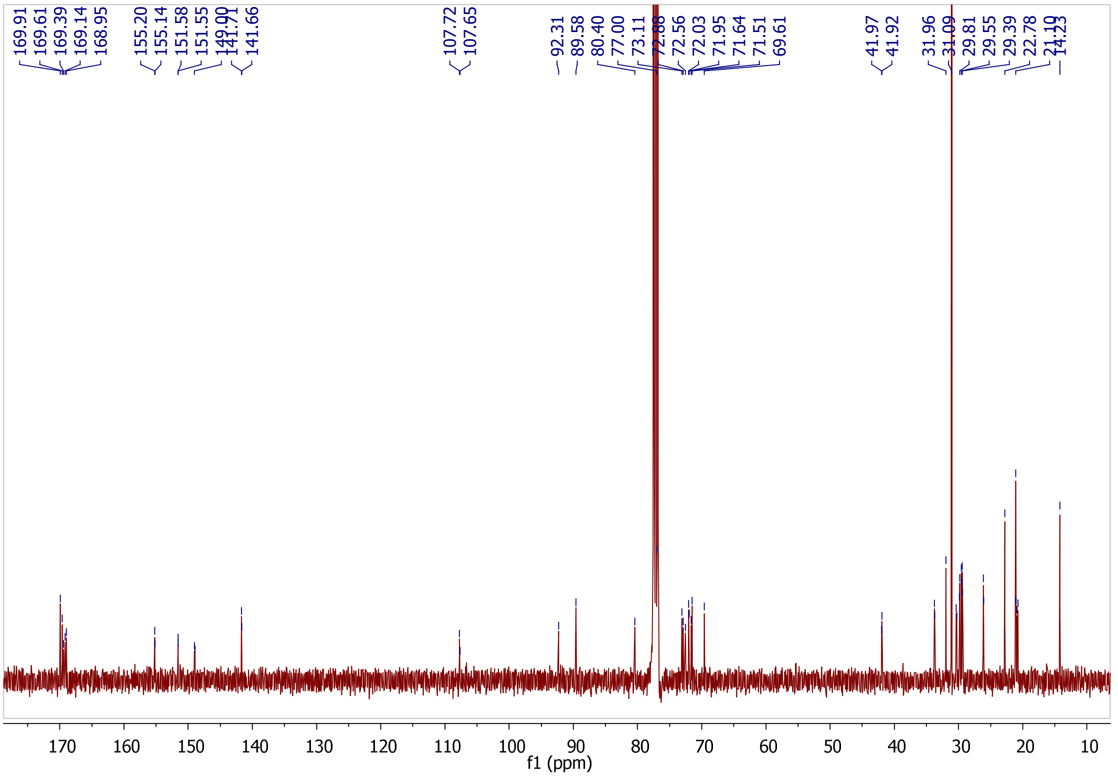

Figure S18 B. ^{13}C NMR Spectrum of compound **21**.

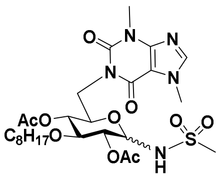

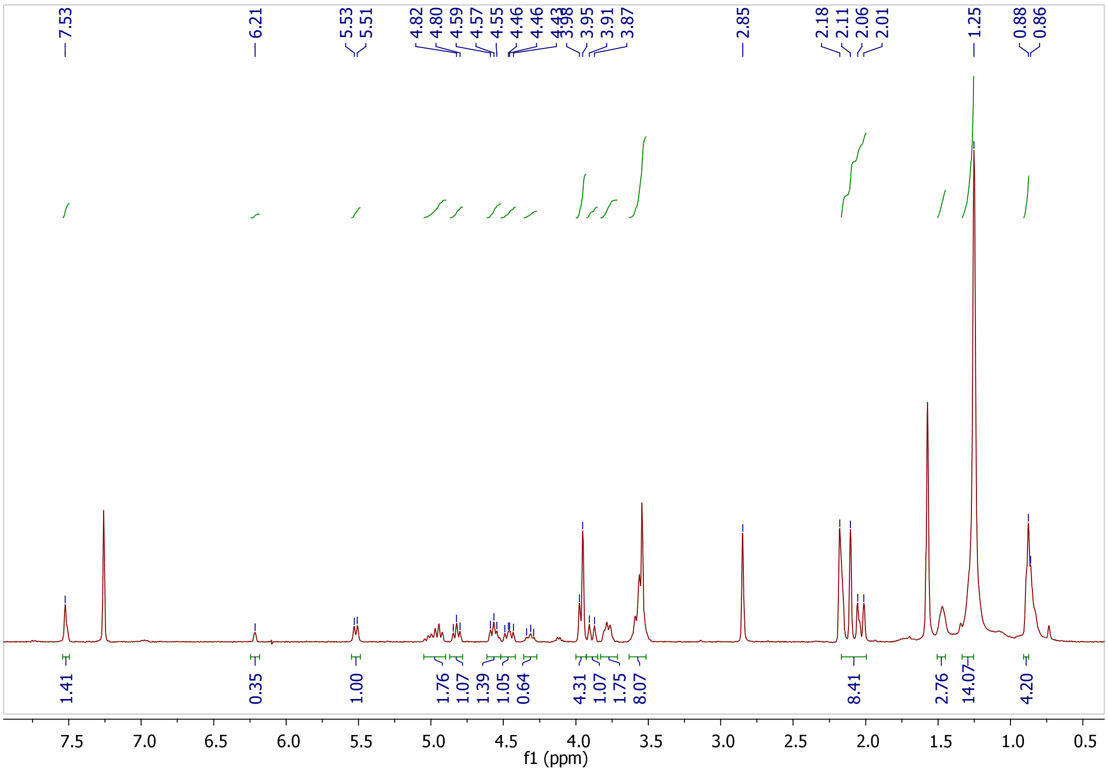

Figure S19 A. ¹H NMR Spectrum of compound **22**.

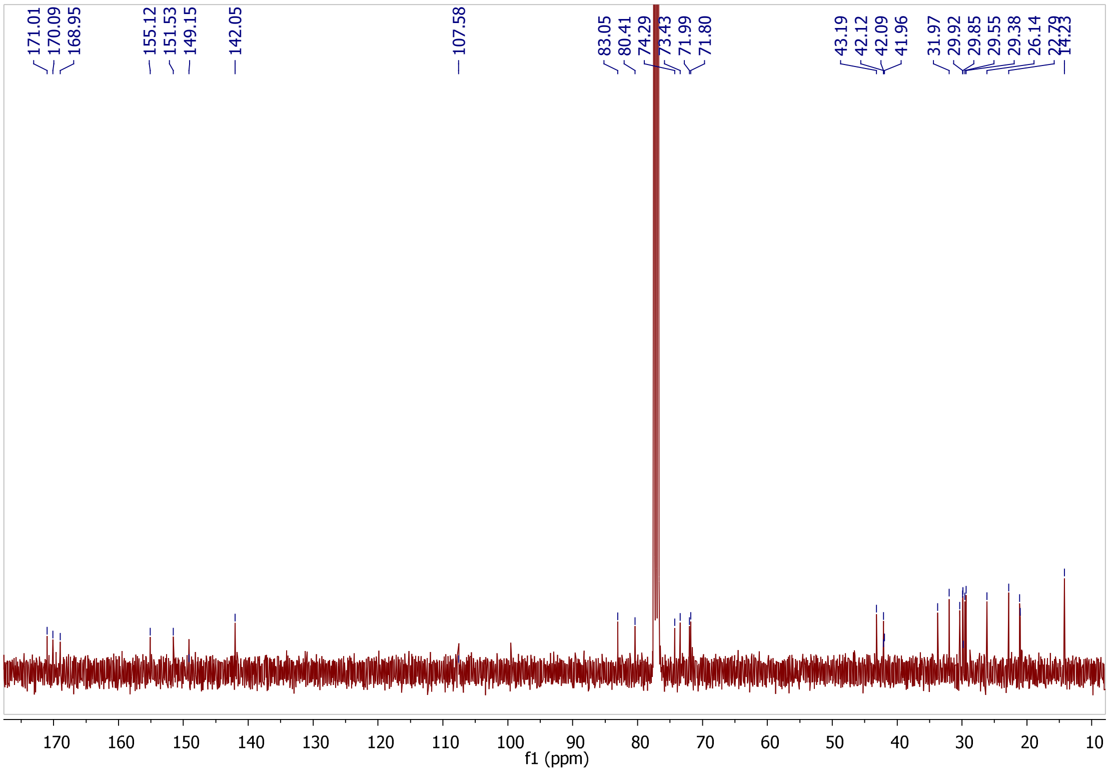

Figure S19 B. ¹³C NMR Spectrum of compound **22**.

2. Enzymatic Studies

Solutions preparation

Preparation of 50 mM Tris–HCl buffer solutions: Tris(hydroxymethyl)-aminomethan (606 mg) was dissolved in bi-distilled water (100 mL) and adjusted with HCl to a pH of 8.0 ± 0.1.
AChE solution 2.005 U/ml: the enzyme (271 U/mg, 0.037 mg) was dissolved in freshly prepared buffer (5 mL) containing NaN3 (0.98 mg).
BChE solution 2.040 U/ml: the enzyme (7.54 U/mg, 1.353 mg) was dissolved in freshly prepared buffer (5 mL) containing NaN3 (0.98 mg).
DTNB solution 3 mM: DTNB (23.8 mg) was dissolved in freshly prepared buffer (20 mL) containing NaCl (116.8 mg) and MgCl2 (38.0 mg).
ATChI solution 15 mM: ATChI (43.4 mg) was dissolved in bi-distilled water (10 mL).
All solutions were stored in Eppendorf caps in the refrigerator or freezer, if necessary. The pure compounds were initially dissolved in DMSO, galantamine hydrobromide as standard was dissolved in bi-distilled water. The final concentrations for the enzymatic assay were yielded by diluting the stock solution with bi-distilled water. No inhibition was detected by residual DMSO (<0.5%).

Enzyme assay

Inhibition constants

A mixture of the DTNB solution (125 µL), enzyme (25 µL) and compounds solutions (25 µL, 3 different concentrations and once water) was prepared and incubated at 30 °C for 20 min. The substrate (25 µL, 4 different concentrations) was added to start the enzymatic reaction. The absorbance data (λ = 415 nm) was recorded under a controlled temperature of 30 °C for 30 min at 1 min intervals. All measurements were performed as triplicates. The used substrate concentrations in the test were as follows: [ATChI] = 0.9375 mM, 0.625 mM, 0.325 mM, 0.1875 mM. The mode of inhibition as well as K_i and K_i' values were determined using Lineweaver–Burk, Dixon and Cornish-Bowden plots.

%-Inhibition

A mixture of the DTNB solution (125 µL), enzyme (25 µL) and compounds solutions (25 µL) was prepared and incubated at 30 °C for 20 min. The substrate (25 µL) was added to start the enzymatic reaction. The concentration of each compound was 50 µM. The used substrate concentration was 0.625 mM. The absorbance data (λ = 415 nm) was recorded under a controlled temperature of 30 °C for 10 min. The relative inhibition was determined as the quotient of the slopes (compound divided by blank) of the linear ranges.

3. Best Docking Poses for Compounds 9, 21-α and 21-β into BChE

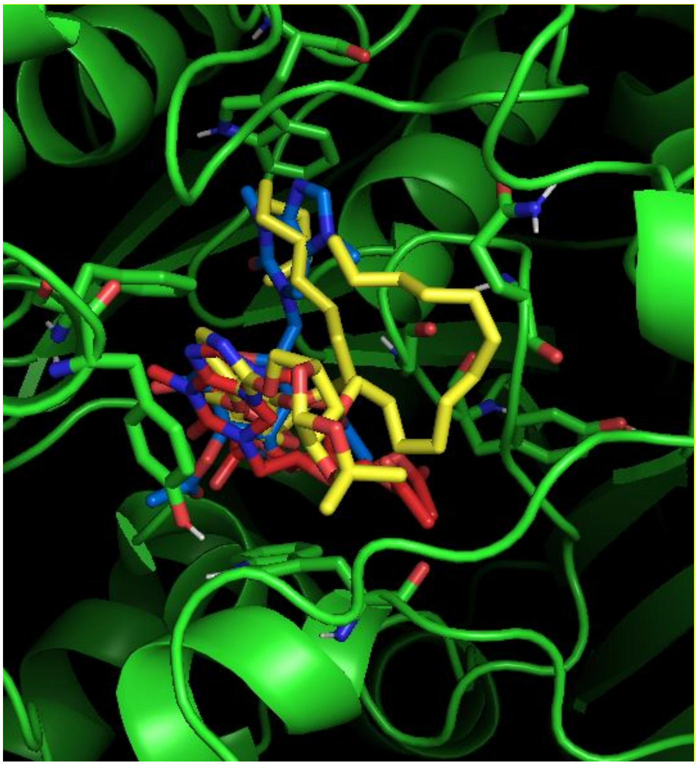

Figure S20. Lowest-energy binding poses of compounds **9** (yellow), **21-α** (blue) and **21-β** (red) to BChE.
